# Integrative proteogenomic characterization of early esophageal cancer

**DOI:** 10.1038/s41467-023-37440-w

**Published:** 2023-03-25

**Authors:** Lingling Li, Dongxian Jiang, Qiao Zhang, Hui Liu, Fujiang Xu, Chunmei Guo, Zhaoyu Qin, Haixing Wang, Jinwen Feng, Yang Liu, Weijie Chen, Xue Zhang, Lin Bai, Sha Tian, Subei Tan, Chen Xu, Qi Song, Yalan Liu, Yunshi Zhong, Tianyin Chen, Pinghong Zhou, Jian-Yuan Zhao, Yingyong Hou, Chen Ding

**Affiliations:** 1 State Key Laboratory of Genetic Engineering and Collaborative Innovation Center for Genetics and Development, School of Life Sciences, Institute of Biomedical Sciences, Human Phenome Institute, Zhongshan Hospital, Fudan University, Shanghai, 200433 China; 2 Department of Pathology, Zhongshan Hospital Fudan University, Shanghai, 200032 China; 3 Department of Oncology, The Affiliated Hospital of Southwest Medical University, Luzhou, 646000 China; 4 Endoscopy Center and Endoscopy Research Institute, Zhongshan Hospital Fudan University, Shanghai, 200032 China; 5 Institute for Development and Regenerative Cardiovascular Medicine, MOE-Shanghai Key Laboratory of Children’s Environmental Health, Xinhua Hospital, Shanghai Jiao Tong University School of Medicine, Shanghai, 200092 China; 6 Department of Anatomy and Neuroscience Research Institute , School of Basic Medical Sciences, Zhengzhou University, Zhengzhou, 450001 China

**Keywords:** Oesophagus, Proteomics, Oesophageal cancer, Cancer genomics

## Abstract

Esophageal squamous cell carcinoma (ESCC) is malignant while the carcinogenesis is still unclear. Here, we perform a comprehensive multi-omics analysis of 786 trace-tumor-samples from 154 ESCC patients, covering 9 histopathological stages and 3 phases. Proteogenomics elucidates cancer-driving waves in ESCC progression, and reveals the molecular characterization of alcohol drinking habit associated signatures. We discover chromosome 3q gain functions in the transmit from nontumor to intraepithelial neoplasia phases, and find *TP53* mutation enhances DNA replication in intraepithelial neoplasia phase. The mutations of *AKAP9* and *MCAF1* upregulate glycolysis and Wnt signaling, respectively, in advanced-stage ESCC phase. Six major tracks related to different clinical features during ESCC progression are identified, which is validated by an independent cohort with another 256 samples. Hyperphosphorylated phosphoglycerate kinase 1 (PGK1, S203) is considered as a drug target in ESCC progression. This study provides insight into the understanding of ESCC molecular mechanism and the development of therapeutic targets.

## Abstract (web summary)

The progression of oesophageal squamous cell carcinoma (ESCC) from early to advanced stages requires comprehensive molecular characterisation. Here, the authors perform a proteogenomics analysis of ESCC patient samples across nine histopathological stages and three phases, identifying key alterations and paths for progression.

## Introduction

Esophageal cancer (EC) is a malignant gastrointestinal carcinoma, ranking the seventh most common cancer and the sixth leading cause of cancer-related death worldwide^1^. Esophageal adenocarcinoma (EAC) and esophageal squamous cell carcinoma (ESCC) are the two major histologic subtypes of EC, of which EAC is more prevalent in western countries^2^, whereas ESCC predominantly occurs in East Asia, particularly in China and Japan^3^, indicating the diverse lifestyle of the countries as a major etiological factor of EC. The other etiological factors of EC include gender, age^4^, and the habits of drinking/smoking^5^. However, the molecular signatures, which are associated with the risk factors in EC progression, are still unknown.

Generally, the carcinogenesis of tumor development hall long-drawn-out process, and genome data act as a “fossil” record of how a tumor came to be. Several large-scale ESCC cohorts, including The Cancer Genome Atlas (TCGA)^6^, presented genomic aberrations and identified highly mutated genes (e.g., *TP53*) in the advanced stages of ESCC. However, the first occurrences of the mutations/key events and the related effects during the carcinogenesis of ESCC are poorly understood. Liu et al. showed that earlier and advanced stages of ESCC shared several significantly mutated genes^7^. Thus, the mutations were cumulative in ESCC progression, and the key mutation/events which were found in the advanced stages might exist in the earlier stages, whereas there is still a lack of proteogenomic landscape in ESCC progression. In addition, the molecular mechanism of the diversity and tumor heterogeneity of ESCC is poorly understood, imposing a challenge for developing ESCC therapeutic strategies.

Histologically, the esophageal wall includes the epithelium, lamina propria, muscularis mucosa, submucosa, muscularis externa, and adventitia^8^. The depth of infiltration of the cancer cells, determining the stage of lesions, was measured at the deepest point of their penetration in corresponding layers^9^. Surgery is the predominant curative treatment strategy in advanced stages where the cells invade muscularis externa (T2–T4 stages), with poor quality of life (QOL) and a low 5-year survival rate (<30%)^10^. Though the recent advances in endoscopic submucosal dissection (ESD)^11^ have achieved the early detection of ESCC patients (T1 stage) with higher QOL and significantly improved overall survival rate (>90%)^12^, the complexity of the early ESCC progression and the extreme trace amount of tissue samples in different stages have limited in portraying the multi-omics molecular landscape of ESCC.

In this study, we perform a comprehensive multi-omics analysis of 786 trace-tumor samples from 154 ESCC patients. The integrative multi-omics dataset reveals the stage-specific and risk factor-associated molecular characterization, and defines the cancer-driving waves along with the mutation accumulation in EC progression. Proteogenomics uncovers key events in the transit of the phases and the trajectory analysis shows 6 major tracks and their molecular characteristics during the carcinogenesis of ESCC.

## Results

### Overview of proteogenomic landscape in ESCC progression

**Fig. 1:**
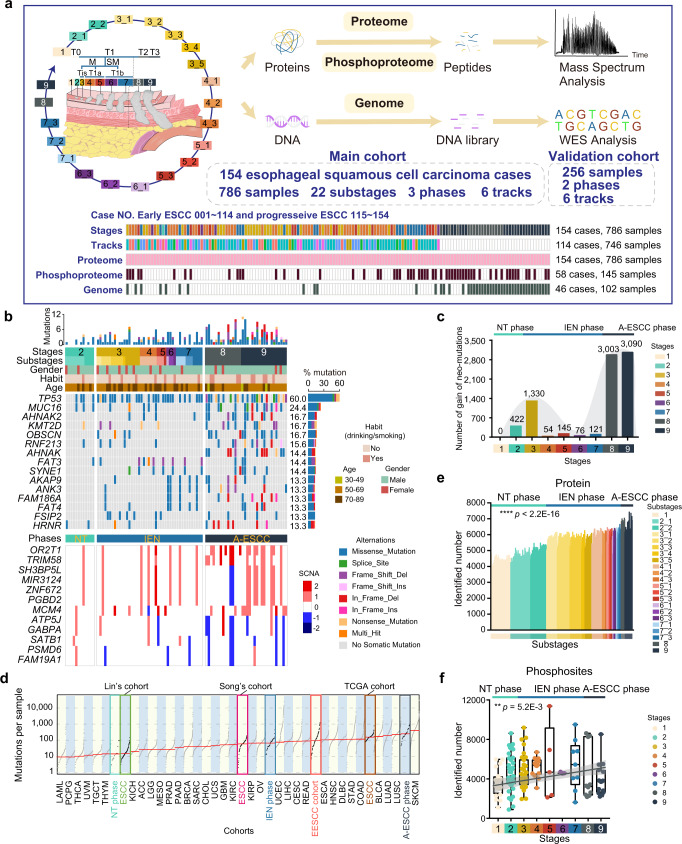
The multi-omics landscape in ESCC progression. **a** Overview of the experimental design and the number of samples for the genomic, proteomic, and phosphoproteomic analyses. ESCC esophageal squamous cell carcinoma. **b** The genomic profile of ESCC progression. Top: the mutation number and types of all the samples from early to progressive ESCC. Bottom: the somatic copy number alterations of all the samples from early to progressive ESCC. The mutation frequencies are shown by a bar plot at the right panel. NT phase: non-tumor phase, IEN phase: intraepithelial neoplasia phase, A-ESCC phase: advanced-stage ESCC phase. **c** The gain of neo-mutations at all stages in ESCC progression. **d** Analysis of the mutations loads of diverse cohorts. EESCC cohort: early ESCC cohort. **e** The number of the identified proteins of 786 samples (Kruskal–Wallis test, *p* < 2.2E–16). **f** Boxplot showing the number of the phosphosites identifications of 145 samples (Kruskal–Wallis test). Boxplot shows median (central line), upper and lower quartiles (box limits), 1.5× interquartile range (whiskers). ****p < 1.0E–4, ***p < 1.0E–3, **p < 0.01, *p < 0.05, ns. > 0.05. Source data are provided as a Source data file.

We performed multi-omics-based profiling of trace 786 samples collected from 154 ESCC patients who had not experienced prior chemotherapy or radiotherapy. The clinicopathological characteristics of patients and tumors are summarized in Supplementary Table 1. Subsequently, 22 substages during ESCC progression from healthy esophageal tissue to tumor development were established for these samples following WHO and Japanese pathology diagnostic criteria^13^. The number of substages identified from the ESCC patients varied from 4 to 16, and the tissue samples from the corresponding substages were separately dissected from the formalin-fixed, paraffin-embedded (FFPE) slides (Supplementary Fig. 1a). All ESCC samples in our cohort were dissected 3 mm thick and stood up one by one in the embedding, and were then marked in the hematoxylin and eosin (H&E)-stained sections (Supplementary Fig. 1c, d). In total, 786 samples were collected and classified into 9 histopathological stages covering 22 substages in our cohort (Supplementary Table 2), including stage 1 (normal tissue stage), stage 2 (hyperplasia stage), stage 3 (Tis stage), stage 4 (lamina propria cancer stage), stage 5 (muscularis mucosa stage), stage 6 (submucosal invasion cancer stage a), stage 7 (submucosal invasion cancer stage b), stage 8 (T2 stage), and stage 9 (T3 stage) (Fig. 1a and Supplementary Data 1a; Methods). The normal (stage 1)/tumor cell purity of all samples was over 95%, indicating the high quality of all samples of our cohort (Supplementary Fig. 1e). In addition, we collected another 256 samples as an independent validation cohort from 49 early-stage ESCC patients, covering 6 of 9 histopathological stages as stages 1, 2, 3, 4, 5, and 7 (Fig. 1a and Supplementary Table 3; Methods). That represented the advantages of our samples in a pathological region-resolved mode, providing the chance to portray molecular profiles of ESCC in a time-resolved mode. Subsequently, we performed mass spectrometry (MS) profiling of all 786 samples, phosphoproteomic profiling of 145 samples (Supplementary Table 4 and Supplementary Data 1b), and whole-exome sequencing (WES) of 102 samples in the main cohort (Fig. 1a, Supplementary Figs. 1b and 2a, and Supplementary Data 1c, d; Methods). To demonstrate the findings and results in the main cohort, we also performed proteomic profiling of 256 samples in the validation cohort.

WES profiling identified 9547 mutations in the Fudan cohort (this study) (Supplementary Data 2a). The top mutations were *TP53*, *MUC16*, *FAT3*, *SYNE1*, *AKAP9*, *FAT4*, etc. (Fig. 1b). *TP53*, frequently mutated in EC^14^, was the top-ranked mutation in the Fudan cohort, and was co-occurrence with the mutation of *KMT2D* (Supplementary Fig. 2b). In our study, we observed that the number of mutations gradually cumulated during ESCC carcinogenesis, ranging from 422 mutations in hyperplasia stage to 5280 mutations in the T3 stage. Neo-mutations were indicated as those just appearing at a certain stage (Methods). In our cohort, we found that the number of the neo-mutations peaked at the Tis stage (*n* = 1330), and the T2 (*n* = 3003), indicating the significant events during carcinogenesis (Fig. 1c). Thus, nine pathological stages of our cohort were grouped into three phases (NT, IEN, and A-ESCC) (Fig. 1c), which allowed us to explore the key mutational events and the corresponding impacts in ESCC progression. Observation of mutation loads of the Fudan cohort and other ESCC cohorts (TCGA cohort^15^, Lin’s cohort^16^, and Song’s cohort^17^) showed the lowest mutation loads in the NT phase in the Fudan cohort (Fig. 1d). Liu et al. reported fewer mutations were detected in the esophageal nondysplastic epithelium (simple hyperplasia)^7^, which was also observed in our cohort that the mutation loads of the NT phase were lower (Wilcoxon signed-rank test, *p* < 0.05, A-ESCC vs. NT ratio = 9.36, IEN vs. NT ratio = 2.08) (Supplementary Fig. 2c), indicating the low mutation burden of our cohort was due to the low mutation loads of the early-stages of ESCC. Collectively, we built a comprehensive genomic landscape in ESCC progression, and presented the difference between the early and advanced stages of ESCC.

At the protein and phosphoprotein levels, proteomic analysis was performed using a label-free quantification strategy^18^. Protein abundance was first calculated by intensity-based absolute quantification (iBAQ)^19^ and then normalized as the fraction of total (FOT). Spearman’s correlation coefficient of HEK293T cells was 0.91, indicating the consistent stability of our MS platform (Supplementary Fig. 2d and Supplementary Data 3a). The gradually decreased (Spearman’s) correlation coefficient of the nine histopathological stages reflected the increased tumor heterogeneity during the carcinogenesis of ESCC, highlighting the importance of exploring molecular characteristics in ESCC progression (Supplementary Fig. 2e). With the advancement of the stages of ESCC, the numbers of protein identifications were slightly increased (Kruskal–Wallis test, *p* < 2.2E–16) from ~5000 in stage 1 (normal tissue) to ~7000 in stages 8 and 9 (T2 and T3 stages) with a total of 15,071 in 786 samples at 1% false discovery rate (FDR) (Methods) (Fig. 1e and Supplementary Fig. 2f). In addition, the reference proteome was highly dynamic based on the protein abundance, which spanned over eight orders of magnitude (Supplementary Fig. 2g). In addition, the ESCC biomarkers identified in previous studies (several ESCC tissues or cell lines), including ACTA2, TAGLN^20^, HSPA9, PDIA4, PLEC, POSTN, PSAP, and THBS1^21^ were also covered in the Fudan cohort (Supplementary Data 3b). At the phosphoprotein level, a total of 52,856 phosphosites corresponding to 7612 phosphoproteins were identified in 145 samples (Supplementary Fig. 2h, i and Supplementary Data 3c). During the process of ESCC carcinogenesis, the number of phosphosite identifications was slightly increased (Kruskal–Wallis test, *p* = 5.2E–3) (Fig. 1f). In the validation cohort, label-free quantification measurement of 256 samples resulted in a total of 12,383 protein groups with a 1% FDR at the protein and peptide levels (Supplementary Fig. 2j). Consistent with the findings in the main cohort, with the advancement of the stages of ESCC, slightly elevated protein identifications were detected in the validation cohort (Kruskal–Wallis test, *p* < 1.0E–4) (Supplementary Fig. 2k). Compared to the NT phase, more protein identifications were identified in the IEN phase both in the main cohort and validation cohort (Wilcoxon rank-signed test, *p* < 1.0E–4) (Supplementary Fig. 2l). Overall, we established a comprehensive landscape of ESCC progression at the multi-omics level.

### The SBS16 signature, associated with alcohol drinking, promoted DNA replication in the IEN phase

Except diverse lifestyle of the countries^2,3^, alcohol drinking is one of the key risk factors of ESCC^22^. Moody et al. pointed that SBS16 is the significant signature associated with alcohol drinking^22^, whereas the impacts of SBS16 signature on the molecular level in the ESCC progression have not been revealed. Furthermore, SBS16 signature shows a negative association with the overall survival of ESCC patients (referred from TCGA cohort, log-rank test, *p* = 0.049) (Supplementary Fig. 3a). These findings allowed us to explore the impacts of SBS16 in ESCC patients with drinking habit at the molecular level.

**Fig. 2:**
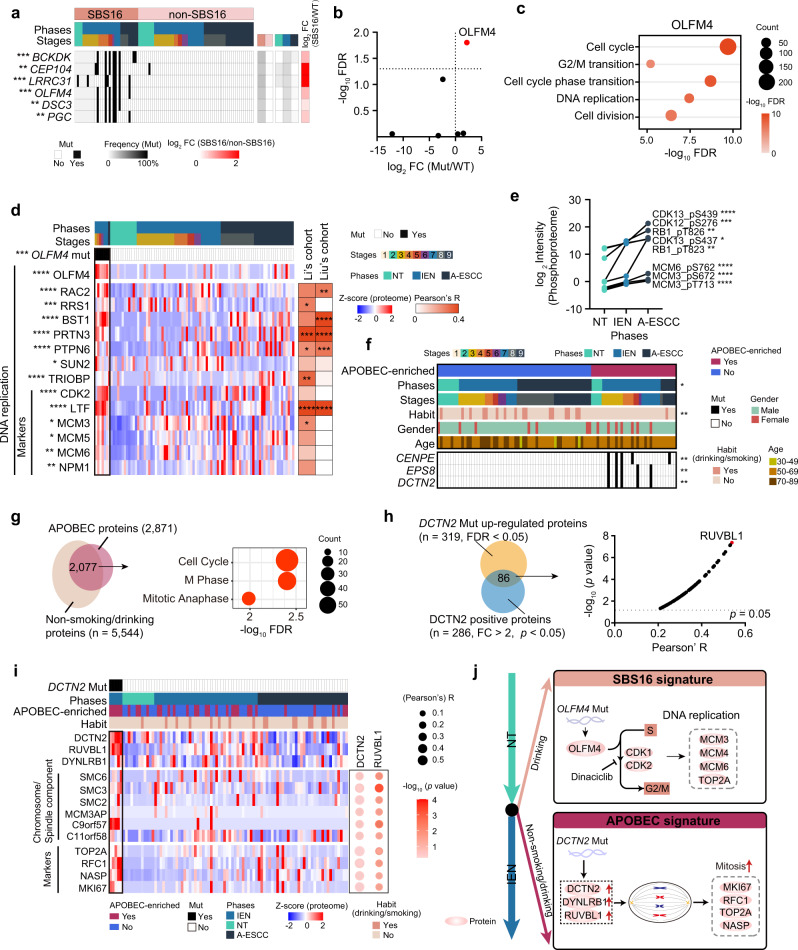
The risk factor-associated mutational signatures in ESCC progression. **a** Heatmap showing the significantly positive associated mutations in SBS16 signature (two-sided Fisher’s exact test). ***p = 1.4E–4 (*BCKDK*), **p = 2.7E–3 (*CEP104*), ***p = 1.4E–4 (*LRRC31*), ***p = 4.5E–4 (*OLFM4*), ***p = 1.5E–3 (*DSC3*), ***p = 1.5E–3 (*PGC*). The square directs to a subset of patient samples used for WES. **b** Volcano analysis of the impacts of significantly positive associated mutations in SBS16 signature on their counterpart proteins expression (two-sided Wilcoxon signed-rank test). **c** Represented pathway enrichment that was positively correlated with OLFM4. **d** Heatmap showing the protein levels of DNA replication in *OLFM4* mutation group vs. WT group (two-sided Wilcoxon signed-rank test, BH-adjusted *p < 0.05). **e** The expression (log_2_-transformed Intensity, median) of represented DNA replication-related phosphoprotein in ESCC progression (Kruskal–Wallis test, BH-adjusted *p < 0.05). A total of 145 samples for phosphoproteomic profiling are used in this analysis. *n* (NT) = 57, *n* (IEN) = 62, *n* (A-ESCC) = 26 biologically independent samples examined. **f** The correlation between etiological factors (top) and the significantly associated mutations of APOBEC signature (bottom) (two-sided Fisher’s exact test). *p = 0.012 (Phase), **p = 4.2E–3 (Habit), **p = 4.6E–3 (*DCTN2*), **p = 4.6E–3 (*EPS8*), **p = 4.6E–3 (*CENPE*). **g** Venn diagram depicting the number of the overlapped proteins overrepresented in the APOBEC signature and non-smoking/drinking ESCC patients (left, two-sided Wilcoxon signed-rank test, BH-adjusted *p < 0.05), and the associated signaling pathways (right). **h** Venn plot (left) showing the overlapped proteins significantly correlated with DCTN2 at the gene and protein levels (two-sided Wilcoxon signed-rank test, BH-adjusted *p < 0.05). Volcano plot (right) de*p*icting the correlation between DCTN2 and the overlapped proteins (*n* = 86, two-sided Pearson’s correlation test). **i** Heatmap (left) presenting the represented chromosomal/spindle components and cell proliferation markers overrepresented in the *DCTN2* mutation group (two-sided Wilcoxon signed-rank test), and correlation (right) between DCTN2/RUVBL1 and represented chromosomal/spindle components and cell proliferation markers (two-sided Pearson’s correlation test). **j** A brief summary of the impacts of the SBS16 signature (top) and APOBEC signature (bottom) in ESCC progression. A total of 102 samples for WES are used in the analysis. ****p < 1.0E–4, ***p < 1.0E–3, **p < 0.01, *p < 0.05, ns. > 0.05. Source data are provided as a Source data file.

Of note, gene ontology (GO) analysis revealed that DNA replication was enriched in the ESCC patients with SBS16 signature (Supplementary Fig. 3b). Among the mutations, significantly related to SBS16 signature, we found that *OLFM4* mutation upregulated its corresponding protein expression (Wilcoxon signed-rank test, *OLFM4* Mut vs. WT ratio = 4.6, FDR = 3.4E–5) (Fig. 2a, b). Generally, OLFM4 promotes S-phase transition in cancer cell proliferation^23^. In our cohort, OLFM4-positive-associated proteins were involved in cell proliferation, including DNA replication, cell division, etc. (Fig. 2c, d). Moreover, OLFM4 displayed a positive association with CDK1 and CDK2 at the protein level (Pearson’s *R* = 0.44 for CDK1 and 0.54 for CDK2, *p* = 0.013 for CDK1 and 2.5E–3 for CDK2) (Supplementary Fig. 3c), which is a key regulator in the transition from S-phase to G2/M phase in cell cycle^24^. The mutation of *OLFM4* showed positive impacts on the protein levels of cell proliferation-related markers, such as CDK2, MCM3/5/6, etc. (Fig. 2d). Besides, over-phosphorylation of DNA replication-related proteins was also detected during the carcinogenesis of ESCC (Kruskal–Wallis test, FDR < 0.05, A-ESCC and IEN vs. NT ratio ≥ 2) (Fig. 2e). To further validate the findings in our cohort, we analyzed the impacts of OLFM4 in other ESCC cohorts, such as the Li’s cohort^25^ and the Liu’s cohort^26^, and found that OLFM4 displayed positive association with DNA replication-related proteins at the protein level in these cohorts (Fig. 2d and Supplementary Fig. 3d). Taken together, SBS16 signature was a key event in the transition from the NT phase to the IEN phase for ESCC patients with a drinking habit, in which *OLFM4* mutation showed positive impacts on DNA replication (Fig. 2j).

### The APOBEC signature was overrepresented in the non-smoking/drinking ESCC patients

To explore the specific etiological factors that might contribute to the mutagenesis of ESCC, we adopted a non-negative matrix factorization (NMF) algorithm^27^ to extract mutational signatures from the WES data of the Fudan cohort and other ESCC cohorts, such as Moody’s cohort^22^, TCGA cohort^15^, Lin’s cohort^16^, and Song’s cohort^17^. In our cohort, DNA repair signature was detected as early as in the NT phases and lasted till the A-ESCC phase (Fisher’s exact test, *p* = 2.8E–3, 41.7%, 31.8%, 70.6% for NT/IEN/A-ESCC phases, respectively) (Supplementary Fig. 3e, f), indicating DNA damage is one of the causes leading to esophageal carcinogenesis. In addition, we found APOBEC signature was prominent in ESCC progression and was specially detected as early as in the IEN phase (Fisher’s exact test, *p* = 0.012, 66.7%) (Fig. 2f and Supplementary Fig. 3g). Similarly, we confirmed that APOBEC signature was detected in the earlier stage of ESCC (Fisher’s exact test, *p* = 0.029, 50%) in the TCGA cohort^28^ (Supplementary Fig. 3h).

Integration of the findings in the Moody’s cohort^22^, we found APOBEC signature was prominent in the non-smoking/drinking ESCC patients (Fisher’s exact test, *p* = 8.4E–3, 42.5%), in which higher enrichment score was detected (Wilcoxon signed-rank test, FDR = 5.1E–4, non-smoking/drinking vs. smoking vs. drinking ratio = 1.78) (Supplementary Fig. 3i, j). To explore the impacts of APOBEC signature in the non-smoking/drinking ESCC patients, we integrated the highly expressed proteins both detected in the APOBEC signature and ESCC patients with non-smoking/drinking habit, and discovered these proteins were correlated with cell proliferation process (Fig. 2g). Somatic mutations analysis revealed that the mutations of *CENPE*, *EPS8*, and *DCTN2*, were positive associated with APOBEC signature (Fisher’s exact test, *p* < 0.05) (Fig. 2f). We further investigated the impacts of these three mutations, and found *DCTN2* mutation upregulated its corresponding protein expression (Wilcoxon signed-rank test, FDR = 0.040, Mut vs. WT ratio = 2.0) at the protein level (Supplementary Fig. 3k). In addition, only the protein level of DCTN2 was significantly overrepresented in the APOBEC signature group (Wilcoxon signed-rank test, FDR = 5.1E–5) (Supplementary Fig. 3k). These findings indicated the potential effects of *DCTN2* mutation in the patients with APOBEC signature.

DCTN2, a subunit of dynactin, binds to both microtubules and cytoplasmic dynein, and is involved in a diverse array of cellular functions, including spindle formation, chromosome movement, nuclear positioning, etc.^29^. Among the dynein families, we found that DYNLRB1 exhibited a positive correlation with DCTN2 (Pearson’s *R* = 0.30, *p* = 4.3E–3) at the protein level (Supplementary Fig. 3l). Dynein families share a conserved motor domain coupled cycles of ATP hydrolysis with conformational changes to produce movement^30^. To determine the activation and movement of dynein in mitosis, we integrated DCTN2 positively correlated proteins, and found RUVBL1 showed a significant correlation with DCTN2 (Pearson’s *R* = 0.54, *p* = 4.3E–8) at the protein level (Fig. 2h). Generally, RUVBL1 exhibits DNA- and nucleosome- activated ATPase activity and catalyzes ATP-dependent nucleosome sliding in mitosis^31,32^. In our cohort, we found *DCTN2* mutation had positive impacts on the protein level of chromosomal/spindle components, evidenced by the markers including SMC2, SMC3, etc. (Fig. 2i). As well as RUVBL1, DCTN2 showed a significantly positive correlation with chromosomal/spindle components and markers of tumor cell proliferation (e.g., TOP2A, MKI67, etc.)^33^ (Fig. 2i). These findings indicated the activation of microtubule and spindle in G2/M in the mitosis. Taken together, the links of the findings in our cohort and the Moody’s cohort revealed that APOBEC signature was prominent in the ESCC patients with non-smoking/drinking habit, in which *DCTN2* mutation upregulated the protein level of DCTN2, elevating mitosis and cell proliferation (Fig. 2j).

### The gain of chromosome 3q was a key event in the transmit from the NT phase to the IEN phase

**Fig. 3:**
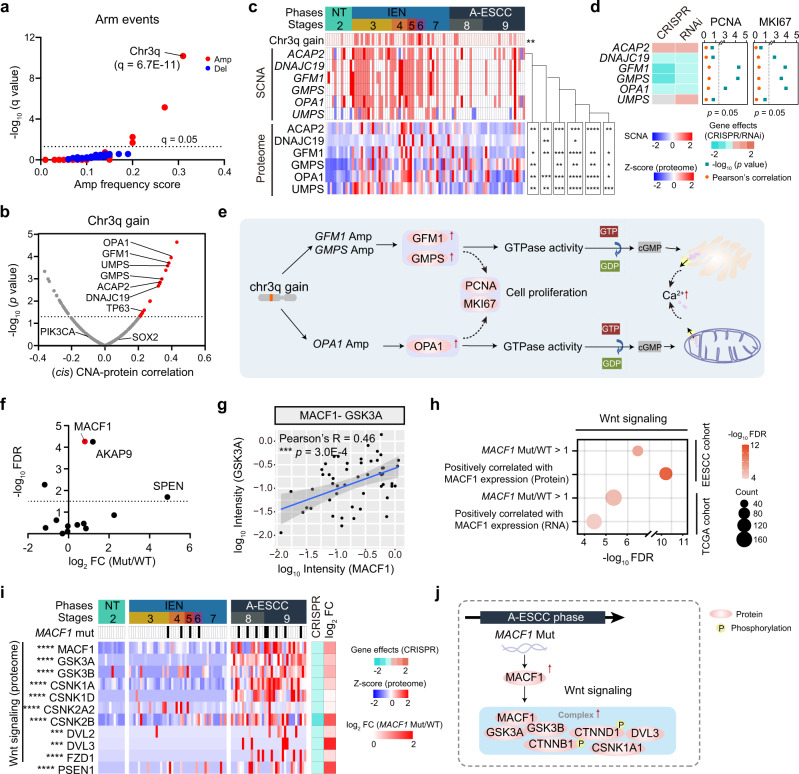
Integrative omics analyses of early ESCC samples. **a** The significant arm events of 102 samples in ESCC progression. The gain and loss events are highlighted in red and blue, respectively. **b** Volcano plot showing the *cis*- correlation of the SCNA (*x*-axis) and the associated –log_10_ (*p* value) (*y*-axis) on the genes at chr3q gain (two-sided Spearman’s correlation test). **c** The *cis* SCNA-protein regulations of significantly correlated genes (top) on their corresponding proteins expression (bottom) (two-sided Spearman’s correlation test, *p* < 0.05). **d** Dependency map-supported (https://depmap.org) panels showing relative survival averaged across all available ESCC cell lines after depletion of the indicated genes by RNAi or CRISPR. The right shows Pearson’s correlation and *p* value of these genes with PCNA and MKI67 at the protein level (two-sided Pearson’s correlation test). **e** A brief summary of the impacts of chr3q gain. **f** Volcano plot showing the impacts of the top ten mutations of ESCC progression on proteins expression (log_2_-transformed Intensity) (*x*-axis) and the associated –log_10_ (FDR) (*y*-axis) (two-sided Student’s *t*-test). **g** Scatterplot showing the relationship between log_10_ GSK3A and log_10_ MACF1 expression at the protein level in the Fudan cohort (two-sided Pearson’s correlation test, mean ± SD). **h** Represented pathway enrichment of proteins that was positively correlated with MACF1 in the Fudan cohort (top) and the TCGA ESCC cohort (bottom). Biological pathways are analyzed from the GO/KEGG database. **i** Heatmap showing the impacts of the mutation of *MACF1* on the expression of Wnt signaling-related proteins in ESCC progression (Kruskal–Wallis test, BH-adjusted *p < 0.05). The square directs to a subset of patient samples used for WES (*n* = 102). **j** A brief summary of the impacts of the mutation of *MACF1*. ****p < 1.0E–4, ***p < 1.0E–3, **p < 0.01, *p < 0.05, ns. > 0.05. Source data are provided as a Source data file.

To explore the impacts of key arm events, we performed whole exome-based somatic copy number alterations (SCNAs) analyses based on WES data and examined the regulatory effects of 23,109 SCNAs on protein expressions of 102 samples in ESCC progression (Supplementary Data 3d). The integrated genomic data of all phases illustrated that the gain of chromosomes 3q (chr3q), which was also observed in the TCGA ESCC cohort, was the top-ranked arm event, which was prominent in the IEN phase (Fisher’s exact test, *p* = 2.9E–3, 45.5%) (Fig. 3a and Supplementary Fig. 4a–c). In addition, the individual regions at the chr3q gain also showed significant association in ESCC progression, including chr3q26.1 gain, chr3q29 gain, chr3q22.1 gain, and chr3q12.2 gain (Supplementary Fig. 4d). To explore the biological functions of chr3q gain, we performed *cis-*effects analysis of the genes with CNA regions at the protein level, and found 20 genes at chr3q gain perturbation profiles had significantly positive *cis* effects on their associated proteins (Fig. 3b). Interestingly, nearly 35% genes at chr3q gain were positively associated with Ca^2+^ signal.

The Ca^2+^ signal is identified as a key regulator in processes of excitable cells (e.g., neurons) and non-excitable cells (including those of the epithelia)^34^, where it controls a diverse array of processes such as secretion, proliferation^35^, and promotes cancer cell survival^36^. In our cohort, all the amplifications of genes in the Ca^2+^ signal were detected in the IEN phase (Fig. 3c), suggesting the gain of chr3q event was detected as early as in the ESCC early stage. In addition, we found the amplifications of those genes also had significantly positive effects on other parallel proteins in the Ca^2+^ signal at the protein level, such as the amplification of *GMPS* was positively associated with the protein levels of GFM1, OPA1, etc. (Fig. 3c). To assess the effects of these amplified genes, we annotated outliers for the degree of which CRISPR- or short hairpin RNA (RNAi)-mediated depletion reduced ESCC cell lines^37^. As a result, we found the deletion of three genes (*GMPS*, *GFM1*, and *OPA1*) had negative effects on the proliferation of ESCC cell lines (Fig. 3d and Supplementary Data 4a, b). In addition, the three genes (*GMPS*, *GFM1*, and *OPA1*) exhibited a positive correlation with PCNA and MKI67, makers of tumor cell proliferation^33^ (Fig. 3d).

Furthermore, we found that the other important genes at chr3q (e.g., *TP63/PIK3CA/SOX2*) were also prominent in the IEN phase (Fisher’s exact test, *p* < 0.05, 59.1%, 70.9%, 59.1% for TP63/PIK3CA/SOX2 respectively) (Supplementary Fig. 4e). Specifically, the amplifications of *TP63* at chr3q28 showed positive impacts on DNA replication (Supplementary Fig. 4f, g), further indicating the functions of chr3q gain in the transmit process from the NT phase to the IEN phase, and implying the impacts of *TP63* amplification on DNA replication in ESCC progression. Taken together, the chr3q gain was a driven event in the transmit process from the NT phase to the IEN phase, leading to the activation of Ca^2+^ signal and cell proliferation in the IEN phase (Fig. 3e).

### The impacts of genomic aberrations in ESCC progression

Notably, *TP53*, highly mutated in ESCC^7^, was the top-ranked mutation in ESCC progression, and was prominent in the younger male ESCC patients (Fisher’s exact test, *p* = 0.044, 65.8%) in our cohort (Supplementary Fig. 4h). To explore the impacts of *TP53* mutation in ESCC progression, we incorporated the alterations of significantly upregulated proteins (SUPs, Wilcoxon rank-signed test, FDR < 0.05) (Supplementary Fig. 4i). As well as in the TCGA cohort^15^, the GO enrichment disclosed that those overlapped SUPs were related to DNA replication and cell cycle (e.g., MCM3/4/5/6, CCNK, PPP1R8, etc.), and ECM signaling (e.g., ITGA2/3/5, LAMA3/5, etc.), which was also overrepresented in the tumor tissues compared to the paired non-cancerous adjacent tissues (NATs) in the Liu’s cohort^26^ (Supplementary Fig. 4j, k). Corresponding to the stages in ESCC progression, *TP53* mutation displayed positive impacts on DNA replication/cell cycle in the IEN phase, and ECM signaling in the A-ESCC phase (Supplementary Fig. 4l).

Among the top ten mutations in ESCC progression, the mutation-protein correlation analysis revealed that *MACF1* mutation upregulated its counterpart protein level (*t*-test, FDR = 5.5E–5) (Fig. 3f and Supplementary Fig. 4m). In addition, gradually increased expression of MACF1 was detected (Kruskal–Wallis test, FDR = 6.3E–9, A-ESCC vs. NT ratio = 1.5E + 3) at the protein level in ESCC progression (Supplementary Fig. 4n).

Generally, MACF1 is a multidomain protein that associates with microfilaments and microtubules, and binds to a complex (e.g., CTNND1, GSK3B, etc.) in Wnt signaling^38^. In our study, significant correlations were found between MACF1 and GSK3A (Pearson’s *R* = 0.46, *p* = 3.0E–4), and MACF1 and GSK3B (Pearson’s *R* = 0.28, *p* = 0.027) at the protein level (Fig. 3g and Supplementary Fig. 4o). In addition, consistent results were found in TCGA cohort^15^ at the RNA level (Supplementary Fig. 4p), inferring the mutation of *MACF1* would activate Wnt signaling. To investigate the impacts, we integrated the MACF1 positively associated proteins in our cohort and TCGA cohort, and found that those proteins participated in Wnt signaling (Fig. 3h). Of note, the incorporation of the proteins in Wnt signaling affected by *MACF1* mutation, were highly expressed in the A-ESCC phase (Kruskal–Wallis test, FDR < 0.05, A-ESCC vs. NT ratio ≥ 2), such as GSK3A, GSK3B, CSNK1A, CSNK2B, DVL3, etc. (Fig. 3i). Furthermore, the CRISPR-mediated depletion of the genes (e.g., CSNK2B, CSNK1A, etc.) reduced the proliferation of ESCC cell lines (Fig. 3i). At the phosphoprotein level, the overrepresented phosphorylation of CTNNB1 (T551, S191) and CTNND1 (S346, Y865), were observed in the A-ESCC phase (Kruskal–Wallis test, FDR < 0.05, A-ESCC vs. NT ratio ≥ 2) (Supplementary Fig. 4q). Therefore, the mutation of *MACF1* upregulated the protein level of MACF1, which bound to the Wnt complex and activated Wnt signaling in the A-ESCC phase (Fig. 3j). Collectively, we uncovered that *TP53* mutation observed in the whole ESCC stages exhibited diverse impacts during ESCC carcinogenesis, and *MACF1* mutation had roles in promoting ESCC progression in the A-ESCC phase.

### Proteomic characterization of three phases in ESCC progression

During the carcinogenesis of ESCC, three phases covered the NT phase, the IEN phase, and the A-ESCC phase. Specifically, the NT phase contained stage 1 and stage 2, the IEN phase covered stage 3 last till to stage 7, and the A-ESCC phase included the advanced stages T2 and T3 (Supplementary Fig. 5a). To explore the characteristics of the three phases of ESCC, we analyzed the TMB and differentially expressed proteins (DEPs) of the three phases. As a result, the comparative analysis revealed that the NT phase, with the lowest TMB, was featured by metabolic process (e.g., KLK12/13, ALOX12, etc.) and inflammatory response (e.g., IL36A, SERPINB1, etc.) (Supplementary Figs. 2c and 5c). DNA repair signature was detected in the IEN phase, indicating DNA damage was initiated. To react to the external damage, the proteins involved in the inflammatory response (e.g., IL36A, SERPINB1, etc.) were highly expressed in the NT phase. As shown in Figs. 2 and 3, the IEN phase of ESCC had higher TMB compared to the NT phase, and was featured with the chr3q gain. The IEN overrepresented proteins participated in the oncogenic-related pathways, such as cell cycle (e.g., CDK1/2, RB1, etc.), DNA repair (e.g., LIG1, PARP1, etc.), EGFR signaling pathway (e.g., EGFR, SOS1, etc.), and so on. Comparatively, the A-ESCC phase of ESCC, with the highest TMB, was characterized by the dominant pathways of Wnt signaling (e.g., WNT2B, GSK3A, etc.) and glycolysis (e.g., PGK1, ENO3, etc.).

To further demonstrate the findings, we analyzed the DEPs (Wilcoxon rank-signed test, FDR < 0.05, IEN vs. NT ratio ≥ 2 or ≤ 0.5) of the phases in the main cohort (*n* = 786) and the validation cohort (*n* = 256), and found consistent findings of the characterizations of the phases both in the main cohort and validation cohort. Specifically, the primary functions of esophagus were dominant in the NT phase, such as metabolic processes including keratinization (e.g., KLK12/13, CSTA, etc.), lipid and amino acid metabolism (e.g., CYP4F22, ALOX12, etc.) (Supplementary Fig. 5b–d). In addition, the overrepresented proteins in the IEN phase participated in the oncogenic-related pathways, such as cell cycle (e.g., CDK1/2, RB1, etc.), DNA repair (e.g., LIG1, PARP1, etc.), EGFR signaling pathway (e.g., EGFR, SOS1, etc.), etc. (Supplementary Fig. 5b–d). These consistent findings in the main cohort and validation cohort indicated the benign of the NT phase, and the relative malignancy of the IEN phase, highlighting the importance of investigating the key molecular events during the carcinogenesis of esophagus.

### A carcinogenesis path with eight dynamic waves in ESCC progression

**Fig. 4:**
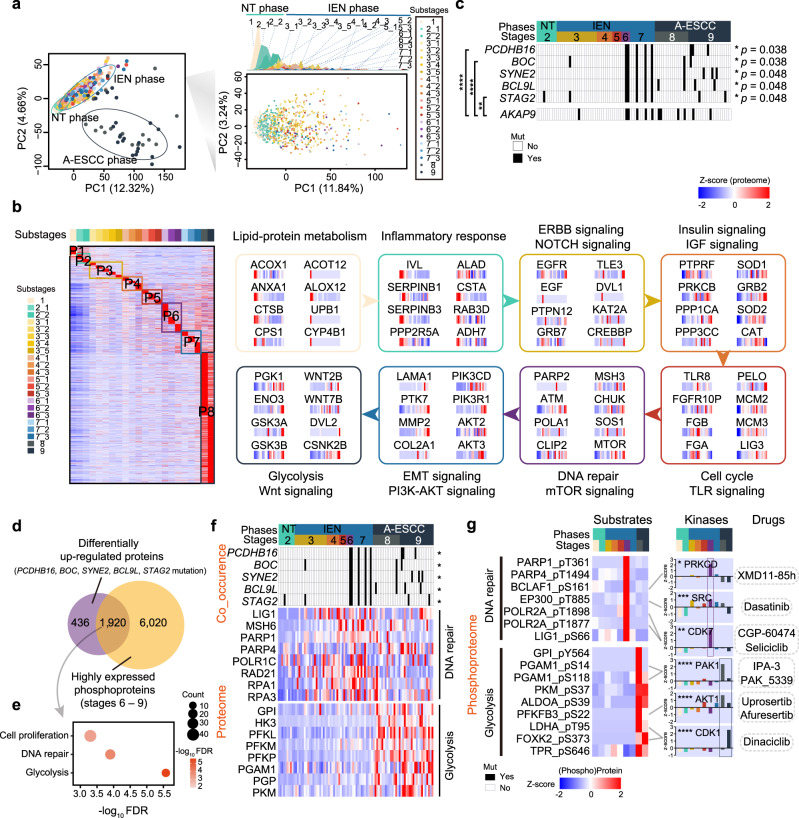
The temporal driver pathway waves in ESCC progression. **a** Principal component analysis (PCA) of the Fudan cohort. Left: PCA of all 786 ESCC samples (including NT phase, IEN phase, and A-ESCC phase); Right: PCA of 746 early ESCC samples (NT phase and IEN phase). **b** Heatmap analysis of the dynamic switches during the carcinogenesis of ESCC (Kruskal–Wallis test). Left: heatmap analysis of DEPs of the 22 substages in ESCC progression. Right: the driver pathway waves of 8 panels in ESCC progression. **c** The mutations are significantly associated with stages in ESCC progression (two-sided Fisher’s exact test). The highlighted mutations (right) are exclusively co-mutations (two-sided Fisher’s exact test). The square directs to a subset of patient samples used for WES (*n* = 102). *p* values of the co-mutations with *AKAP9*: ****p = 3.7E–5 (*PCDHB16*), ****p = 3.7E–5 (*BOC*), **p = 1.7E–3 (*STAG2*). **d** The number of the proteins regulated by the co-mutations and **e** the associated biological pathways. **f** Heatmap showing the impacts of the co-mutations of *PCDHB16*, *BOC*, *SYNE2*, *BCL9L*, and *STAG2* (top, two-sided Fisher’s exact test), on the protein level (bottom) in ESCC progression (Kruskal–Wallis test). *p = 0.038 (*PCDHB16*), *p = 0.038 (*BOC*), *p = 0.048 (*SYNE2*), *p = 0.048 (*BCL9L*), *p = 0.048 (*STAG2*). The square directs to a subset of patient samples used for WES (*n* = 102). **g** The kinase-substrate interactions in ESCC progression (Kruskal–Wallis test). A total of 145 samples for phosphoproteomic profiling are used in this analysis. *p = 0.015 (PRKCD), ***p = 2.4E–4 (SRC), **p = 9.6E–3 (CDK7), ****p = 8.0E–6 (PAK1), ****p = 5.3E–9 (AKT1), ****p = 2.8E–10 (CDK1). ****p < 1.0E–4, ***p < 1.0E–3, **p < 0.01, *p < 0.05, ns. > 0.05. Source data are provided as a Source data file.

The proteogenomics on three phases indicated a temporal correlation between genomic aberrations and proteomic alterations in ESCC progression. To further portray molecular profiles of ESCC in a time-resolved mode, we split the whole ESCC progression into 22 substages. Visualization of the abundance of the E2 proteins (*n* = 6885; Methods) by principal component analysis (PCA) (Methods) differentiated the proteome profiles for the 22 substages, which clearly discriminated the proteomes of the early and the advanced ESCC stages. In addition, PCA of early ESCCs displayed obvious diversity among the substages along with ESCC progression (Fig. 4a). These results further showed the distinct profiles of the substages in ESCC progression.

To investigate specific characteristics of the substages in ESCC progression, we performed the substage-based supervised clustering analysis (Methods) with substage highly expressed proteins (Kruskal–Wallis test, FDR < 0.05, certain substage vs. other substages ratio ≥ 2) (Fig. 4b). Based on the enrichment analysis of the substages highly expressed proteins showing that the molecular characterization of the substages in ESCC progression was associated with the lesion invasion layers, and could be grouped into eight models (1–2_1/2_2–3_1/3_2/3_3/3_4/3_5–4_1/4_2/4_3/–5_1/5_2/5_3–6_1/6_2/6_3–7_1/7_2/7_3–8/9) (Supplementary Fig. 5g). Thus, we speculated that the carcinogenesis path in ESCC progression could be linked to those eight models.

To further demonstrate the speculation, we performed co-expression analysis of the substage highly expressed proteins and identified eight protein patterns, which were consistent with the results in those eight models correlated to the lesion invasion layers (Supplementary Fig. 5h, k). In addition, the consistent findings of the enrichment pathways were also detected in eight models based on substages and eight protein patterns based on the results of co-expression analysis. Therefore, eight panels were identified on the basis of the histopathological stages in a time-resolved mode (Fig. 4b and Supplementary Fig. 5h). Specifically, in the (sub)stage 1 (normal tissue), the primary metabolic machinery was dominant, such as lipid metabolism (e.g., HMGCS1, ALOX15B, etc.), pyruvate metabolism (e.g., CYP4F12, ACOX3, etc.), and amino acid metabolism (e.g., ANXA1, CTSB, etc.). In stage 2 (hyperplasia stage), the expression of proteins, including PPP2R5A, SERPINB3, and CSTA, involved in the immune response to external damage, were overrepresented. Enhancement of ERBB signaling and NOTCH signaling, and the related proteins (e.g., EGFR, GRB7, etc.) were detected in stage 3 (Tis stage). In stage 4 (lamina propria stage), insulin signaling and IGF signaling-related proteins were overrepresented (e.g., SOD1/2, PPP3CC, etc.). Cell cycle/TLR signaling (e.g., MCM2/3, TLR8, etc.) and DNA repair/mTOR signaling (e.g., MSH3, MTOR, etc.) were the dominant pathways in stage 5 (muscularis mucosa stage), and the stage 6 (sm a stage), respectively. In stage 7 (sm b stage), PI3K-AKT signaling and EMT signaling (e.g., AKT2/3, COL2A1, etc.) were dominant, of which EMT signaling is involved in tumor-initiation and motility^39^. Glycolysis and Wnt signaling (e.g., PGK1, WNT2B/7B, etc.) related proteins were overrepresented in stages 8 (T2 stage) and 9 (T3 stage). PGK1, an important glycolytic enzyme, though not mutated at the gene level, was highly increased at the protein level, especially in the T2 and T3 stages of ESCC, which provided further evidence for the crucial role of glycolysis, and suggested the potential function of PGK1 in ESCC progression.

In the validation cohort, 256 samples covered 6 of 9 histopathological stages, in which stages 6 (submucosal invasion cancer stage a), 8 (T2), and 9 (T3), were not included. Substage-based supervised clustering analysis identified six proteomic patterns (V1–V6), covering six of eight patterns in the main cohort (Supplementary Fig. 5i). In addition, the stages in the proteomic panels were consistent with those in the main cohort.

To further demonstrate the result at the molecular level, we analyzed the DEPs of the stages (Kruskal–Wallis test, FDR < 0.05), and found that the molecular characterizations of the six patterns in the validation cohort were consistent with those in the main cohort (Supplementary Fig. 5j). Specifically, the V1 (stage 1, normal tissues) proteins were associated with lipid and protein metabolism (e.g., ACOX1, ALOX12, etc.) (Supplementary Fig. 5j, k). In the V2 (stage 2, hyperplasia stage), inflammatory response (e.g., COL6A5, SERPINB3, etc.) was dominant. NOTCH signaling (e.g., ADAM10, CREBBP, etc.) and insulin signaling (e.g., IRS2, PPP1CA, etc.) were the dominant pathways in the V3 (stage 3, Tis stage) and V4 (stage 4, lamina propria stage), respectively. At the muscularis mucosa stage (stage 5, V5), cell cycle and TLR signaling (e.g., MCM2, TLR2, etc.) were dominant. The V6 (stage 7, submucosal invasion cancer stage b) proteins participated in EMT signaling and PI3K-AKT-mTOR signaling (e.g., MMP1/2, PIK3CB, etc.) were consistent with the molecular features of the submucosal invasion cancer stage in the main cohort. Therefore, the molecular characterizations of the proteomic patterns in the validation cohort were consistent with those in the main cohort (Supplementary Fig. 5k).

At the gene level, we observed the stage-associated mutations including *PCDHB16* (Fisher’s exact test, *p* = 0.038), *BOC* (Fisher’s exact test, *p* = 0.038), *SYNE2* (Fisher’s exact test, *p* = 0.048), *BCL9L* (Fisher’s exact test, *p* = 0.048), *STAG2* (Fisher’s exact test, *p* = 0.048), which were detected in stage 6 and last till to the stage 9 (Fig. 4c). In addition, the mutations of *PCDHB16*, *BOC*, and *STAG2* were co-occurrent with *AKAP9* mutation, which exhibited positive impacts on glycolysis. The recurrent co-occurrence of genomic events helps to dissect the genomic complexity underlying tumor progression^40^, thus enabling us to explore the functional impacts of the co-occurrence mutations in ESCC progression. Furthermore, the multi-dimensional omics data provided an excellent chance to explore the relationships between the genome and the proteome in the time-resolved ESCC progression. We thus performed enrichment pathway analysis using the DEPs (*n* = 608), which were the overlapped between the differentially upregulated proteins (*n* = 1748, Mut vs. WT ratio ≥ 2) and highly expressed phosphoproteins (*n* = 2818, submucosa stage to T3 stage) (Fig. 4d). The results showed that those proteins participated in DNA repair, cell proliferation, and glycolysis (Fig. 4e). Specifically, DNA repair (e.g., LIG1 S66, PARP1 T361, etc.) and glycolysis (e.g., GPI Y564, PGAM1 S118, etc.) related proteins were overrepresented in the submucosa stages (stages 6 and 7) and in the advanced stages (stages 8 and 9) of ESCC, respectively, at the protein and phosphoprotein levels (Kruskal–Wallis test, FDR < 0.05, certain stage vs. other stages ratio ≥ 2) (Fig. 4f, g).

To explore the kinase-substrate interactions in ESCC progression, we integrated the overrepresented kinases in the co-occurrence mutations group, and performed the kinase-substrate analysis based on phosphoprotein data. As a result, we found the kinases-substrate regulation was notably consistent with the dynamic waves in ESCC progression. Specifically, the stage 6 overrepresented kinases (e.g., PRKCD, SRC, CDK7) (Kruskal–Wallis test, FDR < 0.05, stage 6 vs. other stages ratio ≥ 2), showed positive regulation with the DNA repair-related phosphoproteins, such as BCLAF1 S161, EP300 T885, POLR2A T1898, etc. The T2 and T3 stages overrepresented kinases (e.g., PAK1, AKT1, CDK1) (Kruskal–Wallis test, FDR < 0.05, T2 and T3 stages vs. other stages ratio ≥ 2), displayed positive regulation on glycolysis related phosphoproteins, evidenced by the overrepresented phosphorylation of PGAM1 S14 and S118, PFKFB3 S22, LDHA T95, etc. (Fig. 4g). These results suggested the potential therapy of kinase inhibitor (e.g., XMD11-85h, Dasatinib, CGP-60474/Seliciclib, etc.) in ESCC progression.

Taken together, a carcinogenesis path with eight dynamic waves in ESCC progression was revealed on the basis of the consistency among the genomic aberrations, proteomic alterations, and phosphoproteomic actions: metabolism (e.g., ANXA1 and CTSB) – DNA damage (e.g., PPP2R5A and SERPINB3) – cell proliferation (e.g., EGFR and EGF) – lesion invasion (e.g., GRB2 and PRKCB) – cell cycle (e.g., MCM2/3 and FGFR1OP) – cell differentiation (e.g., MSH3 and MTOR) – tumor metastasis (e.g., MMP2 and AKT2/3) – esophageal carcinogenesis (e.g., PGK1 and CTNNB1) (Supplementary Fig. 5k). Furthermore, these results also defined the substages-specific molecular characteristics and uncovered the potential candidates for ESCC malignancy.

### Proteome clusters of ESCC progression

**Fig. 5:**
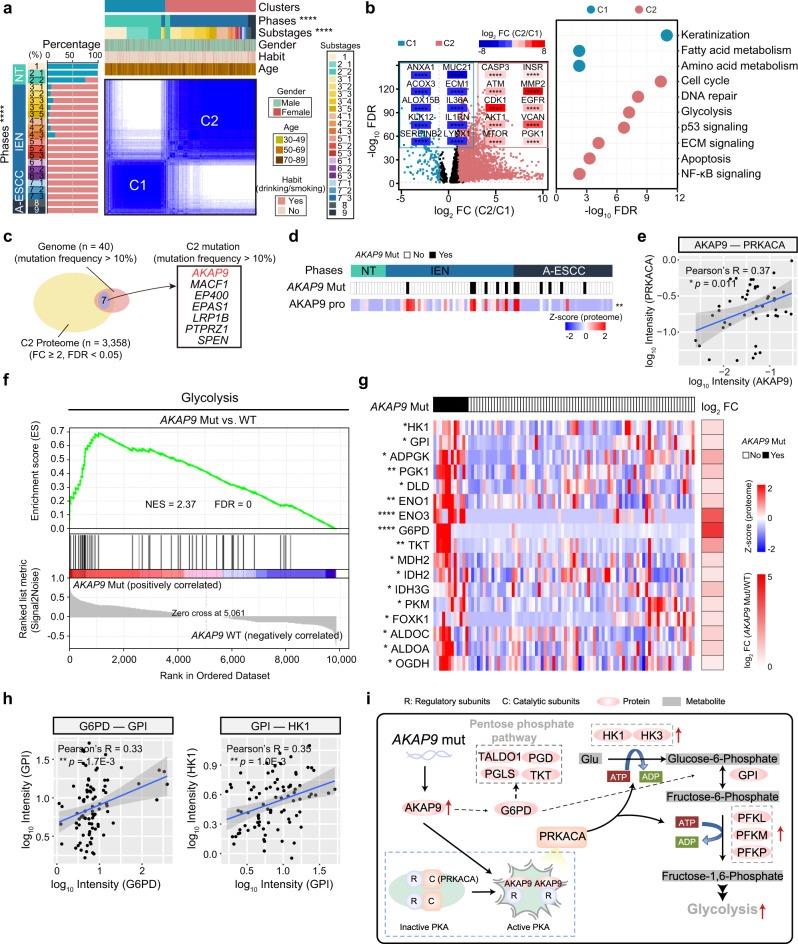
Proteomic clusters and the impacts of *AKAP9* mutation in ESCC progression. **a** Consensus clustering analysis of 786 samples (two-sided Fisher’s exact test). Left: the percentages of the two clusters in 22 substages; Right: 786 samples were classified into two clusters based on proteomic patterns. *p = 0.029 (Age), ****p < 2.2E–16 (Phases), ****p < 2.2E–16 (Substages). **b** Volcano analysis of DEPs (left) in the two clusters and their associated biological pathways (right) in the two clusters (two-sided Student’s *t*-test). Biological pathways were analyzed from the Reactome database. C1: the Cluster 1. C2: Cluster 2. **c** Venn diagram depicting the number of the genes both detected in the genome and proteome in C2. The right shows the significant C2 mutations with mutation frequency over 10%. **d** Heatmap showing the impacts of *AKAP9* mutation on the protein level of AKAP9 (two-sided Student’s *t*-test, BH-adjusted **p = 8.4E–3). **e** Scatterplot showing the relationship between log_10_ PRKACA and log_10_ AKAP9 expression at the protein level (two-sided Pearson’s correlation test, mean ± SD). **f** GSEA plot (KEGG gene sets) for glycolysis in *AKAP9* mutation and WT comparison. **g** Heatmap depicting the impacts of *AKAP9* mutation on glycolysis in ESCC progression (two-sided Student’s *t*-test, BH-adjusted *p < 0.05). The square directs to a subset of patient samples used for WES (*n* = 102). **h** Scatterplots showing the relationship between log_10_ G6PD (left)/HK1 (right) and log_10_ GPI expression at the protein level (two-sided Pearson’s correlation test, mean ± SD). **i** A brief summary of the impacts of *AKAP9* mutation. ****p < 1.0E–4, ***p < 1.0E–3, **p < 0.01, *p < 0.05, ns. > 0.05. Source data are provided as a Source data file.

Consensus clustering (Methods) identified two major proteomic clusters which showed association with the classification of the (sub)stages in ESCC progression: Cluster 1 (C1, *n* = 314) included the stage 1, and 2, and Cluster 2 (C2, *n* = 472) contained the rest of the stages, including the T2 and T3 stages (Fig. 5a, Supplementary Fig. 6a, and Supplementary Data 5a). Comparative analysis of the later-stage samples (especially the IEN phase samples) of C1 and C2 with the clinic features disclosed that the later samples in the C1 were prominent in the younger ESCC patients (<50 years old, Fisher’s exact test, *p* = 0.029, 15.0%) (Supplementary Fig. 6b), indicating the potential impacts of ages in ESCC progression. In addition, the two clusters were associated with 3 phases in ESCC progression, in which the C2 contained the most samples in the IEN phase (95.3%) and the A-ESCC phase (100%), reflecting more malignancy of the C2 (Supplementary Fig. 6c). This distinct two-stage separation suggested that the irreversible fundamental proteome alterations were detected as early as in the Tis stage (stage 3). Van der Schaaf et al. pointed out that the Tis stage was associated with worse survival^41^, further indicating the malignancy of Tis stage in ESCC progression.

The significance analysis of microarray (SAM)^42^ analysis (Methods) was performed to investigate the characteristics of the two clusters at the protein level, which identified 2922 DEPs between C1 and C2 (*t*-test, FDR < 0.05, C2 vs. C1 ratio ≥ 2 or ≤ 0.5), including 168 and 2754 proteins overrepresented in the C1 and C2, respectively (Supplementary Fig. 6d and Supplementary Data 5b). The results showed that the oncogenic pathways-related proteins, including ATM, CDK1, EGFR, CASP3, VCAN, etc., were significantly overrepresented in the C2. On the contrary, the overrepresented proteins of the C1, including KLK12, ACOX3, ALOX15B, and ANXA1, involved in keratinization and inflammatory response, were associated with the primary biological function of normal esophagus (Fig. 5b and Supplementary Fig. 6g). To validate the molecular characterization of two proteomic clusters, we performed PCA of the 256 samples in the validation cohort, which were also classified into two clusters. Consistent with the findings in the main cohort, the C1 included stages 1 and 2, and the C2 contained the rest of the stages in the validation cohort (Supplementary Fig. 6e). In addition, the proteomic clusters in the validation cohort also showed a positive association with the phases in ESCC progression, and specifically, the C1 (Fisher’s exact test, *p* < 1.0E–4, 89.5%) and C2 (Fisher’s exact test, *p* < 1.0E–4, 64.4%) consisted most of the samples in the NT phases and the IEN phase, respectively. Furthermore, based on the SAM analysis, the C1 proteins in the validation cohort participated in the primary biological function of normal esophagus, such as keratinization (e.g., KLK12/13, CSTA, etc.), fatty acid metabolism (e.g., ACOX3, ALOX12, etc.), and amino acid metabolism (e.g., MGLL, CYP4F12, etc.) (Supplementary Fig. 6f, g). The oncogenic pathways-related proteins were overrepresented in the C2 both in the main cohort and validation cohort, including cell cycle (e.g., CDK1, SKP1, etc.), DNA repair (e.g., DDB1, XPC, etc.), glycolysis (e.g., ENO2, HK3, etc.), and so on. The specific biomarkers of normal esophagus annotated from Human Proteome Atlas (HPA, https://www.proteinatlas.org), including GBP6, TGM1, TGM3, and S100A14, were overrepresented in the C1, which gradually decreased from the normal stage (stage 1) to advanced stages (stages 8 and 9) of ESCC (Kruskal–Wallis test, FDR < 2.2E–16, stage 9 vs. stage 1 ratio ≥ 2) (Supplementary Fig. 6h, i). Taken together, these results further confirmed the dysregulation of metabolism and oncogenic pathways in ESCC progression.

### The *AKAP9* mutation enhanced glycolysis in the A-ESCC phase

Incorporation of genomic aberrations (*n* = 40, mutation frequency > 10%) and the C2 overrepresented proteome (*n* = 3385, C2 vs. C1 ratio ≥ 2) revealed that seven mutations (e.g., *AKAP9*, *MACF1*, *EP400*, etc.) were exclusively detected in the C2 with mutation frequency over 10% (Fig. 5c). Furthermore, the mutation of *AKAP9* was associated with poor prognosis outcomes of ESCC patients (log-rank test, *p* = 0.029) indicating the potential functions of *AKAP9* mutation in ESCC progression (Supplementary Fig. 6j).

AKAP9, one of the A-kinase anchoring proteins (AKAPs), binds to the regulatory subunit of AMP-dependent protein kinase (PKA) and achieves the activation of PKA^43^, which regulates multiple signaling cascade^44^, such as glucose metabolism including glycolysis^45^. In our cohort, we found the mutation of *AKAP9* upregulated the protein level of AKAP9 (*t*-test, *AKAP9* Mut vs. WT ratio = 6.58, FDR = 8.4E–3) (Fig. 5d and Supplementary Fig. 6k). Moreover, AKAP9 showed a significantly positive correlation with PRKACA, one of the PKA catalytic subunits^46^, at the protein level (Pearson’s *R* = 0.37, *p* = 0.011) (Fig. 5e). Gene set enrichment analysis (GSEA) demonstrated that the *AKAP9* mutation positive-correlated proteins were converged on glycolysis (normalized enrichment score (NES) = 2.37, FDR = 0) (e.g., HK1, GPI, PGK1, etc.), which were gradually increased in ESCC progression (Kruskal–Wallis test, FDR < 0.05, A-ESCC vs. NT ratio ≥ 2) (Fig. 5f, g). Consistently, the expression of AKAP9 was significantly overrepresented in the C2 in the validation cohort (*t*-test, FDR = 3.1E–3, C2 vs. C1 ratio = 3.29) (Supplementary Fig. 6l). We integrated AKAP9 positively associated proteins (*n* = 1344), and found those proteins were involved in glucose metabolism, such as glycolysis, TCA cycle, etc. (Supplementary Fig. 6m). These consistent findings further indicated the impacts of *AKAP9* mutation on glycolysis in ESCC progression.

Allosteric regulation refers to the process where the effect of binding of a ligand at one site of a protein is transmitted to another, often distant, functional site, and thus regulates biological processes including glucose metabolism^47^. As well as HK1 and GPI, G6PD, as one of the activators in the transformation process from glucose-6-phosphate to fructose-6-phosphate^48^, showed a significant association with GPI (Pearson’s *R* = 0.33, *p* = 1.7E–3), further demonstrating the activation of glycolysis (Fig. 5h and Supplementary Fig. 6n). At the phosphoprotein level, we also detected the increased phosphorylation of glycolysis (e.g., ENO1 S272, PGAM1 S189, etc.) in ESCC progression (Kruskal–Wallis test, FDR < 0.05, A-ESCC vs. NT ratio ≥ 2) (Supplementary Fig. 6o). Taken together, the mutation of *AKAP9*, activated PKA and enhanced the energy formation, elevating the process of glucose metabolism, especially glycolysis, in the A-ESCC phase (Fig. 5i). Thus, our study provided valuable insights into the proteomic characteristics with *AKAP9* mutation and revealed the impacts on glycolysis in ESCC progression at the multi-omics level.

### Personalized trajectory revealed six major carcinogenesis tracks of early ESCC

**Fig. 6:**
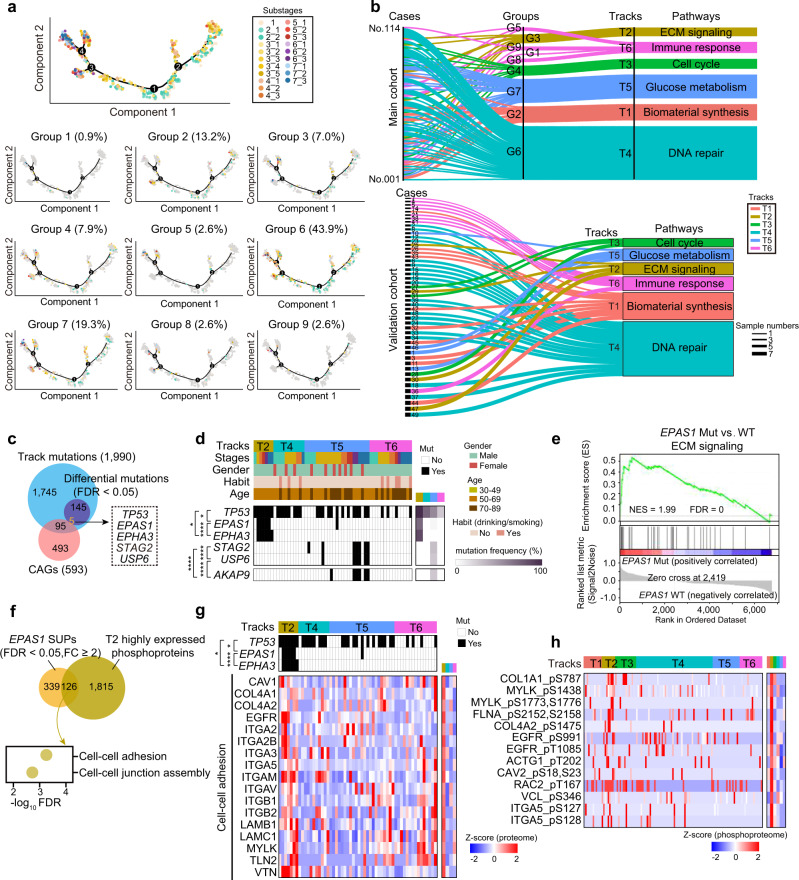
Personalized trajectory reveals six major carcinogenesis tracks of the early ESCCs. **a** The trajectory of 746 samples (top) and 114 early ESCC cases were grouped into 9 (bottom). **b** Sankey diagram analysis of 114 early ESCC cases (top, main cohort) and 49 early ESCC cases (bottom, validation cohort). **c** Venn diagram showing the track mutations (top) and the CAGs (bottom) (two-sided Fisher’s exact test). CAGs: cancer-associated genes. The overlapped mutations are shown in the box. **d** The CAG-associated track mutations in the early ESCCs. The co-mutations are highlighted on the left (two-sided Fisher’s exact test), and the mutation frequency is shown on the right. The square directed to a subset of patient samples used for WES (*n* = 68) in early ESCCs. Co-mutations: *p = 0.032 (*TP53* and *EPAS1*), *p = 0.032 (*TP53* and *EPHA3*), ****p = 9.3E–6 (*EPAS1* and *EPHA3*), *p = 2.2E–7 (*STAG2* and *USP6*), ****p = 9.3E–6 (*USP6* and *AKAP9*), ****p = 3.2E–5 (*STAG2* and *AKAP9*). **e** GSEA plot (KEGG gene sets) for ECM signaling in *EPAS1* mutation and WT comparison. **f** Venn diagram depicting the number of the overlapped proteins enhanced by the mutation of *EPAS1* and T2 enhanced phosphoprotein (top), and the associated biological pathways (bottom). SUPs: the significantly upregulated proteins. **g** Heatmap showing the represented protein in the cell–cell adhesion positive associated with *EPAS1* mutation (two-sided Fisher’s exact test). The square directs to a subset of patient samples used for WES (*n* = 68) in early ESCCs. Co-mutations: *p = 0.032 (*TP53* and *EPAS1*), *p = 0.032 (*TP53* and *EPHA3*), ****p = 9.3E–6 (*EPAS1* and *EPHA3*). **h** Heatmap showing the phosphorylation of the phosphoproteins in cell–cell adhesion (Kruskal–Wallis test). The square directs to a subset of patient samples used for phosphoproteome (*n* = 119) in early ESCCs. ****p < 1.0E–4, ***p < 1.0E–3, **p < 0.01, *p < 0.05, ns. > 0.05. Source data are provided as a Source data file.

The diversity and tumoral heterogeneity of ESCC remain challenging to decide precise clinical strategies for different ESCC patients who have diverse featured carcinogenesis tracks. To this end, we used trajectory inference methods^49^ (Methods) to trace the carcinogenesis lineages of early ESCC in the cohort. As a result, six major tracks were classified (13.2%, 7.0%, 7.9%, 43.9%, 19.3%, and 8.7% of patients, respectively) (Fig. 6a, b and Supplementary Data 6a). The track proteins were determined by the expression trend along with ESCC progression (Kruskal–Wallis test, FDR < 0.05, stage 9 vs. stage 1 ratio ≥ 2), and the dominant pathways of six major tracks were annotated as follows: (1) track 1 (T1, *n* = 15), biomaterial synthesis; (2) track 2 (T2, *n* = 8, non-drinking/smoking track), ECM signaling; (3) track 3 (T3, *n* = 9, female track), cell cycle; (4) track 4 (T4, *n* = 50, mainstream track (mainstream population of ESCC patients)), DNA repair; (5) track 5 (T5, *n* = 22, older track), glucose metabolism; and (6) track 6 (T6, *n* = 10, drinking/smoking track), immune response (Fig. 6b and Supplementary Data 6b, c). The proteins, of which the expression was gradually decreased in six tracks (Kruskal–Wallis test, FDR < 0.05, stage 9 vs. stage 1 ratio ≤ 0.5), were involved in the primary biology of normal esophagus, such as epithelial cell differentiation (e.g., EVPL and AHNAK2) and keratinization (e.g., FLG, KLK12/13 and SPRP3) (Supplementary Fig. 7a).

Furthermore, different tracks were closely associated with various clinical features of early-stage ESCC patients, which improved our understanding of tumor heterogeneity. For example, track 3, featured with cell cycle, had the highest proportion of female patients (Fisher’s exact test, *p* = 7.7E–14, 29.2%) (Supplementary Fig. 7c), significantly greater than the natural proportion of female patients in ESCC (no more than 10%)^50^. Track 6, featured as immune response, had the highest proportion of patients with drinking/smoking habits (Fisher’s exact test, *p* = 2.3E–16, 18.1%), which was associated with chronic inflammatory and regulated oxidative stress in various cancer types^51^, revealing the unique track in the patients with drinking/smoking habits.

To validate the features of the six major tracks, the trajectory inference methods were also applied to 49 early-stage ESCC patients in the validation cohort. As a result, we found all the patients in the validation cohort were also classified into tracks 1–6, of which the molecular characterization was similar to those in track 1 to track 6 from 114 early-stage ESCC patients in the main cohort (Fig. 6b, Supplementary Fig. 7b, d, and Supplementary Data 6d–f). Specifically, the ESCC patients with drinking/smoking habits were prominent in track 6 of the validation cohort (Fisher’s exact test, *p* = 5.1E–9, 18.6%) (Supplementary Fig. 7c). To explore the features of the ESCC patients with drinking/smoking habits, we integrated the gradually increased proteins, which participated in the immune response pathways, including interleukins signaling, antigen processing and presentation, etc. (Supplementary Fig. 7e). The consistent findings in the characterizations of track 6 were observed in the main and validation cohorts, further validating the findings of the personalized tracks.

At the gene level, we identified 150 differential track mutations, 5 of which were also covered in CAGs with mutation frequency over 10%, including *TP53*, *EPAS1*, *EPHA3*, *STAG2*, and *USP6* (Fig. 6c and Supplementary Data 6g). Furthermore, we found the mutations of *STAG2* (Fisher’s exact test, *p* = 1.1E–3, track 2 vs. track 4 vs. track 5 vs track 6 = 0% vs. 0% vs. 30.4% vs. 0%) and *USP6* (Fisher’s exact test, *p* = 3.1E–3, track 2 vs. track 4 vs. track 5 vs track 6 = 0% vs. 0% vs. 26.1% vs. 0%) were prominent in track 5, which were all co-occurrence with *AKAP9* mutation (Fisher’s exact test, *p* = 3.2E–5 for *STAG2* and 9.3E–6 for *USP6*), suggesting the roles of glycolysis in the ESCC patients in track 5 (Fig. 6d). In addition, GSEA showed the positive impacts of the mutations of *STAG2* (NES = 2.10, FDR = 7.2E–4), and *USP6* (NES = 2.04, FDR = 0) on pentose phosphate pathway (Supplementary Fig. 7f), which were the evidence of glucose metabolism characteristics in track 5.

Notably, the mutations of *TP53* (Fisher’s exact test, *p* = 7.2E–3, track 2 vs. track 4 vs. track 5 vs track 6 = 100% vs. 73% vs. 57% vs. 27%), *EPAS1* (Fisher’s exact test, *p* = 3.2E–5, track 2 vs. track 4 vs. track 5 vs track 6 = 71% vs. 0% vs. 4% vs. 0%), and *EPHA3* (Fisher’s exact test, *p* = 2.2E–7, track 2 vs. track 4 vs. track 5 vs track 6 = 86% vs. 0% vs. 0% vs. 0%) were prominent in track 2. As shown in Supplementary Fig. 4l, *TP53* mutation displayed positive impacts on ECM signaling, demonstrating the characteristics of track 2. GSEA displayed the positive impacts of the mutations of *EPAS1* (NES = 1.99, FDR = 0), *EPHA3* (NES = 1.83, FDR = 1.6E–3) on ECM signaling (Fig. 6e and Supplementary Fig. 7g). Furthermore, *EPAS1* mutation was co-occurrence with the mutations of *TP53* (Fisher’s exact test, *p* = 0.032) and *EPHA3* (Fisher’s exact test, *p* = 9.3E–6). To elucidate the impacts of *EPAS1* genomic aberrations on proteomic alterations and phosphoproteomic actions, we integrated the molecules (*n* = 126) overlapped in the *EPAS1* mutation SUPs and track 2 overrepresented phosphoproteins. As a result, we found those overlapped molecules were involved in cell–cell adhesion (e.g., ITGA5, EGFR, etc.) (Fig. 6f, g). Compared with other tracks, the phosphorylation of the cell–cell adhesion proteins (ITGA5 S127 and S128, EGFR S991, etc.) was overrepresented in track 2 (Fig. 6h). Together, our study revealed six major carcinogenesis tracks, and found the track specific mutations had positive impacts on the track carcinogenesis lineages of ESCC.

### PGK1, aberrant glycolytic enzyme, is a potential therapeutic target

**Fig. 7:**
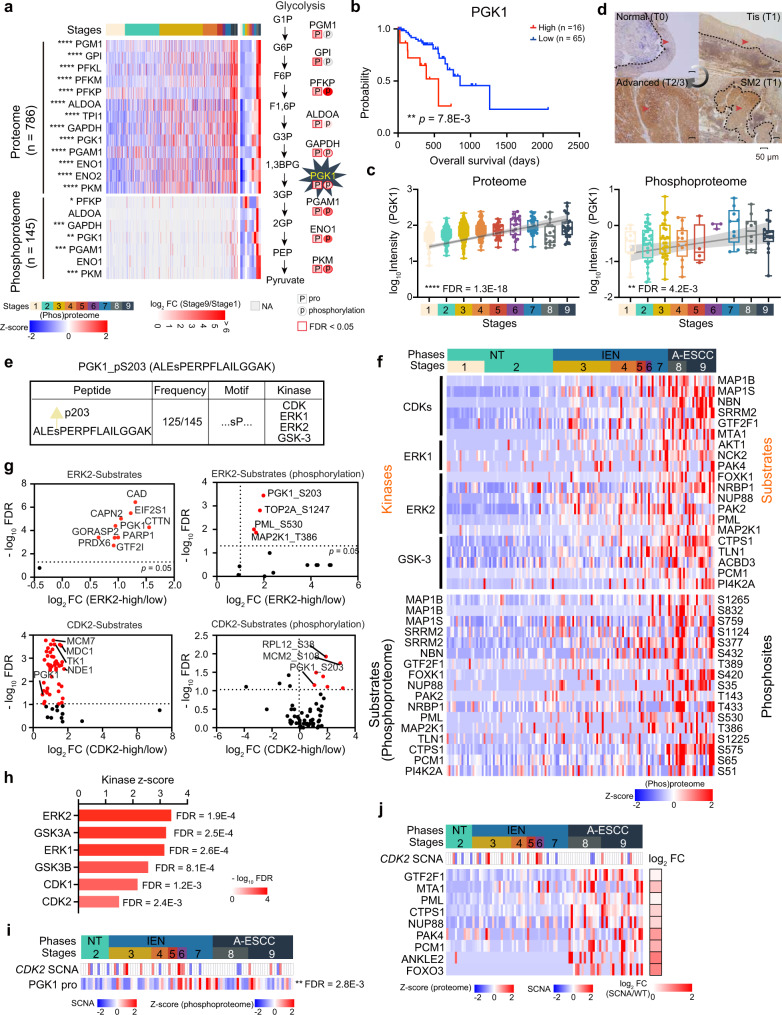
Aberrant glycolytic metabolism in ESCC and alterations in the activities of its key enzyme, PGK1. **a** Aberrant glycolysis in ESCC progression at the multi-omics level (Kruskal–Wallis test, BH-adjusted *p < 0.05). A total of 786 samples for proteomic profiling and 145 samples for phosphoproteomic profiling are used. **b** Highly expressed PGK1 is negatively correlated to prognosis (two-sided log-rank test). **c** Boxplots showing the increased expression (log_10_-transformed Intensity) of PGK1 in ESCC at the protein (left) and phosphoprotein (right) levels (Kruskal–Wallis test). Boxplots show median (central line), upper and lower quartiles (box limits), 1.5× interquartile range (whiskers). A total of 786 samples and 145 samples were used for proteome and phosphoproteome, respectively. Proteome: *n* (stage 1) = 114, *n* (stage 2) = 206, *n* (stage 3) = 259, *n* (stage 4) = 86, *n* (stage 5) = 32, *n* (stage 6) = 17, *n* (stage 7) = 32, *n* (stage 8) = 16, *n* (stage 9) = 24 biologically independent samples examined. Phosphoproteome: *n* (stage 1) = 20, *n* (stage 2) = 37, *n* (stage 3) = 31, *n* (stage 4) = 14, *n* (stage 5) = 5, *n* (stage 6) = 3, *n* (stage 7) = 9, *n* (stage 8) = 10, *n* (stage 9) = 16 biologically independent samples examined. **d** Immunohistochemistry analysis of PGK1 expression in normal (T0), Tis (T1), SM2 (T1), and advanced stage (T2/T3) tissues. The zone with the dotted lines and red arrow represents PGK1 positive staining. The scale bar indicates 50 µm. **e** Analysis of the serine motif of PGK1 (sP). The top shows the sequence and phosphorylated sites of PGK1 (S203). The bottom presents that PGK1 S203 is detected at almost samples in ESCC (125/145) and the kinases associated with the motif of PGK1 S203 (“sP”). **f** The expression of the kinases and the substrates in ESCC progression at the phosphoprotein level. The square directs to a subset of patient samples used for phosphoproteome. **g** Volcano plot displaying the ERK2-substrates (top) and CDK2-substrates (bottom) regulation results (two-sided Wilcoxon rank-sum test). The red marks the overrepresented substrates (left) and phosphorylations (right) in the kinases highly expressed group. **h** Histogram showing the *Z*-score and FDR of the KSEA results. A total of 145 samples for phosphoproteomic profiling are used in the analysis. **i** The SCNAs of *CDK2* have positive effects on PGK1 expression (two-sided Wilcoxon signed-rank test). **j** The impacts of the SCNAs of *CDK2* (middle) on the substrates expression of the kinases (bottom), associated with the PGK1 motif (sP). The square directs to a subset of patient samples used for WES (*n* = 102). ****p < 1.0E–4, ***p < 1.0E–3, **p < 0.01, *p < 0.05, ns. > 0.05. Source data are provided as a Source data file.

Abnormal glycolytic metabolism was observed in the whole process of ESCC carcinogenesis at the multi-omics level, which dramatically increased throughout carcinogenesis (Fig. 7a). PGK1, the first ATP-generating enzyme in glycolysis, gradually increased in ESCC progression in all 6 tracks at the protein level and identified as the nominated drug-targetable protein in ESCC progression (Supplementary Fig. 8a). In addition, we performed Cox regression analysis to assess the prognostic value of PGK1 expression, which was negatively correlated with the OS of ESCC in the TCGA dataset (log-rank test, *p* = 7.8E–3) (Fig. 7b and Supplementary Data 7a). To further cross-validate these results, our dataset confirmed that the expression of PGK1 was gradually increased in ESCC at the protein and phosphoprotein levels (Kruskal–Wallis test, FDR = 1.3E–18 for proteome and 4.2E–3 for phosphoproteome, stage 9 vs. stage 1 ratio = 2.30 for proteome and 2.49 for phosphoproteome) (Fig. 7c), as demonstrated by immunohistochemistry of ESCC FFPE slides in which PGK1 was gradually increased in the process from T0 stage (normal tissue) to the Tis stage, SM2 stage, and advanced stage (Fig. 7d). In addition, the elevated protein of PGK1 was identified in more than 75% (476/672) samples for proteome and 60% (75/125) for phosphoproteome from the stage 2 to stage 9 (*n* = 672 for proteome and 125 for phosphoproteome) (Fig. 7a, c). The only identified motif (sP) of PGK1 was ubiquitously (125/145) detected, and the expression of PGK1 S203 was also gradually increased with ESCC progression (Kruskal–Wallis test, FDR = 0.011, stage 9 vs. stage 1 ratio = 2.44) (Fig. 7e and Supplementary Fig. 8b).

To investigate the proteome-phosphoproteome regulation between the motif of PGK1 and the kinases, we applied motif extraction algorithm^52^ to MS phosphorylation dataset from 145 samples. The results revealed the association of ERK1/2, CDKs, and GSK-3 with the motif of PGK1 (sP)^53^, whose downstream substrates and the related corresponding phosphorylations, including MAP1S (S759), FOXK1 (S420), MAP2K1 (T386), etc., were increased in ESCC progression at the protein and phosphoprotein levels (Kruskal–Wallis test, FDR < 0.05, stage 9 vs. stage 1 ratio ≥ 2) (Fig. 7f). In addition, we found that PGK1 and the phosphorylation (S203) were overrepresented in the kinases highly expressed group (two-sided Wilcoxon rank-sum test, FDR < 0.05, highly vs. lowly expressed group ratio ≥ 2), especially in ERK2 and CDK2 highly expressed group (Fig. 7g), indicating the potential functions of ERK2 and CDK2 in the activation of PGK1 (S203) in ESCC progression.

Our previous study has found ERK1/2 could phosphorylate PGK1 S203 and result in the mitochondrial translocation of PGK1^54^. In this study, we performed the kinase-substrate enrichment analysis (KSEA) of the phosphoproteome of PGK1 (S203). The results of the score and FDR showed that ERK2 was the top-rank one kinase to activate PGK1 in ESCC progression (Fig. 7h). Furthermore, we found that the SCNAs of *CDK2* had positive impacts on the expression of PGK1 (Wilcoxon rank-sum test, FDR = 2.8E–3) (Fig. 7i), and the substrates (e.g., GTF2F1, PAK4, etc.) of kinases (Fig. 7j). These findings indicated that the SCNA of *CDK2* and ERK1/2 synergistically induced the total activity of PGK1 through increasing PGK1 expression both at the protein and phosphoprotein levels. Thus, we proposed that PGK1 S203 could be implicated in identifying the potential therapeutic target to manage ESCC.

**Fig. 8:**
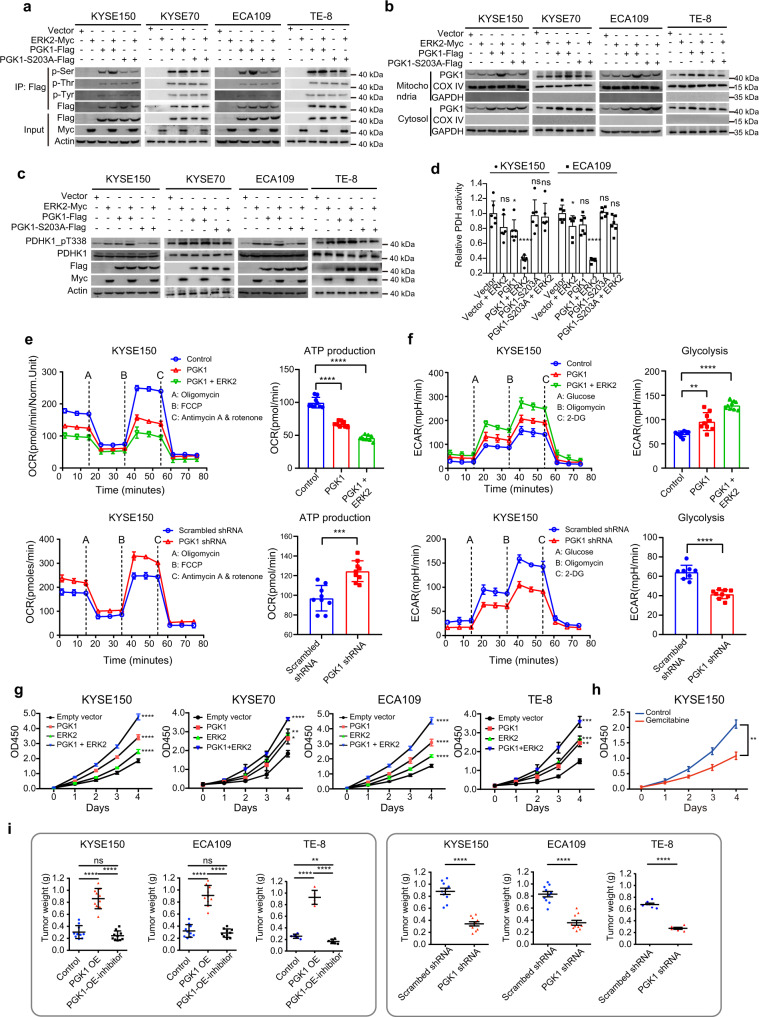
PGK1 reprograms glucose metabolism and contributes to ESCC progression. **a** Pan Serine/Threonine/Tyrosine-phosphorylation levels of PGK1 in KYSE150 cells, KYSE70 cells, ECA109 cells, and TE-8 cells. **b** PGK1 level in mitochondria and cytosol fraction of in KYSE150 cells, KYSE70 cells, ECA109 cells, and TE-8 cells. **c** The impacts of PGK1 and/or ERK2 on PDHK1 T338 phosphorylation levels in KYSE150 cells, KYSE70 cells, ECA109 cells, and TE-8 cells. **d** The impacts of PGK1 and/or ERK2 on PDH activity in KYSE150 cells (*n* = 36) and ECA109 cells (*n* = 36) (two-sided Student’s *t*-test, mean ± SD). KYSE150: *p* = 0.088, *p = 0.031, ****p = 7.7E–6, *p* = 0.81, *p* = 0.66 from left to right. ECA109: *p = 0.043, *p* = 0.052, ****p = 1.3E–7, *p* = 0.78, *p* = 0.058 from left to right. **e** The impacts of overexpressed and knockdown PGK1 and ERK2 on OCR and ATP production (two-sided Student’s *t*-test, mean ± SD). Twenty-four cell samples were used in the analysis. Top: ****p = 1.6E–8 (PGK1), ****p = 2.6E–12 (PGK1 + ERK2). Bottom: ***p = 7.7E–4. OCR: oxygen consumption rate. **f** The impacts of overexpressed and knockdown PGK1 and ERK2 on ECAR (two-sided Student’s *t*-test, mean ± SD). Sixteen cell samples are used in the analysis. Top: **p = 1.2E–3 (PGK1), ****p = 1.0E–11 (PGK1 + ERK2). Bottom: ***p = 3.9E–7. ECAR: extracellular acidification rate. **g** The impacts of PGK1-overexpression (OE) and/or ERK2-OE on cell proliferation in KYSE150 cells, KYSE70 cells, ECA109 cells, and TE-8 cell (two-sided Student’s *t*-test, mean ± SD). A total of 320 cell samples were used in the analysis. KYSE150: ****p = 1.5E–7 (PGK1), ****p = 1.6E–5 (ERK2), ****p = 2.9E–9 (PGK1 + ERK2). KYSE70: *p = 0.014 (PGK1), **p = 4.5E–3 (ERK2), ****p = 6.8E–5 (PGK1 + ERK2). ECA109: ****p = 5.4E–7 (PGK1), ****p = 3.2E–6 (ERK2), ****p = 2.7E–9 (PGK1 + ERK2). TE-8: **p = 2.1E–3 (PGK1), ***p = 5.0E–4 (ERK2), ***p = 3.2E–4 (PGK1 + ERK2). **h** Gemcitabine inhibits cell proliferation (*n* = 30, two-sided Student’s *t*-test, **p = 4.8E–3, mean ± SD). **i** The impacts of PGK1-OE (left) and PGK1 knockdown (right) on the weight of the xenografts in the KYSE150 cells, ECA109 cells, and TE-8 cells (two-sided Student’s *t*-test, mean ± SD). A total of 130 samples are used in the analysis. Left: KYSE150: ****p = 5.9E–8 (Control and PGK1-OE), ****p = 4.4E–9 (PGK1-OE and PGK1-OE-inhibitor), *p* = 0.17 (Control and PGK1-OE-inhibitor). ECA109: ****p = 2.1E–8 (Control and PGK1-OE), ****p = 4.4E–9 (PGK1-OE and PGK1-OE-inhibitor), *p* = 0.17 (Control and PGK1-OE-inhibitor). TE-8: ****p = 1.3E–7 (Control and PGK1-OE), ****p = 4.0E–8 (PGK1-OE and PGK1-OE-inhibitor), *p* = 1.7E–3 (Control and PGK1-OE-inhibitor). Right: ****p = 8.6E–8 (KYSE150), ****p = 3.4E–7 (ECA109), ****p = 7.6E–8 (TE-8). ****p < 1.0E–4, ***p < 1.0E–3, **p < 0.01, *p < 0.05, ns. > 0.05. Source data are provided as a Source data file.

Next, we investigated the roles of PGK1 in regulating glucose and serine metabolism. Overexpression of PGK1 in KYSE150 cells increased the levels of glycolytic-citrate cycle flux metabolites, including 3-PG, pyruvate, and lactate in glycolysis, and citrate, succinate, and fumarate in citrate cycle (Supplementary Fig. 8c and Supplementary Data 7b). In addition, the levels of serine and glycine were also increased in PGK1-overexpressing cells. Conversely, the knockdown of PGK1 decreased the concentration of metabolites in glycolysis and citrate cycle, as well as serine and glycine (Supplementary Fig. 8d and Supplementary Data 7c). Furthermore, the overexpression of ERK2 led to increased Ser-phosphorylation level, but not Thr- or Tyr- phosphorylation levels of PGK1 in KYSE150, KYSE70, ECA109, and TE-8 cell lines (Fig. 8a). On the contrary, ERK2 could not increase the Ser-phosphorylation level of PGK1 S203 mutant (S203A) (Fig. 8a), indicating that ERK2 phosphorylated PGK1 S203 in ESCC cells. Moreover, the increased Ser-phosphorylation level of PGK1 led to mitochondrial translocation of PGK1 (Fig. 8b), which increased the phosphorylation level of PDHK1 at T338 (Fig. 8c), and decreased pyruvate dehydrogenase (PDH) activity in ERK2 overexpressing cells (Fig. 8d and Supplementary Data 7d). It was also observed that the metabolites of glycolysis and serine metabolism were further increased, while citrate cycle metabolites were decreased in ERK2 overexpression cells (*t*-test, *p* < 0.05) (Supplementary Fig. 8c), suggesting that the overexpression of ERK2 could shut down the pyruvate dehydrogenase complex. Moreover, through Seahorse assay, we found overexpression of PGK1 decreased oxygen consumption rate (OCR) and ATP production, and increased extracellular acidification rate (ECAR) (Fig. 8e, f and Supplementary Data 7e). In contrast, knockdown of PGK1 increased OCR and ATP production, and decreased ECAR (Fig. 8e, f and Supplementary Data 7f). In addition, increased expression of ERK2 further decreased OCR and ATP production, and increased ECAR, in PGK1-overexpression cells (Fig. 8e, f). Collectively, these results indicated that the increased expression and phosphorylation levels of PGK1 synergistically enhanced glycolysis and serine metabolism. Accordingly, we confirmed that co-overexpression of PGK1 and ERK2 in the above four kinds of ESCC cell lines (KYSE150, KYSE70, ECA109, and TE-8) promoted their proliferation most profoundly, compared to cells overexpressing either PGK1 or ERK2 (*t*-test, *p* < 1.0E–4) (Fig. 8g and Supplementary Fig. 8e, and Supplementary Data 7g). In contrast, the knockdown of PGK1 slowed down the cell proliferation, which was further inhibited by double-knock down of PGK1 and ERK2 in those four kinds of ESCC cell lines (*t*-test, *p* < 1.0E–3) (Supplementary Fig. 8f, g and Supplementary Data 7h). Furthermore, unlike PGK1, the overexpression of other glycolytic enzymes, including GAPDH and PGM1, which catalyzed the last and the next step reaction of PGK1, respectively, did not show pro-proliferation effects in KYSE150 cells (*t*-test, *p* < 0.05) (Supplementary Fig. 8h and Supplementary Data 7i). Taken together, these results indicated that the activation of glycolytic enzyme PGK1 was associated with the proliferation of ESCC cells.

It has been reported that pyrimidine deoxynucleoside analog diphosphates (for example, gemcitabine) could be used as PGK1 inhibitors because L-nucleoside analog diphosphates were selectively phosphorylated by PGK1^55^. Therefore, we tested the potential of gemcitabine to inhibit ESCC tumor growth. Firstly, we validated the significant inhibitory effects of gemcitabine on PGK1 (*t*-test, *p* = 4.8E–3) with an IC50 of 16.3 nM (Fig. 8h and Supplementary Fig. 8i, and Supplementary Data 7j) using an in vitro enzymatic assay. Secondly, treating the cultured cells with 50 nmol/L gemcitabine significantly decreased the glycolytic flux (such as pyruvate (*t*-test, *p* = 1.6E–3) and lactate (*t*-test, *p* = 0.014)) and cell proliferation (Supplementary Fig. 8j and Supplementary Data 7k, l). Thirdly, the xenograft growth-promoting ability of PGK1 overexpression was abolished in gemcitabine treated mice bearing either KYSE150 cells (*t*-test, *p* = 4.4E–9), ECA109 cells (t -test, *p* = 2.2E–9), or TE-8 cells (*t*-test, *p* = 4.0E–8), which was consistent with the effects of PGK1 knockdown (*t*-test, *p* (KYSE150) = 8.6E–8, *p* (ECA109) = 3.4E–7, *p* (TE-8) = 7.6E–8) (Fig. 8i and Supplementary Fig. 8k, and Supplementary Data 7m, n). In addition, we found that increased levels of PGK1 phosphorylation, but not PGK1 protein levels, were associated with tumor weight in xenograft TE-8 cells (Supplementary Fig. 8l, m and Supplementary Data 7o). Overall, these observations suggested that enhanced PGK1 expression was associated with early ESCC development.

## Discussion

ESCC is one of the most common malignancies, with a relatively low overall 5-year survival rate (less than 30%). Even though the whole genome sequences of ESCC patients have been obtained, the tumor heterogeneity and lack of understanding of the molecular mechanisms in ESCC progression impose many challenging unmet clinical needs in ESCC. It was speculated that tracking the occurrence and development of early ESCC could provide direct evidence of cancer-driving pathways and molecules in each stage.

Depending on the substage-based model, our study detected precise temporal molecular switches promoting the progression of ESCC at the multi-omics level. The sequence of canonical cancer pathways was also disclosed, which involved ERBB, NOTCH, IGF, cell cycle, DNA repair, PI3K-AKT, mTOR, glycolysis, and Wnt signaling. The co-occurrence mutations of *BOC*, *AKAP9*, and *PCDHB16*, detected as early as in stage 6 and last till the T2 and T3 stages, had positive impacts on DNA repair in stage 6, and glycolysis in the T2 and T3 stages, respectively. To further validate the findings and results in our cohort, we collected another 256 samples as an independent validation cohort from 49 early-stage ESCC patients. The number of stages in the validation cohort was comparable with the main cohort. Comparative analysis showed consistent findings in a time-resolved mode in ESCC progression, and presented that the gradual decrease of keratinization and lipid metabolism revealed that the loss of normal esophagus was an important event in initiating early ESCC. The biomarkers of ESCC tissues, such as ACTA2, TAGLN, POSTN, PSAP, and THBS1^20,21^, were also detected and significantly increased during ESCC progression (Kruskal–Wallis test, FDR < 2.2E–16, stage 9 vs. stage 1 ratio ≥ 2) (Supplementary Fig. 5e, f). These findings provided a temporal dimension and trans-omics dimension in understanding the ESCC progression.

It is generally agreed that carcinogenesis is a chronic process involving several genes and pathways in different stages. The significant genomic aberrations were then translated to proteomic alterations in ESCC progression. Liu et al. also showed that several significantly mutated genes were shared in the earlier and advanced stage of ESCC^7^. Thus, we hypothesized that the mutations were cumulative in ESCC progression, and the key mutation/events which were found in the advanced stages might exist in the earlier stages. In our cohort, we observed that the number of mutations gradually cumulated during ESCC carcinogenesis. The dual-peak of the neo-mutations detected at the Tis stage and the T2 stage, perfectly matched their corresponding pathological phenotypes observed in the clinic, delivering that the mutations were not based on the principle of the linear accumulation model, but surged in certain histopathological stages during the carcinogenesis process. The mutation-surging wave at the advanced stage of ESCC (T2 and T3 stages) in the Fudan cohort indicated that the sudden rise of the mutations at specific stages was regular and would determine the carcinogenesis trend.

The neo-mutation peaks in ESCC progression promoted to divide the carcinogenesis of ESCC into three phases (NT, IEN, A-ESCC). Compared to the IEN and A-ESCC phases, the lowest TMB was observed in the NT phase, in which *TP53* mutation and DNA repair signature were detected. Biological function of the normal esophagus and inflammatory response were the dominant pathways in the NT phase. The Ca^2+^ signal impacts nearly all aspects of cellular life^56^, and is implicated in a variety of processes that are important in tumor progression, such as proliferation and invasiveness^57^. In our cohort, we found the gain of chr3q was characterized in the IEN phase, in which the *cis-*effects genes related to the Ca^2+^ signal and showed positive impacts on DNA replication. Interestingly, the amplification of *TP63* at chr3q28, prominent in the IEN phase, showed positive impacts on DNA replication, implying the combined effects of Ca^2+^ signal and *TP63* amplification on DNA replication in the IEN phase and further indicating the functions of chr3q gain in the transmit process from the NT phase to the IEN phase and on DNA replication in ESCC progression. The highest TMB was detected in the A-ESCC phase, in which the mutation of *MACF1*, a large crosslinker that contributes to cell integrity and cell differentiation^58^, activated Wnt signaling at the multi-omics level. Interestingly, based on the two proteomic clusters (C1 and C2) which were associated with the three phases in ESCC progression, the C2 (relatively malignant cluster) prominent mutation of *AKAP9* upregulated the expression-level of AKAP9 and thus activated PKA, improving the transfer of ATP to ADP and enhancing glycolysis at the protein and phosphoprotein levels (Supplementary Fig. 9). These results had explored that all these pathways followed a very precise temporal order during the carcinogenesis progress of ESCC.

Except for the diverse lifestyles of the countries^2,3^, drinking/smoking habit, gender, and ages are key environmental risks of ESCC^1^, while the molecular mechanism is yet unknown. We applied the NMF algorithm to analyze the mutational signatures of the Fudan cohort and other ESCC cohorts, including the TCGA cohort, Moody’s cohort, etc. The integrated findings revealed that the SBS16 signature, which was associated with the ESCC patients with a drinking habit and *OLFM4* mutation exhibited positive impacts on CDKs activation and thus enhancing DNA replication evidenced by the related markers, indicating the potential medicative of Dinaciclib for drinking ESCC patients. In addition, the APOBEC signature was the dominant signature in the non-drinking/smoking ESCC patients, and the significant mutation of *DCTN2* upregulated the protein level of DCTN2, and displayed positive impacts on RUVBL1 and thus activated DNA replication, implying the potential effects of CB-6644 in the non-drinking/smoking ESCC patients. These results revealed the functions of DNA replication in the IEN phase, whereas the molecular mechanism and clinic strategy were diverse in the ESCC patients with the habits of drinking and non-drinking/smoking.

Furthermore, 746 samples from 114 early ESCC patients in the Fudan cohort allowed us to trace the carcinogenesis lineages of early-stage ESCCs, resulting in six tracks closely related to the clinical feature, including gender, age, and risk habits of drinking/smoking. For example, more female ESCC patients were observed in track 3 (Supplementary Fig. 9). To further validate the results in our cohort, we collected another 256 samples as an independent validation cohort from 49 early-stage ESCC patient, which were then classified into tracks 1–6 with similar molecular characterization. A large-scale, population-based cohort study has shown that drinking/smoking promotes ESCC carcinogenesis^5^. Our study disclosed the carcinogenesis lineage of ESCC patients with drinking/smoking habits (track 6). Integration with the findings in the validation cohort revealed that the immune response pathways, including interleukins signaling, antigen processing and presentation, etc., were the dominant pathways in the ESCC patients with drinking/smoking habits, which was consistent with features of track 6, both in the main cohort and validation cohort, further validating the findings of the personalized tracks. In brief, in addition to the driver waves, this study provided 6 carcinogenesis tracks as references for diverse ESCC clinical therapies.

Proliferative cancer demands a great deal of energy and building blocks, and cancer cells mainly rely on aerobic glycolysis to produce building blocks and energy, known as the Warburg effect^59,60^. In the Fudan cohort, aberrant glycolysis and alterations in its key enzyme, PGK1, which was negatively correlated with overall survival rate, were noticed at the multi-omics level, and promoted ESCC cell proliferation and tumor growth. Through motif prediction network and KSEA results, we found that ERK2 was the top-rank kinase associated with the motif (sP) to activate PGK1 (S203) in ESCC progression. Thus, in this study, we focused on the functions of ERK2 on PGK1 phosphorylation, and the roles of PGK1 (S203) in the carcinogenesis process of ESCC. Glycolysis is a sequence of ten enzyme-catalyzed reactions and links other parallel pathways, including the pentose phosphate pathway, serine de novo synthesis pathway, citrate cycle, etc. Several rate-limiting enzymes determine the overall glycolysis rate, and dysregulated glycolytic enzymes are frequently observed in various cancers^61^. In the current study, increased PGK1 both at the protein and phosphoprotein levels through the tumor progress was observed. PGK1 works in the hub of glycolysis and serine/glycine synthesis, and meanwhile, phosphorylated PGK1 at Ser203 was able to inhibit metabolic flux from glycolysis to the citrate cycle. In addition, overexpression of PGK1 decreased OCR and ATP production, and increased ECAR, further indicating the activation of glycolysis. Therefore, the change in PGK1 activated glycolysis, serine synthesis, and inactivated pyruvate dehydrogenase complex leading to further accumulation of glycolysis metabolites. The present study also revealed that the upstream and downstream enzymes of PGK1 in glycolysis did not provide a strong pro-proliferation effect, unlike PGK1.

In addition, the inhibitory effect of gemcitabine on PGK1 enzymatic activity and cell proliferation was validated through in vitro biochemistry assay, cultured ESCC cells, and xenografts model, in which the levels of PGK1 phosphorylation, but not PGK1 protein levels, were positively associated with tumors weight. Collectively, our study indicated that PGK1 is an important drug target in ESCC, whereas the association of the expression and phosphorylation levels of PGK1 with other kinds of cancer requires further investigation.

However, limits are still represented in our study. The low DNA extraction and peptide extraction of the trace amounts of samples restricted the coverage at the multi-omics level, especially for the limited overlapped samples between genome and phosphoproteome, which is also a challenge for transcriptomic analysis. Owing to the lack of the proteogenomic profiling early-stage of ESCC in the previous studies, the validation of the datasets of other ESCC cohorts relied on the advanced-stage samples of ESCC. In addition, the validations of the impacts of other genes (e.g., AKAP9, MACF1, etc.) in ESCC progression are lacking, even though we believe the comprehensive proteogenomic landscape of early-stage ESCC at the multi-omics level will provide a valuable resource for ESCC and considerable insights into understanding ESCC molecular mechanisms.

In summary, our study depicted the comprehensive genomic, proteomic, and phosphoproteomic map in ESCC progression, and highlighted the key events during the transit process in ESCC progression. We discovered the kinetic waves of the dominant cancer pathways via integrative proteogenomic analysis in the whole process of carcinogenesis. We also uncovered 6 major tracks and their molecular characteristics during the carcinogenesis of ESCC, and illustrated the molecular characterization of environmental risks in ESCC at the multi-omics level (Supplementary Fig. 9). Furthermore, we demonstrated and proposed the value of a drug-targetable protein, PGK1, especially the phosphoprotein, PGK1 S203, at the multi-omics map. We believe this study provides insights into understanding the architecture of ESCC progression and enables advances in promoting the diagnostics and therapeutics to manage ESCC.

## Methods

### Patient samples of early ESCCs

#### Construction of the ESCC cohort

Three hundred consecutive patients presumed to have esophageal lesions underwent ESD therapy from January 2018 to December 2018 at Zhongshan Hospital, Fudan University. There were no biases in selecting patients, and none of the patients had received any prior treatment, such as radiotherapy or chemotherapy. One hundred and fourteen early ESCC cases were eligible for the establishment of the intended study cohort. Among the 186 excluded patients, 21 were diagnosed with non-tumor lesions, 26 had stromal tumors, 86 patients were precluded due to the unavailability of their normal tissue samples, and 53 samples failed to pass the pathological quality check, such as tumor cell ratio <80%. Subsequently, 40 advanced ESCC cases (*n* = 16 for T2 and 24 for T3) were screened after surgical resection without neoadjuvant therapy. All cases were staged according to the 8th edition of the American Joint Committee on Cancer (AJCC) TNM staging system.

As for pathology quality control, it was our primary concern for a strict pathology classification. The complex pathological staging was based on the morphological observation according to the 8th edition of the AJCC TNM staging system. Notably, all early-stage ESCC samples in our cohort were dissected with 3 mm thick and stood up one by one in the embedding, and then marked in the H&E-stained sections. The H&E-stained sections were reviewed and evaluated by two or three experienced gastrointestinal pathologists who would mark them according to the proportion of tumor cells (Score 0 = 0%; 0% < Score 1 < 20%; 20% ≤ Score 2 < 40%; 40% ≤ Score 3 < 60%; 60% ≤ Score 4 < 80%; 80% ≤ Score 5 ≤ 100%). The tumor purity of all samples was defined as Score 5, indicating the high quality of all samples of our cohort. The present study was carried out in compliance with the ethical standards of Helsinki Declaration II and approved by the Institution Review Board of Fudan University Zhongshan Hospital (B2019-200R). All the patients’ samples were obtained with Zhongshan’s approval of the Research Ethics Committee. Written informed consent was provided by all participants before any study-specific investigation was performed. Each sample was assigned a new research ID, and the patient’s name or medical record number used during hospitalization was de-identified. Clinical information of individual patients, including age, gender, smoking status, and substages, were listed in Supplementary Data 1a.

According to the WHO and Japanese pathology diagnostic criteria, all the substages in our early ESCC cohort were contained in four TNM stages: T0 (normal epithelial, *n* = 114), T1 (T1a/b cancer, *n* = 114), T2 (*n* = 16), and T3 (*n* = 24) (Supplementary Table 1). T1 was sub-classified into hyperplasia stage (2_1, *n* = 114), mild and/or moderate dysplasia stage (2_2, *n* = 92), Tis stage (3_1 (*n* = 61), 3_2 (*n* = 73), 3_3 (*n* = 67), 3_4 (*n* = 19), and 3_5 (*n* = 39)), lamina propria cancer stage (including m2 stage, 4_1 (*n* = 61), 4_2 (*n* = 18), and 4_3 (*n* = 7)), and muscularis mucosa stage (including m3 stage, 5_1 (*n* = 14), 5_2 (*n* = 9), and 5_3 (*n* = 9)), and submucosal invasion cancer stage (sm stage), namely sm stage a (6_1 (*n* = 5), 6_2 (*n* = 5), and 6_3 (*n* = 7)), and sm stage b (7_1 (*n* = 12), 7_2 (*n* = 9), and 7_3 (*n* = 11)) (Supplementary Table 2). According to the infer of the peaks of neo-mutation, all the samples were distributed to three phases: NT phase (normal tissue stage and hyperplasia stage), IEN phase (Tis stage to submucosa stage), and A-ESCC phase (T2 and T3 stages).

All 786 samples were subjected to proteomic profiling. Owing to the definite volume of the samples of the early ESCC cohort, only 145 samples (from 58 ESCC patients) were adequate for phosphoproteomic profiling: normal tissue (*n* = 20), hyperplasia stage (*n* = 37), Tis stage (*n* = 31), lamina propria cancer stage (*n* = 14), muscularis mucosa stage (*n* = 5), sm stage a (*n* = 3), sm stage b (*n* = 9), T2 stage (*n* = 10), and T3 stage (*n* = 16) (Supplementary Table 4). In addition, only 102 samples (from 46 ESCC patients) covering 20 substages: stage 1 (*n* = 12), hyperplasia stage 2_1 (*n* = 8), hyperplasia stage 2_2 (*n* = 4), Tis stage 3_1 (*n* = 2), Tis stage 3_2 (*n* = 4), Tis stage 3_3 (*n* = 5), Tis stage 3_4 (*n* = 1), Tis stage 3_5 (*n* = 6), lamina propria cancer stage 4_1 (*n* = 7), muscularis mucosa stage 5_1 (*n* = 3), muscularis mucosa stage 5_3 (*n* = 1), sm stage (a) 6_1 (*n* = 1), sm stage (a) 6_2 (*n* = 2), sm stage (a) 6_3 (*n* = 1), sm stage (b) 7_1 (*n* = 4), sm stage (b) 7_2 (*n* = 3), sm stage (b) 7_3 (*n* = 4), T2 stage (*n* = 15), and T3 stage (*n* = 19) were adequate for WES.

#### Validation of an independent ESCC cohort

Two hundred and fifty-six samples were collected as another independent validation cohort, from 49 early-stage ESCC patients (Supplementary Table 3 and Supplementary Data 6d). The number of stages in the validation cohort was proportionable compared with the main cohort. The early-stage ESCC patients of the validation cohort were presumed to have esophageal lesions and underwent ESD therapy from January 2019 to December 2019 at Zhongshan Hospital, Fudan University. There were no biases in selecting patients, and none of the patients had received any prior treatment, such as radiotherapy or chemotherapy. All the patient samples were obtained with Zhongshan’s approval of the Research Ethics Committee. Written informed consent was provided by all participants before any study-specific investigation was performed. Each sample was assigned a new research ID, and the patient’s name or medical record number used during hospitalization was de-identified.

#### Processing of FFPE specimens

All the FFPE specimens were prepared and provided by Zhongshan Hospital, Fudan University. For clinical sample preparation, slides (10 μm thick) from FFPE blocks were macro-dissected, deparaffinized with xylene and washed with ethanol. One 3-μm-thick slide from FFPE blocks was sectioned for H&E stained. All the selected specimens were scraped according to the substages, which were evaluated and confirmed by two or three experienced and board-certified gastrointestinal pathologists, and materials were aliquoted and stored at −80 °C until further processing.

### WES

WES was performed by Novogene Co., LTD. DNA from FFPE tumor tissue samples was collected for WES and matched germline DNA was obtained from non-tumor tissue samples. One hundred and two samples from 46 cases were analyzed. Paired-end sequencing (PE150) was performed on Illumina HiSeq platform (Illumina Novaseq 6000) with the mean coverage of the samples conducted in WES was 131×, and the mean volume of raw data was 14.0G, which was consistent with other literature studies^62^. The resulting sequence libraries (the paired-end sequence and insert DNA between two ends) were quantified with a Qubit 2.0 (Thermo Fisher), and the insert size was determined using an Agilent 2100 Bioanalyzer. The original fluorescence image files obtained from the Hiseq platform are transformed into short reads (raw data) by base calling. These short reads are recorded in FASTQ format, which contains sequence information and corresponding sequencing quality information. Base-calling was used to obtain the raw data (sequenced reads, mean (raw data) of all samples was no less than 12G) from the primary image data.

### DNA extraction and DNA qualification

One hundred and two samples from 46 cases were analyzed by WES. All the samples were firstly dewaxing with dimethylbenzene, and then DNA degradation and contamination were monitored on 1% agarose gels. DNA concentration was measured by Qubit® DNA Assay in Qubit® 2.0 Flurometer (Invitrogen, USA, Catalog: 5190-8863). A total amount of at least 0.6 μg genomic DNA per sample was used as input for DNA sample preparation.

### Library preparation

A total amount of 0.6 μg genomic DNA per sample was used as input for DNA sample preparation. Sequencing libraries were generated using Agilent SureSelect Human All Exon kit (Agilent Technologies, CA, USA, Catalog: 5190-8863) following the manufacturer’s recommendations and index codes were added to each sample.

Fragmentation was carried out by hydrodynamic shearing system (Covaris, Massachusetts, USA) to randomly generate 180–280 bp fragments. The remaining overhangs were converted into blunt ends via exonuclease/polymerase activities. After adenylation of 3’ ends of DNA fragments, adapter oligonucleotides were ligated. DNA fragments with ligated adapter molecules on both ends were selectively enriched in a PCR reaction. After PCR reaction, libraries hybridize with the liquid phase with a biotin-labeled probe, then use magnetic beads with streptavidin to capture the exons of genes. Captured libraries were enriched in a PCR reaction to add index tags to prepare for sequencing. Products were purified using AMPure XP system (Beckman Coulter, Beverly, USA) and quantified using the Agilent high-sensitivity DNA assay on the Agilent Bioanalyzer 2100 system.

The clustering of the index-coded samples was performed on a cBot Cluster Generation System using Hiseq PE Cluster Kit (Illumina) according to the manufacturer’s instructions. After cluster generation, the DNA libraries were sequenced on Illumina Hiseq platform and 150 bp paired-end reads were generated.

### QC of WES data processing and analysis

The following criteria were used to ensure high-quality clean data for the downstream bioinformatics analyses:

1. A paired read was discarded if at least one read contained adapter contamination (>10 nucleotides aligned to the adapter), allowing ≤10% mismatches.
2. A paired read was discarded if >10% of bases were uncertain in at least one read.
3. A paired read was discarded if the proportion of low-quality (Phred quality <5) bases was over 50% in either one read.

At the same time, QC statistics, including total number of reads, raw data, raw depth, sequencing error rate, percentage of reads with Q30 (the percent of bases with a Phred-scaled quality score) greater than 30 and QC content distribution were calculated and summarized.

### Reads mapping to the reference sequence

Valid sequencing data were mapped to the reference human genome (UCSC hg19) by Burrows–Wheeler Aligner (BWA) software^63^ to get the original mapping results stored in BAM format. If one or one paired read(s) were mapped to multiple positions, the strategy adopted by BWA was to choose the most likely placement. If two or more most likely placements were presented, BWA randomly picked one. Then, SAMtools^64^ and Picard (http://broadinstitute.github.io/picard/) were used to sort BAM files and perform duplicate markings, local realignment, and base quality recalibration to generate the final BAM file for computation of the sequence coverage and depth. The mapping step was very difficult due to mismatches, including true mutation and sequencing errors, and duplicates resulting from PCR amplification. These duplicate reads were uninformative and should not be considered as evidence for variants. We used Picard to mark these duplicates for subsequent analysis.

### Detection and calling of somatic mutations

BWA and Samblaster were used for genome alignment, and muTect Software^64^ was used for identifying the Somatic SNV sites, whereas Strelka^65^ was used to detect the Somatic InDels. Control-FREEC was used to detect SCNAs. SAMtools mpileup and bcftools were used for the variant calling and to identify the SNPs and InDels. Statistical analysis included two-tailed Student’s *t*-test and Fisher’s exact test.

### GISTIC and MutSig analysis

To identify significantly amplified or deleted focal-level and arm-level events, we used The Genomic Identification of Significant Targets in Cancer (GISTIC) algorithm^66^ to the genomic data in the Fudan cohort, and the *Q* value <0.25 was considered significant. The genes of each sample were assigned a threshold copy number level to reflect the magnitude of its deletion or amplification. These are integer values ranging from −2 to 2, where 0 means no amplification or deletion of magnitude greater than the threshold parameters described above. Amplifications are represented by positive numbers: 1 means amplification above the amplification threshold; 2 means amplification larger than the arm-level amplification observed in the sample. Deletions are represented by negative numbers: −1 means deletion beyond the threshold, and −2 means deletions greater than the minimum arm-level copy number observed in the sample.

### The gain of neo-mutations

To investigate the mutation at all stages during the progression of ESCC, the numbers of total mutations and neo-mutations at each stage were counted. The number of neo-mutations at a certain stage could reflect the impacts of mutations in the progression of ESCC, demonstrating the genomic characteristics of the early-stage ESCC; therefore, we estimated the gain of neo-mutations. The mutation frequency was estimated by the ratio of the number of mutated samples vs. the number of total samples^18^. Here, the neo-mutation represented the mutations appearing at a certain stage, but was not identified in earlier stages. For example, the mutation of *FAT4* was detected in the Tis stage, but not in the hyperplasia stage.

### Mutational signature analysis

Based on the single nucleotide substitution and its adjacent bases pattern of samples, frequencies of 96 possible mutation types for each sample could be estimated. NMF algorithm was used to estimate the minimal components that could explain maximum variance among samples. Then each component was compared to mutation patterns of 30 validated cancer signatures reported from the COSMIC database to identify cancer-related mutational signatures in the Fudan cohort and other ESCC cohorts. Cosine similarity analysis^66^ was used to measure the similarity between components and signatures, which ranged from 0 to 1, indicating maximal dissimilarity to maximal similarity.

### Analysis of somatic SNVs signatures

#### SBS16 signature enrichment analysis

To identify SBS and portray the contribution across the whole genome based on WES data, we applied the analysis procedure as an R/CRAN package sigminer (Version 2.0.1) (https://cran.r-project.org/web/packages/sigminer/), to extract and analyze mutational signatures for genomic variations, providing valuable insights into cancer study. The most common criterion to estimate the signature number is the cophenetic correlation coefficient. Sigminer package (Version 2.0.1) can provide both relative and absolute exposures of cancer signatures. In addition, we performed the OS survival analysis for SBS16 signature (log-rank test, *p* < 0.05), which was referred from in TCGA cohort genomic dataset^15^ (https://www.cbioportal.org/).

#### APOBEC enrichment estimation

APOBEC-driven mutagenesis is associated with C>T transition events occurring in TCW motif. We applied plotApobecDiff (https://rdrr.io/bioc/maftools/man/plotApobecDiff) to estimate APOBEC enrichment scores estimated, by which all the samples for WES were grouped into two: APOBEC enriched and non-APOBEC enriched. The same methods are applied in previously published studies^67^.

### Defining cancer-associated genes (CAGs)

CAGs were compiled from genes defined by Bailey et al.^68^ and cancer-associated genes listed in Mertins et al.^69^ and adapted from Vogelstein et al.^70^. The list of genes is provided in Supplementary Data 6g.

### Analysis of SCNAs and the impacts on protein expressions

SCNAs analysis was performed using the WES-derived BAM files that were processed in the somatic mutation detection pipeline. These BAM files were further processed by the R package copywriteR (version 1.18.0), which used off-target WES read to infer copy number values. In this study, we used the multiomicsViz (version 1.6.0) in R (version 3.5.1) to perform the correlation of genomics and proteomics data. Correlations between SCNAs and proteome (with proteome data mapped to genes, by choosing the most variable protein as the gene-level representative) were determined using Spearman’s correlation of common genes present in SCNA-proteome (3474 mutations/proteins), which was the key event in esophageal carcinogenesis. Only genes or proteins with <66.7% NAs were considered for the analysis, and protein IDs were mapped to gene names.

### Protein extraction and trypsin digestion

All samples of early and advanced ESCC patients were dissected with microdissection and collected in 1.5 mL EP tubes, and then stored in the refrigerator at −80 °C. The thickness of every FFPE piece is 10 μM, and every substage is no more than 10,000 cells.

Fifty μL TCEP buffer (2% deoxycholic acid sodium salt (Solarbio, Catalog: D8330)), 40 mM 2-chloroacetamide (ALDRICH, Catalog: 22790-250G-F), 100 mM tris-phosphine hydrochloride (AMRESCO, Catalog: 0497), 10 mM (2-carboxyl)-phosphine hydrochloride (ALDRICH, Catalog: 4706-10G), 1 mM phenylmethylsulfonyl fluoride (AMRESCO, Catalog: M145-5G) mixed with MS water (J.T. Baker, Catalog: 4218-03), PH 8.8) were added into 1.5 mL EP tubes with prepared samples, and then heated at 99 °C metal bath for 30 min. After cooling to room temperature, 3 μg trypsin (Promega, Catalog: V528A) was added into each tube and digested for 18 h at 37 °C incubator. Then, 13 μL 10% formic acid (FA) (Sigma, Catalog: F0507) was added into each tube and made vortex for 3 min, and then centrifuged for 5 min (12,000 g). After that, a new 1.5 mL tube with 350 μL buffer (0.1% FA in 50% acetonitrile [ACN] (J.T. Baker, Catalog: 9830-03)) is needed for collecting the supernatant for extraction (vortex for 3 min, and then 12,000 g centrifuged for 5 min). And then, the supernatant was transferred into a new tube for drying at 60 °C in a vacuum drier. After drying, 100 μL 0.1% FA was needed for dissolving the peptides and vortex for 3 min, and then centrifuged for 3 min (12,000 g). The supernatant was picked into a new tube and then desalinated. Before desalination, the activation of pillars with two slides of 3M C18 disk is required, and the lipid is as follows: 90 μL 100% ACN twice, 90 μL 50 and 80% ACN once in turn, and then 90 μL 50% ACN once. The supernatant of the tubes was then loaded into the pillar twice, and decontamination with 90 μL 0.1% FA twice. Lastly, 90 μL elution buffer (0.1% FA in 50% ACN) was added into the pillar for elution twice and only the effluent was collected for MS. Then the collection liquid was put at 60 °C in a vacuum drier for drying (~1.5 h).

### Proteome/phosphoproteome analysis in LC-MS/MS analysis

For the proteomic profiling of samples, peptides were analyzed on a Q Exactive HF-X Hybrid Quadrupole-Orbitrap Mass Spectrometer (Thermo Fisher Scientific, Rockford, IL, USA) coupled with a high-performance liquid chromatography (HPLC) system (EASY nLC 1200, Thermo Fisher). Dried peptide samples re-dissolved in Solvent A (0.1% FA in water) were loaded to a 2-cm self-packed trap column (100-μm inner diameter, 3 μm ReproSil-Pur C18-AQ beads, Dr. Maisch GmbH) using Solvent A and separated on a 150-μm-inner-diameter column with a length of 15 cm (1.9 μm ReproSil-Pur C18-AQ beads, Dr. Maisch GmbH) over a 150 min gradient (Solvent A: 0.1% FA in water; Solvent B: 0.1% FA in 80% ACN) at a constant flow rate of 600 nL/min (0–150 min, 0 min, 4% B; 0–10 min, 4–15% B; 10–125 min, 15–30% B; 125–140 min, 30–50% B; 140–141 min, 50–100% B; 141–150 min, 100% B). The eluted peptides were ionized under 2.0 kV and introduced into mass spectrometer). MS was performed under a data-dependent acquisition mode. For the MS1 Spectra full scan, ions with m/z ranging from 300 to 1400 were acquired by Orbitrap mass analyzer at a high resolution of 120,000. The automatic gain control (AGC) target value was set as 3E6. The maximal ion injection time was 80 ms. MS2 Spectra acquisition was performed in the ion trap mode at a rapid speed. Precursor ions were selected and fragmented with higher energy collision dissociation (HCD) with a normalized collision energy of 27%. Fragment ions were analyzed by the ion trap mass analyzer with the AGC target at 5E4. The maximal ion injection time of MS2 was 20 ms. Peptides that triggered MS/MS scans were dynamically excluded from further MS/MS scans for 12 s. The same methods and parameters have been applied in other published studies^71^.

For the phosphoproteomic samples, peptides were analyzed on a Q Exactive HF-X Hybrid Quadrupole-Orbitrap Mass Spectrometer (Thermo Fisher Scientific) coupled with a HPLC system (EASY nLC 1200, Thermo Fisher Scientific). Dried peptide samples re-dissolved in Solvent A (0.1% formic acid in water) were loaded onto a 2-cm self-packed trap column (100 μm inner diameter, 3 μm ReproSil-Pur C18-AQ beads, Dr. Maisch GmbH) using Solvent A and separated on a 150-μm-inner-diameter column with a length of 30 cm (1.9 μm ReproSil-Pur C18-AQ beads, Dr. Maisch GmbH) over a 150-min gradient (buffer A: 0.1% formic acid in water; buffer B: 0.1% formic acid in 80% ACN) at a constant flow rate of 600 nL/min (0–150 min, 0 min, 4% B; 0–10 min, 4–15% B; 10–125 min, 15–30% B; 125–140 min, 30–50% B; 140–141 min, 50–100% B; 141–150 min, 100% B). The eluted phosphopeptides were ionized and detected by a Q Exactive HF-X Hybrid Quadrupole-Orbitrap mass spectrometry. Mass spectra were acquired over the scan range of m/z 300–1400 at a resolution of 120,000 (AUG target value of 3E+06 and maximum injection time 80 ms). For the MS2 scan, HCD fragmentation was performed at a normalized collision energy of 30%. The MS2 AGC target was set to 5E4 with a maximum injection time of 100 ms. The peptide mode was selected for monoisotopic precursor scan, and charge state screening was enabled to reject unassigned 1+, 7+, 8+, and >8+ ions with a dynamic exclusion time of 40 s to discriminate against previously analyzed ions between ±10 ppm.

### Phosphopeptide enrichment and analysis

All qualified profiling data were processed at firmiana platform against the human RefSeq protein database (updated on July 4, 2013) in the National Center for Biotechnology Information (NCBI). Owing to the definite volume of the samples of the early ESCC cohort, only 145 samples (from 41 ESCC patients) were found to be adequate.

The phosphoproteome samples were prepared by Fe-NTA Phosphopeptide Enrichment Kit (Thermo, Catalog: A32992) according to the manufacturer’s instructions. Briefly, 2 mg peptides were resuspended in 200 μL binding/wash buffer and loaded to the equilibrated spin column. The resin was mixed with the sample by gently tapping. The mixture was incubated for 30 min and centrifuged at 1000 × g for 30 s to discard the flowthrough. The column was then washed with 200 μL of binding/wash buffer and centrifuged at 1000 × g for 30 s three times and washed with 200 μL of LC-MS grade water one more time. The phosphopeptide was eluted with 100 μL of elution buffer and centrifuged at 1000 × g for 30 s two times. Phosphopeptides were dried down for LC-MS/MS analysis.

### Quantification of global proteome data and phosphoproteome data

In our study, all MS raw files were processed at firmiana platform^72^ (a one-stop proteomic cloud platform: http://www.firmiana.org). Briefly, all MS raw files were searched against the NCBI human RefSeq protein database (updated on July 4, 2013, 32,015 entries) in Mascot search engine (version 2.3, Matrix Science Inc). Trypsin was used as the proteolytic enzyme allowing up to two missed cleavages. Carbamidomethyl (C) was considered as a fixed modification. For the proteome profiling data, variable modifications were oxidation (M) and acetylation (Protein N-term). For the phosphoproteome data, variable modifications were oxidation (M), acetylation (Protein N-term) and phospho (S/T/Y). All the identified peptides were quantified at firmiana platform with peaks area derived from their MS1 intensity. The mass tolerances were 20 ppm for precursor and 50 mmu for the product collected by Q Exactive HF-X, which has been applied in previously published studies^71^. Precursor ion score charges were limited to +2, +3, and +4. The FDRs of the peptide-spectrum matches and proteins were set at a maximum 1%. The same cutoff strategies of FDR have been widely used in recently published researches^73,74^. Label-free protein quantifications were calculated in our cohort, that so-called iBAQ algorithm^19,75^, which divided the protein abundance (derived from identified peptides’ intensities) by the number of theoretically observable peptides. Then the FOT, defined as a protein’s iBAQ divided by the total iBAQ of all identified proteins within one sample, was used to represent the normalized abundance of a particular protein across samples.

### Data imputation

For the missing values (NAs) in our study, we first applied a match between runs (MBR) algorithm^76,77^ in this study, which has been proven to be an effective technique to fill the missing values, which was widely used in other proteomic studies^78^. In detail, we built a dynamic regression function based on commonly identified peptides in samples. According to correlation value *R*^2^, linear or quadratic function was applied for regression to calculate retention time (RT) of corresponding hidden peptides, and check the existence of the extracted ion chromatogram (XIC) based on the m/z and calculated RT. The function evaluated the peak area values of those existed XICs. These peak area values are considered as parts of corresponding proteins. This strategy has been applied in other published proteomic studies^79,80^.

At proteomic profiling of 786 samples, all the proteome data were processed as follows: E1 (10,913 GPs): all the proteins were required to have at least two unique strict peptide, and we excluded keratins proteins of which the maximum FOT in all 786 experiments were less than 1.0E–5 in FOT; E2 (6,885 GPs): GPs were identified in more than 20% samples of each substage, and the FOT of all proteins whose FOT values were less than 1.0E–5 were replaced with 1.0E–5 to adjust small values, which is also applied in other published proteomic studies^18,79^.

In our study, the trypsin digestion and mass spectrometric analysis of the forty-two HEK293T cell samples (Cat# CRL-11268 from ATCC, RRID: CVCL_QW54) were consistent with the methods of early ESCC sample in our cohort, and then applied to assess the quality control of the performance of MS, of which the measurement is often applied in proteogenomic studies^18^. In our study, Spearman’s correlation coefficient of HEK293T cells (*n* = 42) was 0.91, indicating the consistent stability of our MS platform.

### The unified terms for quantitative analysis

#### The unified terms for quantitative analysis at the genome level

As for the correlation analysis (1) between the mutation characteristics and three phases of ESCC, (2) between the different clinic features of ESCC patients (e.g., *MACF1*), the terms of findings/results of the related correlations were uniformly defined as “prominent/ordinary” with the measurements of two-sided Fisher’ exact test/Chip-seq test (*p* < 0.05) and proportions, including the mutations (e.g., *TP53* and *MACF1*), mutational signatures (e.g., SBS16 and APOBEC), etc. The same methods of the terminology were used for proteogenomic analysis in CPTAC^81^ and Wu’s team^82^.

As for the genome-proteome combined analysis, the terms of the findings/results of the impacts of mutations (e.g., *AKAP9*, *MACF1*) on their counterpart proteins/phosphoproteins expression, were uniformly defined as “upregulate/downregulate” with the measurements of statistic test (two-sided Student’s *t*-test/Wilcoxon rank-signed test) and the fold change (≥2 and ≤0.5 for upregulated and downregulated, respectively). The terms of findings/results of the impacts of mutations (e.g., *AKAP9*, *MACF1*) on other proteins/phosphoproteins expression and the related pathways, were uniformly defined as “positive/negative impacts” with the measurements of statistic test (two-sided Student’s *t*-test/Wilcoxon rank-signed test) and the fold change (≥2 and ≤0.5 for positive impacts and negative impacts, respectively). The same methods of the terminology were applied for proteogenomic analysis in CPTAC^81,83^.

#### The unified terms for quantitative analysis at the proteome and phosphoproteome levels

As for the analysis of the proteins/phosphoproteins expressions and identifications in the comparisons between two group comparisons at proteome and phosphoproteome levels (e.g., two proteomic clusters, mutation group vs. WT group, etc.), the terms of the findings/results were uniformly defined as “overrepresent/underrepresent” with the measurement results of statistic test (two-sided Student’s *t*-test/Wilcoxon rank-signed test) and the fold change (≥2 and ≤0.5 for overrepresented and underrepresented, respectively). The same methods of the terminology were also applied in our previous study^71^ and CPTAC^81^.

As for the analysis of the proteins/phosphoproteins expressions and identifications among three or more group comparisons at proteome and phosphoproteome levels (e.g., 3 phases, 9 stages, 8 waves, etc.), the terms of the findings/results were uniformly defined as “increase/decrease” with the measurement results of statistic test (Kruskal–Wallis test) and fold changes (≥2 and ≤0.5 for increase and decrease, respectively). The same methods of the terminology were also applied in our previous study^84^ and Zeng’s team^85^.

As for the correlation analysis between two proteins/phosphoproteins (e.g., AKAP9 and PRKACA, MACF1 and GSK3B, etc.), the terms of the findings/results were uniformly defined as “positive/negative association” with the measurement results of correlation coefficients (two-sided Pearson’s correlation) and *p* values (<0.05). The same methods of the terminology were also applied in our previous study^84^ and CPTAC^86,87^. In addition, the outliers and missing values were excluded from calculating the correlations.

As for the enrichment analysis of the proteins/phosphoproteins (e.g., cluster 1 overrepresented proteins, etc.), the terms of the findings/results were uniformly defined as “dominant/recessive” with the measurement results of adjust. FDR (<0.05) and/or proteins/phosphoproteins counts. The same methods of the terminology were also applied in our previous study^71^ and CPTAC^88^.

### Batch effect analysis

The hierarchical clustering and principal component analyses were implemented by programming language R (version 4.0.2) to assess the batch effects in our proteome dataset with respect to the following two variables: batch identity and sample type (substage). For the hierarchical clustering analysis, the pair-wise Spearman’s correlation coefficients of the same substage samples were investigated. The samples of the same type exhibited a high similarity, whereas samples of different types clearly differed. There was no clear association between the batch intensity and correlation coefficients. Furthermore, we used the average linkage algorithm with one minus Spearman’s correlation coefficient as the dissimilarity measure. In the global heatmap in our study, each protein expression value in the global proteomic expression matrix was transformed into a *Z*-score across all samples. For the sample-wise and protein-wise clustering, the distance was set as “Euclidean” distance, and the weight method was “complete”. The *Z*-score-transformed matrix was clustered using R package: pheatmap (version 1.0.12).

### Interpretation of the phases, clusters, waves/panels, and tracks

The three phases consisted of the NT phase (stages 1 and 2), IEN phase (stage 3 last till to stage 7), and A-ESCC phase (T2 and T3 stage), which were based on the peaks of the gain of neo-mutations and the histopathological stages in ESCC progression. In our cohort, we found the number of the neo-mutations peaked at stage 3 (Tis stage) and the T2 stage, allowing us to explore the key events in ESCC progression.

The two clusters were based on the distinctive proteomic characterization through consensus clustering analysis, which were associated with the subclassification of stages in ESCC progression. Specifically, C1 included stages 1 and 2, and C2 contained the rest of the (sub)stages, including the T2 and T3 stages. The proteomic clusters provided us a chance to explore the different molecular characterizations of the benign and relative malignant ESCC at the multi-omics level.

The substage-based eight dynamic waves/panels were shown to portray the carcinogenesis path in ESCC progression in a time-resolved mode at the multi-omics level. The eight dynamic waves/panels revealed the substage-specific molecular characteristics and provided potential candidates for ESCC malignancy.

The six tracks represented the personalized lineages of early-stage ESCC patients at the multi-omics level, which were closely associated with various clinical features. For example, track 3, featured with cell cycle, had the highest proportion of female patients (29%). Track 6, featured as immune response, had the highest proportion of patients with drinking/smoking habits (18%).

### Differential proteomics analysis

A SAM^42^ analysis identified 2922 differential proteins in C1 and C2, which were identified based on all 786 samples from 114 early-stage and 40 advanced-stage ESCC patients. The statistical analysis was performed with two-tailed Student’s *t*-test on overlapping samples to determine the differential abundance of proteins between two clusters and diverse tracks, in which statistical significance (two-sided Student’s *t*-test, FDR < 0.05, and differential expression C2 vs. C1 ratio ≥ 2 or ≤ 0.5) was considered in the differential analysis. The two-tailed Student’s *t*-test was used for statistical analysis. Proteins with no NAs in at least 20% of samples were considered in each substage, in which NAs which was assigned to 1.0E–5, which was applied in other studies^79^. In detail, the data type of SAM analysis was set as “two-class unpaired”, delta value was set respectively to meet FDR < 0.01, and the “standard” (t-statistic) was used. The *p* values were adjusted and set the q-values of DEPs. The DEPs were defined if they met the following criteria: (1) *q* value less than 0.01, and (2) fold change (FC, C2 vs. C1 ratio) was either no less than 2 (≥2) or no more than 0.5 (≤0.5). As a result, a total of 2922 DEPs were detected.

When comparing the DEPs of 22 substages, we focused on the substages’ highly expressed proteins (one substage versus all other substages), which were then enriched by GO/KEGG database^89^/Reactome^90^. We then annotated the signaling pathways (FDR < 0.05) and manually checked the pathway-associated proteins, which were then estimated whether they were significantly associated with the 22 substages of ESCC (Kruskal–Wallis test).

In a differential analysis of proteins in ESCC progression (gradually decreased or increased) at the protein level, the highly expressed proteins of each substage/track/panel were screened, in which the differential expressed ratio and adjust. FDR (Kruskal–Wallis test) was considered. Statistical analysis was performed in R (version 3.5.1).

### Pathway enrichment analysis

To investigate the dominant signaling pathways of 2 clusters, 6 tracks, and 22 substages, we used gene sets of molecular pathways from GO/KEGG^89^/Reactome^90^ databases to compute the pathway. Statistical significance was considered when FDR was less than 0.05. The differential score (*Q*) is obtained as signed –log_10_ FDR.

To assess the impacts of the mutations, we applied GSEA for pathway enrichment analysis^91^. GSEA evaluated and determined whether priori defined sets of genes show statistically significant, cumulative changes in gene expression that are correlated with a specific phenotype. To assess the impacts of the mutations (e.g., *OLFM4*, *DCTN2*, *MACF1*, *AKAP9*, *EPAS1*, *EPHA3*, *STAG2*, *USP6*), the samples grouped were subjected to GSEA, respectively. The represented proteins were identified in all samples (FDR < 0.01, unique peptides ≥ 2). Molecular Signatures Database (MSigDB) of KEGG gene sets (C2) was used for enrichment analysis. FDR value of 0.05 was used as a cutoff. The enrichment score in GSEA was calculated by first ranking the proteins from the most to least significant with respect to the two phenotypes (i.e., Mut and WT); the entire ranked list was then used to assess how the proteins of each gene set were distributed across the ranked list.

### Principal component analysis

We performed PCA on a total of 6885 proteins of 786 samples to illustrate the global proteomic difference among the 22 ESCC substages. The PCA function under the scikit-learn R package was implemented for unsupervised clustering analysis with the parameter “n_components = 2” on the expression matrix of global proteomic data. A colored ellipse represented the 95% confidence coverage for each group, calculated based on the mean and covariance of points in each specific substage.

### Consensus clustering analysis of proteomics data

The protein expression matrix of the 786 samples was used to identify the proteomic subtypes using the consensus cluster method. Consensus clustering was performed using the ConsensusClusterPlus R package (ConsensusclusterPlus, version 1.46.0)^92^ with E2 proteins (*n* = 6885). Consensus Cluster Plus parameters were reps = 1000, pItem = 0.8, pFeature = 1, clusterAlg = “km”, distance = “euclidean”, plot = “PDF”. Euclidean distance and 1000 repetitions in the range of 2–10 clusters. The consensus matrix of κ = 10 showed clear separation among clusters. The empirical cumulative distribution function plot initially showed optimal separation. Clustering by κ = 2 appeared to have the most obvious cut between clusters and showed a significant association with the pathological substages. Taken together, proteome clusters were defined using k-means consensus clustering with  κ = 2. As summarized in Supplementary Fig. 6a, e, the clustering analysis of the samples (vertical column) by protein abundance (horizontal rows) classified all samples as two proteomic clusters defined by silhouette analyses.

### Survival analysis

To investigate the impacts of mutations on protein expressions and the development of ESCC carcinogenesis, identified common genes in genomic and proteomic data of biological pathways were screened to perform the survival analysis. The data associated with OS information was referenced to other ESCC cohorts. In addition, Kaplan–Meier survival curves (log-rank test) were used for OS analysis. *p* value (less than 0.05) for significance was used. Owing to the lack of proteomics data of early ESCC, the OS data (RNA-seq, *n* (ESCC) = 81) of PGK1 was downloaded from the TCGA database^93^ (https://portal.gdc.cancer.gov).

### Trajectory inference methods and tracks analysis

We used the monocle (version 2.10.1) and trajectory inference methods to trace the carcinogenesis lineages in 114 early-stage ESCC patients. Firstly, the proteins (E1, *n* = 9,741) of all 746 samples (114 early ESCC cohort) were used. In addition, the proteins with mean expression over 1.0E–1 were highlighted and screened. The dataset was clustered and pre-prepared by t-distributed stochastic neighbor embedding using a Barnes–Hut implementation with Rtsne (version 0.15) in R (version 3.5.1). All the substages of each early ESCC patient were considered as the pseudotime to construct the trajectory of each early ESCC patient. In the end, the trajectory of each ESCC patient was revealed; and then, nine groups were determined by the number of nodes and bifurcations, and finally, six tracks were determined.

Sequentially, 22 substages of 114 early-stage ESCC patients were used as the pseudotime to construct the entire trajectory of ESCC patients. The proteins which were gradually expressed in ESCC progression (Kruskal–Wallis test, stage 9 vs. stage 1 ratio ≥ 2, FDR < 0.05) in each track were determined by the expression trend (*K* > 0) in ESCC progression by fitting curves with ggplot2 (version 3.3.0) in R (version 3.5.1), which were applied to the pathways enrichment to determine the dominant pathways of 6 tracks. Thus, in Fig. 6b, the dominant pathway of each track was defined based on the enrichment of the increased proteins during the carcinogenesis of ESCC, which was differentially expressed among the substages in ESCC progression (Kruskal–Wallis test, stage 9 vs. stage 1 ratio ≥ 2, FDR < 0.05).

### Kinase activity prediction and phosphopeptide analysis

The phosphoproteome data of 145 ESCC samples were searched against the database with MaxQuant. The phosphorylation of S or T or Y was set as variable modification, in which three mis-cleavages were allowed, with a minimum Andromeda score of 40 for spectra matches. The ratios of identified phosphorylation sites of all samples were used to estimate the kinase activities by KSEA algorithm^94^. The information on kinase-substrate relationships was obtained from publicly available databases, including PhosphoSite^95^, Phospho.ELM^96^ and PhosphoPOINT^97^. The information on substrate motifs was obtained either from the literature^98^ or from an analysis of the KSEA dataset with Motif (sP)^99^. PGK1 S203 was the only phosphosite, which was frequently detected (125/145) in ESCC progression. The motif (sP) was then matched to Human Protein Reference Database (http://hprd.org/PhosphoMotif_finder) and the kinase-substrate-motif network analysis was referenced to PhosphoSitePlus (PSP, https://www.phosphosite.org/homeAction)^100^ and NetworKIN 3.0^101^. Statistical analysis was performed in R (version 3.5.1) with the Kruskal–Wallis test.

In our cohort, we adjusted the abundance of phosphoproteins by the total protein counterpart abundance, which has been applied in previously published studies^26^. In addition, the phosphosites shown in the quantitative analysis were identified in ≥30% of samples, which has been applied in previously published studies^81,88^.

### The samples used in the statistical comparison

In the analysis of proteogenomic profiling in ESCC progression (Fig. 1 and Supplementary Figs. 1 and 2), a total of 102 samples for WES were used to explore the correlations between the two mutations. As for the analysis of the protein and phosphosite identifications on the basis of the (sub)stages/phases in ESCC progression, a total of 786 samples for proteomic profiling and 145 samples for phosphoproteomic profiling in the main cohort, and 256 samples for proteomic profiling in the validation cohort were applied.

In the analysis of the molecular characterization of alcohol drinking habit-associated signatures (SBS16 and APOBEC) (Fig. 2 and Supplementary Fig. 3), a total of overlapped samples (*n* = 102) for proteomic profiling and WES were employed to investigate the correlation (1) between signatures (SBS16 and APOBEC) and the mutations, (2) between the signatures (APOBEC and DNA repair), (3) between APOBEC signature and the clinic features of ESCC patients (e.g., drinking/smoking habits), and analyze the protein levels of the DEPs in two groups comparison (e.g., Mutation group vs. WT group, APOBEC group vs. non-APOBEC group). As for the analysis of the phosphoprotein levels of the DEPs of the phases in ESCC progression, a total of 145 samples for phosphoproteomic profiling in the main cohort were used.

In the analysis of impacts of the chromosome 3q gain, *TP53* and *MACF1* mutations in ESCC progression (Fig. 3 and Supplementary Fig. 4), the overlapped samples (*n* = 102) for proteomic profiling and WES were used to analyze the correlation (1) between mutational characteristics (e.g., chr3q gain, *MACF1* mutation, *TP63/PIK3CA/SOX2* amplifications) and the three phases in ESCC progression, (2) between *TP53* mutation and the features of ESCC patients (e.g., gender, age), and to explore the impacts of mutations on their counterpart protein levels. A total of 786 samples for proteomic profiling and 145 samples for phosphoproteomic profiling in the main cohort were applied to analyze the DEPs in the three phases of ESCC progression.

In the analysis of substage-based carcinogenesis path in ESCC progression in a time-resolved mode at the multi-omics level (Fig. 4 and Supplementary Fig. 5), the overlapped samples (*n* = 102) for proteomic profiling and WES were used to explore the correlation between mutations and the stages/phases in ESCC progression. A total of 786 samples for proteomic profiling and 145 samples for phosphoproteomic profiling in the main cohort, and 256 samples for proteomic profiling in the validation cohort were applied to investigate the protein and phosphoprotein levels of the phases/stages in ESCC progression.

In the analysis of distinctive proteomic characterization proteomic clusters which were associated with the phases in ESCC progression (Fig. 5 and Supplementary Fig. 6), the overlapped samples (*n* = 102) for proteomic profiling and WES were employed to explore the correlation between the two proteomic clusters and the (sub)stage/phases in ESCC progression, and the ESCC patients (e.g., age), and analyze the protein levels of the DEPs between the mutation group and WT group. A total of 786 samples for proteomic profiling and 145 samples for phosphoproteomic profiling in the main cohort, and 256 samples for proteomic profiling in the validation cohort were applied to investigate the protein levels and phosphoprotein levels of the DEPs in the (sub)stages in ESCC progression.

In the analysis of the personalized lineages of early-stage ESCC patients, which were closely associated with various clinical features (Fig. 6 and Supplementary Fig. 7), 68 early-stage ESCC samples from 32 cases for WES were employed to explore the correlation (1) between mutations and tracks, (2) between tracks and the features of ESCC patients. A total of 746 early-stage ESCC samples for proteomic profiling in the main cohort and 256 samples for proteomic profiling in the validation cohort were used to explore the characteristics of six tracks of ESCC.

In the analysis of the validation of PGK1 (S203) promoting ESCC progression, which was a key enzyme in glycolysis (Figs. 7 and 8 and Supplementary Fig. 8), 786 samples for proteomic profiling and 145 samples for phosphoproteomic profiling were used to differential expression of PGK1 in the stages in ESCC progression, and 102 samples overlapped for proteomic profiling and WES were used to explore the impacts of SCNA of *CDK2* on PGK1 and other kinases which were associated with the motif of PGK1 (S203). Twenty-four and fourteen samples were used for the comparisons of metabolite levels in Supplementary Fig. 8c and Supplementary Fig. 8d, respectively. Seventy-two samples were used for the comparisons of PDH activity in Fig. 8d at the cell level. Twenty-four and sixteen samples were used for comparisons in seahorse assay in Fig. 8e and Fig. 8f, respectively. Three hundred and twenty samples were used for the comparisons of cell proliferation assay exploring the impacts of the overexpression/knockdown of PGK1 and ERK2 in Fig. 8g and Supplementary Fig. 8f, and two hundred samples were used for the comparisons of cell proliferation assay exploring the impacts of the overexpression/knockdown of other enzymes (e.g., GAPDH and PGM1) in glycolysis PGK1 and ERK2 in Supplementary Fig. 8h. Thirty samples were used for the comparisons of the PGK1 inhibition effects in Fig. 8h, and twenty samples were used for the comparisons of the metabolites’ inhibition effects in Supplementary Fig. 8j. As in the mouse xenograft assay, one hundred and thirty mouse samples were used for the comparisons of tumor weight in Fig. 8i. Forty-eight samples were used for the comparisons of PGK1 expression in Supplementary Fig. 8m.

### Immunohistochemistry (IHC) analysis

To detect the expression of PGK1 in the tissue by IHC staining, 3-μm-thick sections from each FFPE tissue block were de-waxed with xylene and rehydrated through a graded series of ethanol, prepared by Zhongshan Hospital, Fudan University.

Total PGK1 immunostaining was performed on representative samples from normal to progressive ESCC. The IHC assay using PGK1 rabbit antibody (Wuhan Fine Biotech Co., Ltd, Catalog: FNab06354, dilution 1:200) was performed with Ventana iView DAB Detection Kit on a BenchMark XT automated staining system (Ventana Medical Systems, Tucson, AZ). Normal IgG from the same species of primary antibody diluted to match the concentration of the primary antibody was used as the negative control. For PGK1 negative cases, the experiments were repeated on the whole section in order to exclude heterogeneity. For assessment of staining, slides were scanned with the ScanScope System (Aperio, CA) and viewed with ImageScope (Aperio).

### Metabolite quantification

The pyruvate, lactate, citrate, succinate, fumarate, and glycine levels were measured using NMR spectra, which were also applied in other studies^102^. 3-phosphoglycerate and serine levels were measured using LC-MS/MS. Briefly, ~1 × 10^7^ cells were treated with a cold aqueous methanol solution (80% v/v) to stop cell metabolism quickly. Samples were then centrifuged for 15 min at 15,000 × g and 4 °C, after which the supernatants were collected. The supernatants were then lyophilized and reconstituted in 500 μL methanol/water (10:90 v/v). The separated metabolites were fractioned by using HPLC employing an LC-20AB pump (Shimadzu, Kyoto, Japan) and the Luna NH2 column (P/N 00B-4378-B0; 5 μm, 50 × 2.0 mm; Phenomenex, Torrance, CA). The mobile phase comprised eluent A (0.77 g NH_4_OAc, 1.25 mL NH_4_OH, 25 mL ACN, and 300 µL acetic acid [HAc] dissolved in 500 mL water) and eluent B (ACN). The elution program was as follows, 0.1 min, 85% B; 3 min, 30% B; 12 min, 2% B; 15 min, 2% B; and 16–28 min, 85% B. The flow rate of the pump was 0.3 mL/min, and the mass spectrometer used was the 4000 QTRAP system (AB Sciex, Framingham, MA) operated in multiple reaction monitoring mode. The MS parameters were electrospray voltage, 5 kV; gas 1, 30 kPa; gas 2, 30 kPa; curtain gas, 25 kPa; and temperature, 500 °C. The ions monitored for 3-phosphoglycerate and serine were at 185-79 and 106-60, respectively.

### Seahorse assays

The Mitochondrial respiration OCR and ECAR were measured by a Seahorse XF96 Extracellular Flux Analyzer (Agilent Technologies, CA, USA), using a Cell Mito Stress Test Kit (103015, Agilent Technologies) and a Glycolysis Stress Test Kit (103020, Agilent Technologies), respectively. TE-8 cells were seeded into XF96 Cell Culture Microplates (101085, Agilent Technologies) at the density of 5000 cells/well in an assay media supplemented with 1 mM pyruvate and 80 uL of RPMI, centrifuged for 10 min, and allowed to adhere to plate overnight. Then, the culture medium was replaced with phenol red-free assay solution and cells were equilibrated for 1 h without CO_2_ immediately before the extracellular flux (XF) assay. For the mitochondrial stress test, oligomycin, carbonyl cyanide 4-(triflfluoromethoxy) phenylhydrazone and rotenone/antimycin A, respectively, were added according to the manufacturer’s instructions and protocols. The glycolytic rate assay was performed in XF Base Media without phenol red, oligomycin and 2-deoxy-Dglucose were added in proper order.

### Cell lines and cell culture

The following ESCC cell lines were used in this assay: Human ECA109 cells (ATCC, Catalog: GCC-OE0002CS, RRID: CVCL_6898), Human KYSE150 cells (ATCC, Catalog: GCC-OE0004CS, RRID: CVCL_QW54), Human KYSE70 cells (YaJi Biological, Catalog: YS1331C, RRID: CVCL_1356), Human TE-8 cells (YaJi Biological, Catalog: YS2958C, RRID: CVCL_1766). All cells were cultivated in RPMI-1640 medium (HyClone, Logan, UT, USA) supplemented with 10% fetal bovine serum (HyClone, Logan, UT, USA), and incubated at 37 °C in 5% CO_2_.

### Gene overexpression and knockdown

For transient gene overexpression, the whole-length cDNA of *PGK1*, *GAPDH* and *PGM1* were cloned into pcDNA3.1 (b)-Flag vector between the NheI and EcoRI sites. The whole-length cDNA of *ERK2* was cloned into pcDNA3.1(b)-Myc vector between the NheI and EcoRI sites, whereas the plasmid PGK1-S203A-Flag was generated by site-directed mutagenesis using the Muta-nBEST kit (TaKaRa, Kyoto, Japan, Catalog: R401) according to the manufacturer’s instructions. Then plasmids were transfected into KYSE150 cells, KYSE70 cells, ECA109 cells, and TE-8 cells using Lipofectamine 3000 (Invitrogen, Carlsbad, CA, USA) according to the manufacturer’s instructions. For stable overexpression of *PGK1*, the whole-length cDNA of *PGK1* was cloned into vector pBABE puro between the BamHI and EcoRI sites; then, the plasmids were co-transfected with pCMV-VSV-G and pCMV-Gag-Pol plasmids into KYSE150 cells, KYSE70 cells, ECA109 cells, and TE-8 cells using the calcium phosphate method. For stable knockdown of *PGK1* and *ERK2*, pMKO.1-shRNA plasmids encoding specific shRNAs targeting human PGK1 (5′–CTGACAAGTTTGATGAGAATG–3′) and human ERK2 (5′–CAAAGTTCGAGTAGCTATCAA–3′) were transfected, together with pCMV-VSV-G and pCMV-Gag-Pol plasmids, into one HEK293T packaging cell line using the calcium phosphate method and the virus supernatants were collected from the medium for the subsequent infection of KYSE150 cells, KYSE70 cells, ECA109 cells, and TE-8 cells.

### Immunoprecipitation

For immunoprecipitation of the FLAG-tagged proteins, cells were lysed with 0.1% NP-40 buffer containing 50 mM Tris-HCl (pH 7.5), 150 mM NaCl, 0.1% NONIDET P-40, 1 μg/mL aprotinin, 1 μg/mL leupeptin, 1 μg/mL pepstatin, and 1 mM PMSF. The whole-cell lysates were incubated with monoclonal anti-Flag antibody-conjugated M2 agarose beads (Sigma) for 4 h at 4 °C. The bound proteins were triple-washed with 0.1% NP-40 buffer.

### Western blot analysis

Standard procedures were followed for western blot analysis. Primary antibodies used in this study include anti-PGK1 antibody (Wuhan Fine Biotech Co., Ltd., China, Catalog: FNab06354, dilution 1:1000), Anti-β-actin (Genscript, Piscataway, NJ, USA, Catalog: A00702, dilution 1:10,000), Anti-p-Ser (Cell Signaling Technology, Danvers, MA, USA, Catalog:9606, dilution 1:4000), Anti-phospho-Threonine (Cell Signaling Technology, Danvers, MA, USA, Catalog: 9386, dilution 1:1000), Anti-phospho-Tyrosine (Cell Signaling Technology, Danvers, MA, USA, Catalog: 9411, dilution 1:2000), Anti-COX IV (Cell Signaling Technology, Danvers, MA, USA, Catalog: 4580, dilution 1:1000), Anti-GAPDH (Cell Signaling Technology, Danvers, MA, USA, Catalog: 85925, dilution 1:10,000), Anti-Thr-338 PDHK1 (Signalway Antibody, Nanjing, China, Catalog: C11596, dilution 1:500), Anti-PDHK1 (Cell Signaling Technology, Danvers, MA, USA, Catalog: 3820, dilution 1:1000), Anti-ERK2 (Cell Signaling Technology, Danvers, MA, USA, Catalog: 9108, dilution 1:1000), Anti-Flag (Abmart, Shanghai, China, Catalog: M20008, dilution 1:5000), Anti-PGM1 (Cell Signaling Technology, Danvers, MA, USA, Catalog: 12098, dilution 1:1000), and Anti-PHGDH (Cell Signaling Technology, Danvers, MA, USA, Catalog: 66350, dilution 1:1000). Western blot signals were obtained by detecting chemiluminescence by using a Typhoon FLA 9500 biomolecular imager (GE Healthcare).

### PGK1 purification and enzymatic assay

Flag-tagged PGK1 protein immuno-precipitated from HEK293T cells was eluted with Flag peptide buffer. The eluent was further purified and concentrated using an Amicon Ultra Centrifugal Filter (10 kDa molecular weight cutoff, Millipore) in a buffer containing 50 mM Tris-HCl pH 7.5, 100 mM KCl, 5 mM MgCl_2_ and 5% glycerol. PGK1 activity was measured using purified Flag-tagged PGK1 (0.2 μg/mL) mixed with DMSO or different concentrations of gemcitabine in the reaction buffer containing 50 mM Tris-HCl (pH 7.6), 8 mM MgCl_2_, 4 mM ATP, 0.2 mM NADH, 12 mM 3-phosphoglycerate, and 8 U of GAPDH at 25 °C. The change in absorbance at 340 nm owing to the decrease of NADH was measured.

### PDH activity assay

The assays were carried out using PDH Enzyme Activity Microplate Assay Kit (Abcam, Catalog: ab109902) according to the manufacturer’s guidance. The total intracellular PDH activities of KYSE150 cells, KYSE70 cells, ECA109 cells, and TE-8 cells were normalized with the protein expression levels of COX IV.

### Cell proliferation assay

Cell proliferation was assessed using the Cell Counting Kit-8 (Dojindo Molecular Technologies, Inc, Kumamoto, Japan, Catalog: CK04). In brief, cells were seeded in a 96-well plate at 4 × 10^3^ cells per well and allowed to adhere. Cell Counting Kit-8 solution (10 μL) was added to each well, and the cells were cultured in 5% CO_2_ at 37 °C for 2 h. Cell proliferation was determined by measuring the absorbance at 450 nm.

### Mouse xenograft assay

Five-week-old male Balb/C nude mice were obtained (Shanghai SLAC Laboratory Animal Co., Ltd, Shanghai, China) for in vivo xenografts. Mice were housed in polycarbonate cages, and provided free access to food and water with a 12-h light:dark cycle. ESCC cells (~1 × 10^7^) were subcutaneously injected into nude mice. When the tumor volumes reached approximately 100 mm^3^, PGK1-OE tumor-bearing mice were randomly separated into two groups (*n* = 10 per group) as follows: PGK1-OE group and PGK1-OE-inhibitor (gemcitabine (Sigma-Aldrich, Catalog: G6423, 6 mg/kg) group. Mice in the group of PGK1-OE-inhibitor were injected intravenously every other day for eight times; while the mice in the other group were injected saline. Tumors were harvested and weighed after 30 days post injection. All experimental procedures involving animals in this study were approved by the Fudan University Institutional Animal Care and Use Committee and were conducted in accordance with the National Institutes of Health Guidelines for the Care and Use of Laboratory Animals. The maximal tumor burden permitted by the committee is 2000 mm^3^.

### Statistics and reproducibility

Statistical details of experiments and analyses can be found in the (supplementary)/figure legends and supplementary datasets in this text. Standard statistical tests were used to analyze the clinical data, including but not limited to Wilcoxon signed-rank test, Fisher’s exact test, Kruskal–Wallis test. Specifically, the statistical significance of differences between the two groups was calculated with the Wilcoxon rank-sum test and Student’s *t*-test; for more than two groups’ comparison, Kruskal–Wallis test was used. When exploring the association of different groups with clinical variables, Fisher’s exact test and Wilcoxon rank-sum test were used for categorical variables and continuous variables, respectively. As for the correlation analysis between two proteins/phosphoproteins, Pearson’s correlation of correlation coefficients was used. For the correlation analysis among the stages in ESCC progression and different HEK293T cell samples, Spearman’s correlation of correlation coefficients was used. Kaplan–Meier plots (two-sided log-rank test) were used to describe the OS. To validate the findings in this study, each experiment was repeated at least three times independently. In this study, all analyses were performed in R (version 4.0.2) and GraphPad Prism (Version 9), and all statistical tests were two-sided, and statistical significance was considered when *p* value <0.05, which was adjusted using the BH procedure. Data in the boxplot were presented as median (central line), upper and lower quartiles (box limits), 1.5× interquartile range (whiskers).

### Reporting summary

Further information on research design is available in the Nature Portfolio Reporting Summary linked to this article.

## Supplementary information

Supplementary Information

Description of Additional Supplementary Files

Supplementary Data 1

Supplementary Data 2

Supplementary Data 3

Supplementary Data 4

Supplementary Data 5

Supplementary Data 6

Supplementary Data 7

Reporting Summary

## Data Availability

The proteome and phosphoproteome raw datasets generalized in this study have been deposited to the ProteomeXchange Consortium (dataset identifier: PXD038961) via the iProX partner repository (https://www.iprox.cn/)^103^ under Project ID IPX0002178000. The VCF files of the WES data files were deposited to the European Genome-Phenome Archive (EGA) associated with the study EGAS00001006126 under project ID EGAD00001008672. Data are available upon request through EGA without any restrictions, and will be available permanently. The raw WES data are available in the GSA^104^ (Genome Sequence Archive, https://ngdc.cncb.ac.cn/gsa-human/) under restricted access HRA004153 for data privacy laws related to patient consent for data sharing, access can be obtained by the Request Data steps in GSA database website or contacting the corresponding author. The approximate response time for accession requests is about 2 weeks. Once access has been granted, the data will be available to download for 3 months. The gene expression profiles of ESCC cell lines in the public dataset Expression 21Q2 in this study are available in the Depmap database (https://depmap.org/portal/download/?releasename=DepMap+Public+21Q2&filename=CCLE_expression.csv). The remaining data are available within the article, Supplementary Information, or Source Data file. Source Data are provided with this paper.

## Source data

Source Data

## Code availability

No special code was used in this study, and codes for all figures in the study are available for research purposes from the corresponding authors on request.
