# Supplementary material for: Integrative proteogenomic characterization of early esophageal cancer
Source: Nat Commun. 2023 Mar 25;14:1666. doi: 10.1038/s41467-023-37440-w (PMC10039899; doi:10.1038/s41467-023-37440-w)
Supplement: Supplementary file 1 — Supplementary Information [file 41467_2023_37440_MOESM1_ESM.pdf]

Supplementary Fig. 1

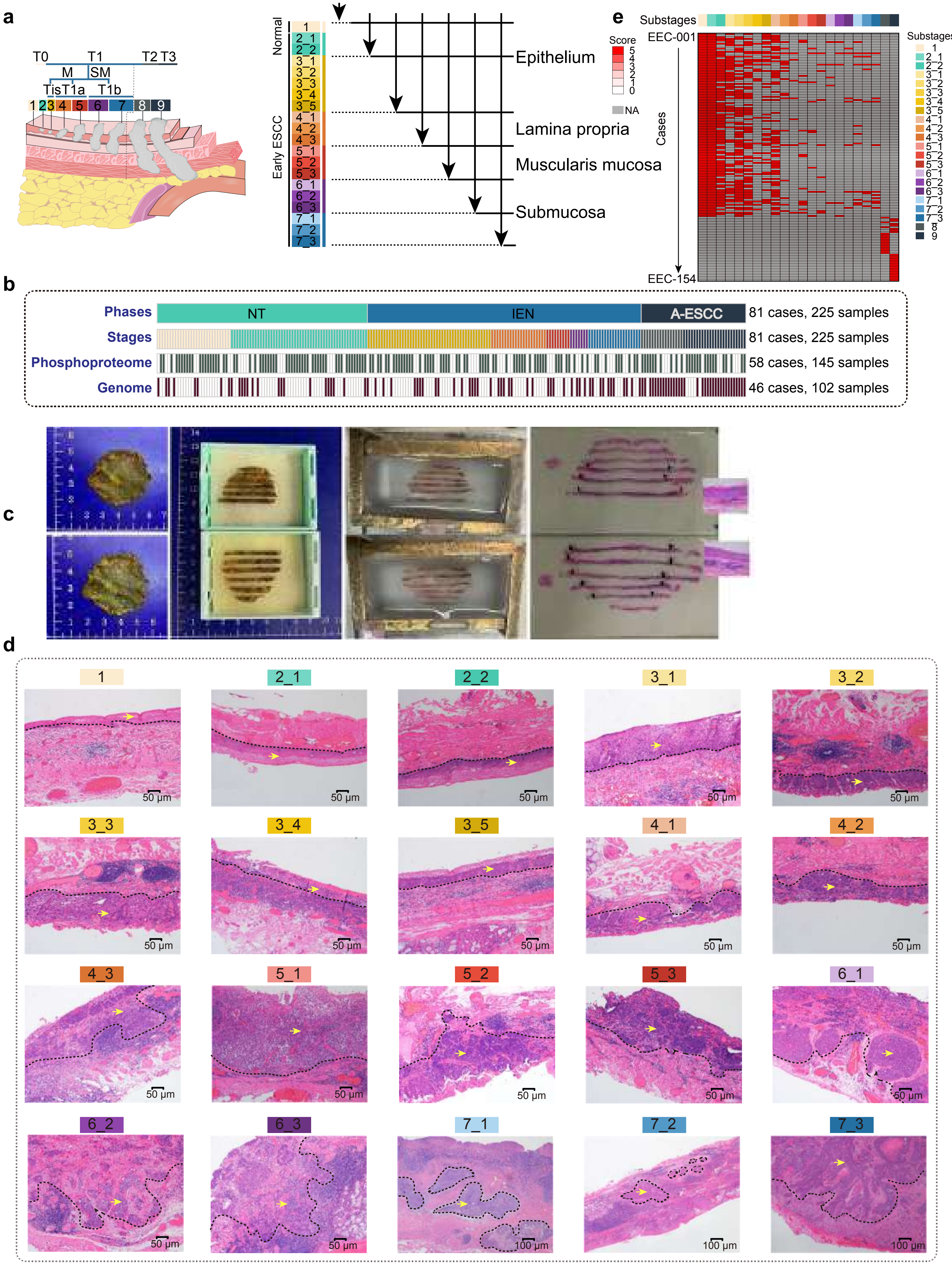

Supplementary Fig. 1 H&E stained on early ESCCs. (a) Invasion layer in ESCC progression. T0 – T1 indicated the early ESCC stages, ranging from substage 1 (normal tissue stage) to substage 7_3 (submucosa stage). T2 – T3 represent the advanced ESCC stages, including stages 8 (T2 stage) and 9 (T3 stage). Diversity sign is marked the distinctive invasion layer on the right. **(b)** The links of phosphoproteome data to genome data. The square directs to a subset of patient samples used for phosphoproteome or for WES (n = 102). NT phase: non-tumor phase. IEN phase: intraepithelial neoplasia phase. A-ESCC phase: advanced-stage esophageal squamous cell carcinoma phase. **(c)** The procedure of dissection and embedding. **(d)** H&E staining analysis of different substages in early ESCC. **(e)** The normal epithelial/tumor cell purity of 786 samples in ESCC progression. Source data are provided as a Source data file.

Supplementary Fig. 2

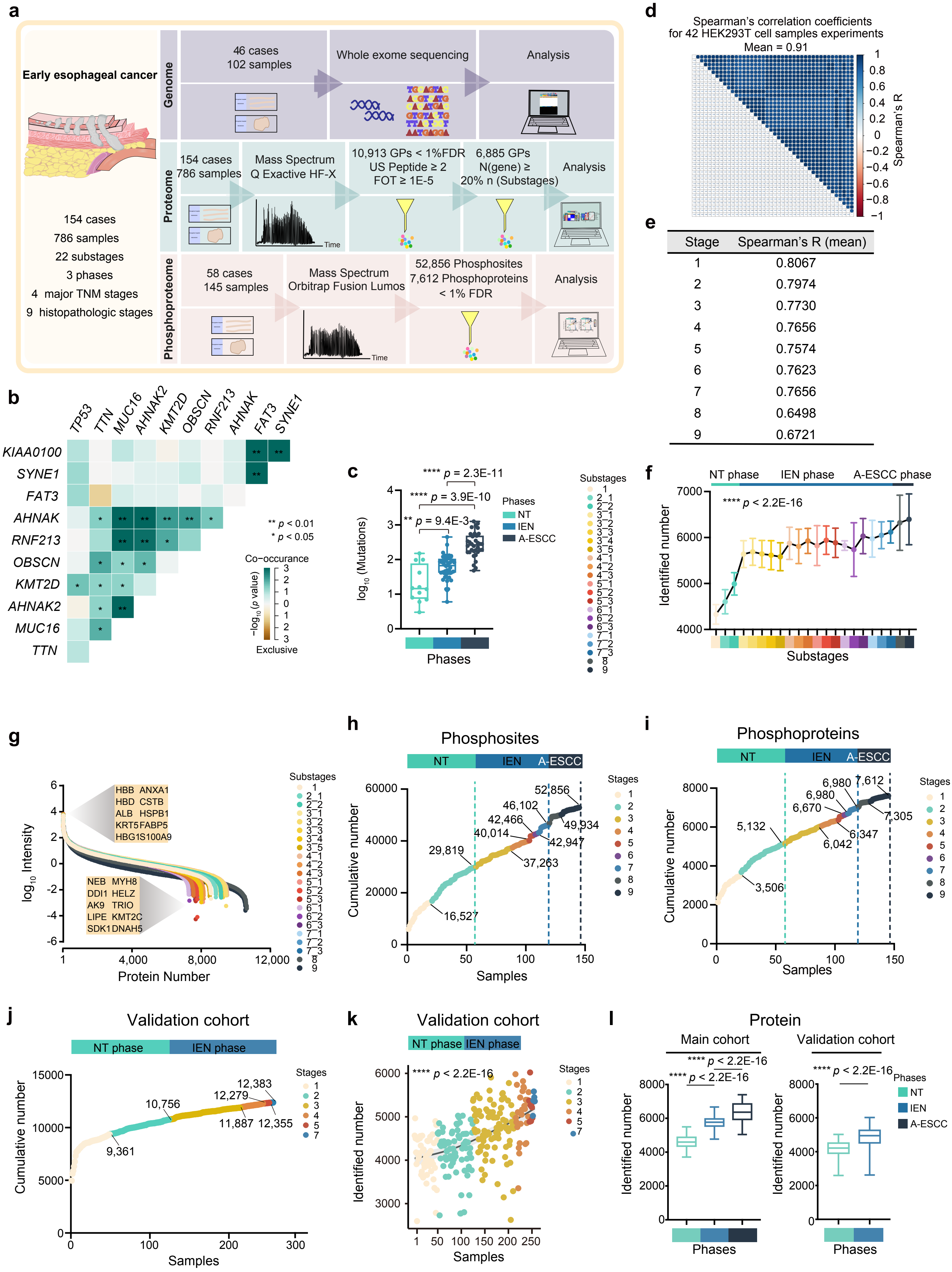

e

Stage	Spearman's R (mean)
1	0.8067
2	0.7974
3	0.7730
4	0.7656
5	0.7574
6	0.7623
7	0.7656
8	0.6498
9	0.6721

Supplementary Fig. 2 A brief workflow of multi-omics analysis and overview of the proteomic profiles of early and progressive ESCC cohorts. (a) The workflow of sampling, processing, and analysis of all ESCC samples at the multi-omics level. The 786 samples are collected from 154 ESCC patients, including those from the surgery samples (n = 40, T2 – T3) and biopsy samples after ESD (n = 746, T0–T1). **(b)** The exclusively co-mutations of the top 10 mutations in the genomics data (n = 102) of the Fudan cohort (two-sided Fisher's exact test, $p < 0.05$). **(c)** Boxplot showing the TMB in the NT, IEN, A-ESCC phases of ESCC (two-sided Wilcoxon signed-rank test). A total of 102 samples for WES are used in the analysis. ** $p = 9.4E-3$ (NT & IEN), **** $p = 3.9E-10$ (IEN & A-ESCC), **** $p = 2.3E-11$ (NT & A-ESCC). **(d)** (Spearman's) correlation analysis of 42 HEK293T cell samples as MS quality control to evaluate the robustness of label-free quantification (two-sided Spearman's correlation test). **(e)** Table chart showing the (Spearman's) correlation coefficients (mean) of stages in ESCC progression (n = 786, two-sided Spearman's correlation test). **(f)** The number of protein identifications of 22 substages in ESCC progression (Kruskal-Wallis test, **** $p < 2.2E-16$, mean ± SD). n (stage 1) = 114, n (stage 2) = 206, n (stage 3) = 259, n (stage 4) = 86, n (stage 5) = 32, n (stage 6) = 17, n (stage 7) = 32, n (stage 8) = 16, n (stage 9) = 24 biologically independent samples examined. **(g)** The dynamics of protein abundance identified in 22 substages. Proteins are quantified as normalized iBAQ value and transformed to $\log_{10}$ Intensity. The highest- and lowest- abundance proteins are shown in the box. The cumulative number of the phosphosites **(h)** and phosphoproteins **(i)** of 145 samples in ESCC progression. **(j)** The cumulative number of the proteins of 256 samples (validation cohort) in ESCC progression. **(k)** The number of the identified proteins of 256 samples (validation cohort) in ESCC progression (Kruskal-Wallis test, **** $p < 2.2E-16$). **(l)** Boxplots showing the number of the protein identifications in the phases during the carcinogenesis of ESCC in the main cohort (left, n = 786) and validation cohort (right, n = 256) (two-sided Wilcoxon rank-signed test). **** $p < 2.2E-16$, **** $p < 2.2E-16$, **** $p < 2.2E-16$ from left to right. Boxplots show median (central line), upper and lower quartiles (box limits), 1.5× interquartile range (whiskers) in panels **c**, **l**. **** $p < 1.0E-4$, *** $p < 1.0E-3$, ** $p < 0.01$, * $p < 0.05$, ns. > 0.05. Source data are provided as a Source data file.

Supplementary Fig. 3

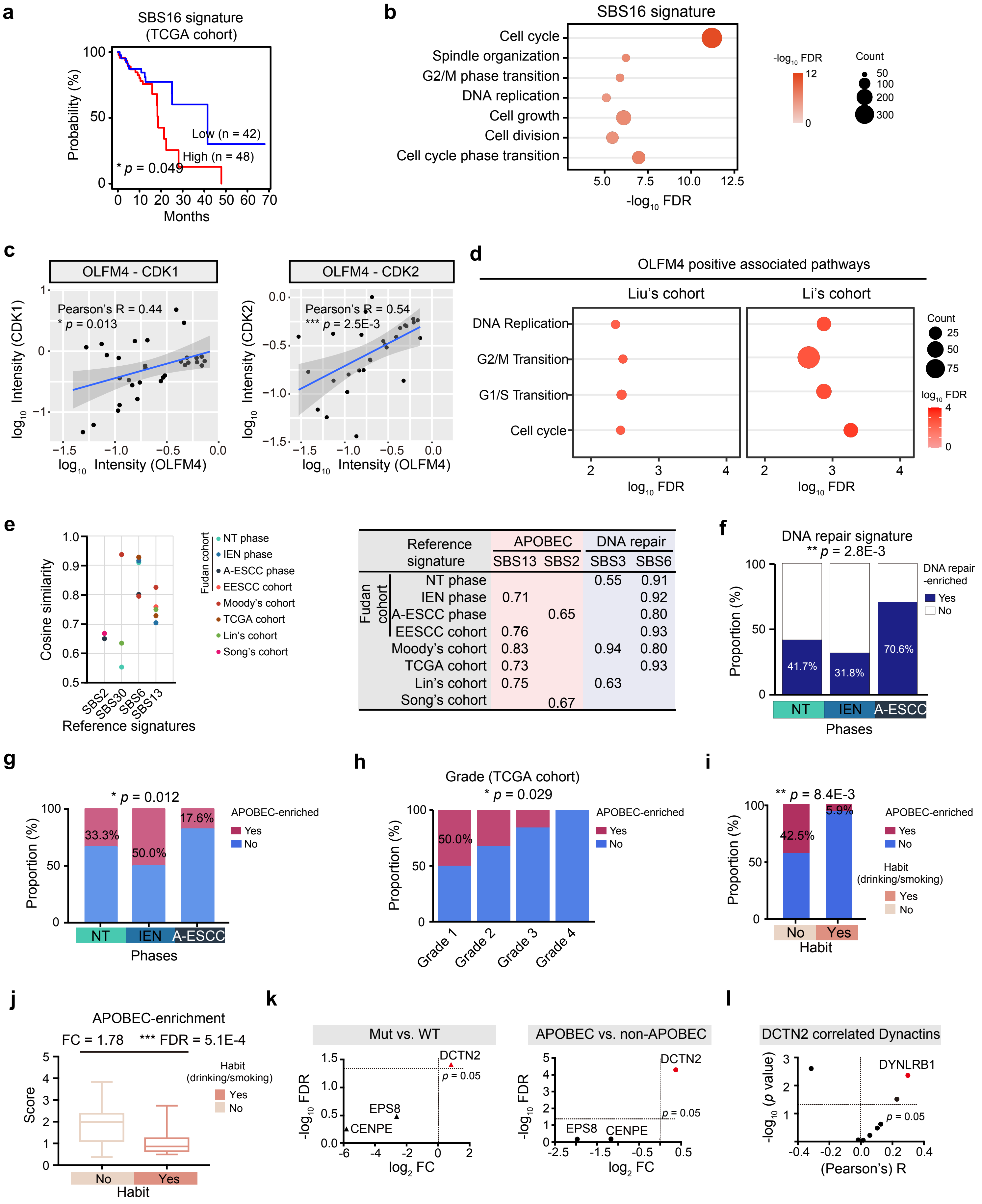

Reference signature	APOBEC SBS13	APOBEC SBS2	DNA repair SBS3	DNA repair SBS6
Fudan cohort: NT phase			0.55	0.91
Fudan cohort: IEN phase	0.71			0.92
Fudan cohort: A-ESCC phase		0.65		0.80
EESCC cohort	0.76			0.93
Moody's cohort	0.83		0.94	0.80
TCGA cohort	0.73			0.93
Lin's cohort	0.75		0.63	
Song's cohort		0.67		

Supplementary Fig. 3 The risk factor associated signature in ESCC progression. (a) Survival analysis of patients with high SBS16 signature score (two-sided log-rank test). The survival information is referred from the TCGA ESCC cohort. **(b)**The dominant pathways in SBS16 signature enrichment group. **(c)** Scatter plot showing the (Pearson's) correlation between OLFM4 and CDK1 (left), and OLFM4 and CDK2 (right) (two-sided Pearson's correlation test, mean ± SD). **(d)** The represented enrichment pathways with OLFM4 positively associated proteins in other ESCC cohorts. **(e)** The cosine similarity (left) and dominant signatures (right) of the Fudan cohort and other ESCC cohorts. **(f)** Histogram showing the proportion of DNA repair signature in the three phases of ESCC (two-sided Fisher's exact test). **(g)** Histogram showing the proportion of APOBEC signature in ESCC progression (two-sided Fisher's exact test). **(h)** Histogram showing the proportion of APOBEC signature in the four grades in ESCC progression of TCGA cohort (two-sided Fisher's exact test). **(i)** Histogram showing the proportion of APOBEC signature in ESCC patients with different habits (drinking/smoking) (two-sided Fisher's exact test). **(j)** Boxplot showing higher APOBEC enrichment score in non-smoking/drinking ESCC patients (two-sided Wilcoxon signed-rank test). Boxplots show median (central line), upper and lower quartiles (box limits), 1.5× interquartile range (whiskers). n (APOBEC enriched) = 32, n (APOBEC non-enriched) = 58 biologically independent samples examined. **(k)** Volcano analysis showing the impacts of *DCTN2/EPS8/CENPE* mutations (left) and APOBEC signature (right) on the protein-levels of DCTN2/EPS8/CENPE (two-sided Wilcoxon signed-rank test). **(l)** Volcano plot depicting the (Pearson's) correlation between DCTN2 and the dynactin families' proteins (two-sided Pearson's correlation test). **** $p < 1.0E-4$, *** $p < 1.0E-3$, ** $p < 0.01$, * $p < 0.05$, ns. > 0.05. Source data are provided as a Source data file.

Supplementary Fig. 4

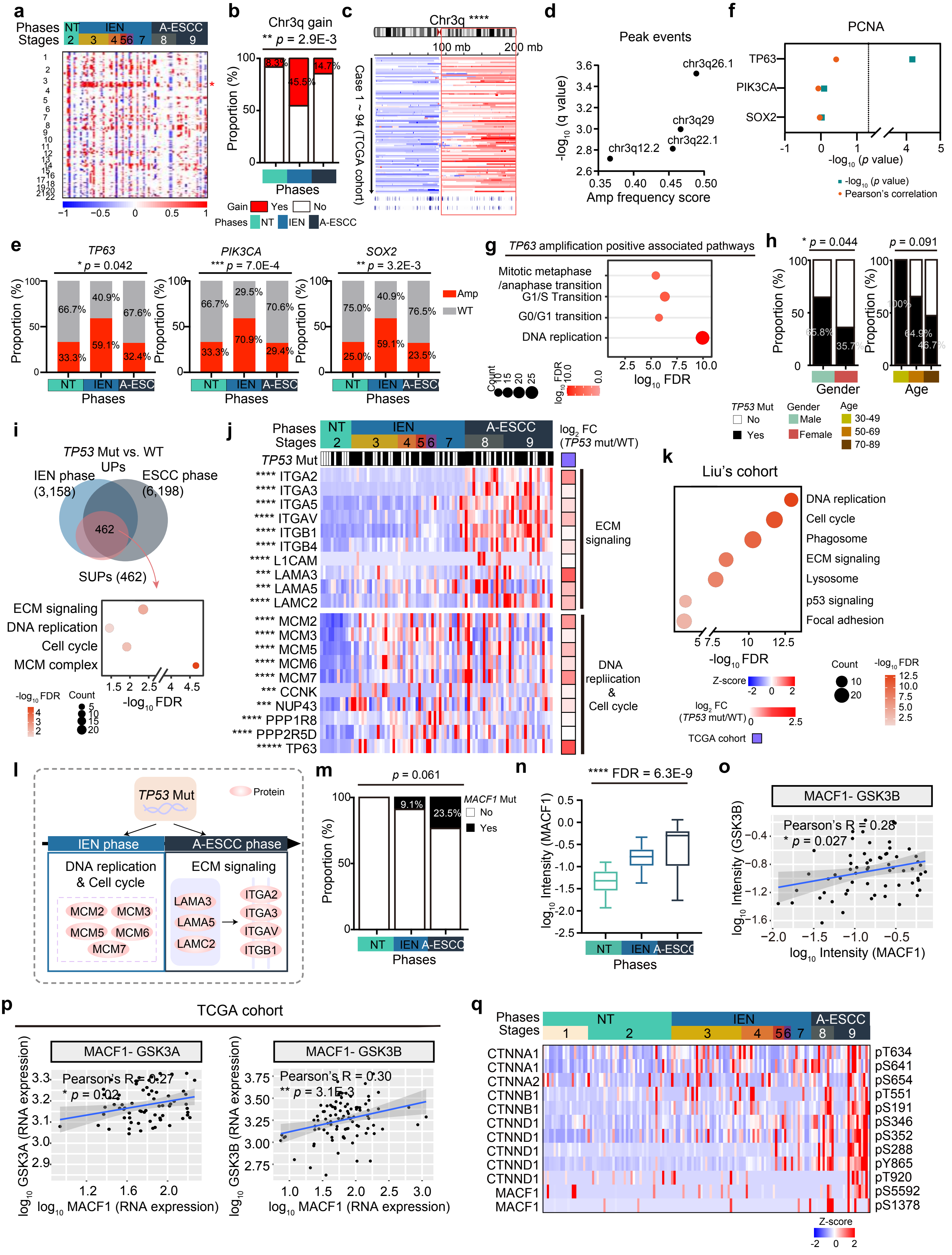

Supplementary Fig. 4 Integrative analyses of genomics, proteomics, and phosphoproteomics data in ESCC progression. (a) Profiling of absolute copy number alterations observed in the Fudan cohort. The square directs to a subset of patient samples used for WES (n = 102). (**b)** Histogram showing the proportion of the chr3q gain in the three phases of 102 samples in ESCC progression (two-sided Fisher's exact test). **(c)** Profiling of absolute copy number alterations showing the roles of chr3q gain in the TCGA ESCC cohort (two-sided Fisher's exact test, **** p < 2.2E-16). **(d)** The significant peaks events at the chr3q gain in ESCC progression. A total of 102 samples for WES are used in the analysis. **(e)** Histograms showing the proportion of the amplifications of *TP63*/*PIK3CA/SOX2* in the three phases of 102 samples in ESCC progression (two-sided Fisher's exact test). **(f)** The (Pearson's) correlation between TP63/PIK3CA/SOX2 and PCNA at the protein level (two-sided Pearson's correlation test). **(g)** The represented biological pathways associated with *TP63* amplification. **(h)** Histograms showing the proportion of *TP53* mutation in gender (left) and ages (right) in ESCC progression (two-sided Fisher's exact test). **(i)** The number of the overlapped proteins (top) enhanced by *TP53* mutation and the representative pathways (bottom) (two-sided Wilcoxon rank-signed test). Ups: the up-regulated proteins. **(j)** Heatmap showing the proteins and the associated biological pathways elevated by *TP53* mutation in ESCC progression (Kruskal-Wallis test, BH-adjusted * $p < 0.05$). The square directs to a subset of patient samples used for WES (n = 102). The fold change (*TP53* Mut vs. WT ratio) of the TCGA cohort shown in the right. **(k)** The represented pathways in the tumor tissues compared to the paired non-cancerous adjacent tissues (NATs). **(l)** A brief summary of the impacts of *TP53* mutation in ESCC progression. **(m)** Histogram showing the proportion of *MACF1* mutation in three phases in ESCC progression (n = 102 for WES) (two-sided Fisher's exact test). **(n)** Boxplot depicting the expression of MACF1 in ESCC progression at the protein level (Kruskal-Wallis test). Boxplot shows median (central line), upper and lower quartiles (box limits), 1.5× interquartile range (whiskers). The overlapped samples (n = 90) for proteomic profiling and WES were used in the analysis, in which normal tissues were not included. n (NT) = 12, n (IEN) = 44, n (A-ESCC) = 34 biologically independent samples examined. **(o)** Scatterplot showing the relationship between $\log_{10}$ GSK3B and $\log_{10}$ MACF1 expression (n = 102 for WES) at the protein level (two-sided Pearson's correlation test, mean ± SD). **(p)** Scatterplots showing the relationship between $\log_{10}$ GSK3A (left)/GSK3B (right) and $\log_{2}$ MACF1 expression at the RNA level in TCGA ESCC cohort (two-sided Pearson's correlation test, mean ± SD). **(q)** Heatmap showing the impacts of

MACF1 mutation on the expression of Wnt signaling related phosphoproteins in ESCC progression. The square directed to a subset of patient samples used for phosphoproteome. A total of 145 samples for phosphoproteomic profiling are used in the analysis. **** $p < 1.0E-4$, *** $p < 1.0E-3$, ** $p < 0.01$, * $p < 0.05$, ns. > 0.05. Source data are provided as a Source data file.

Supplementary Fig. 5

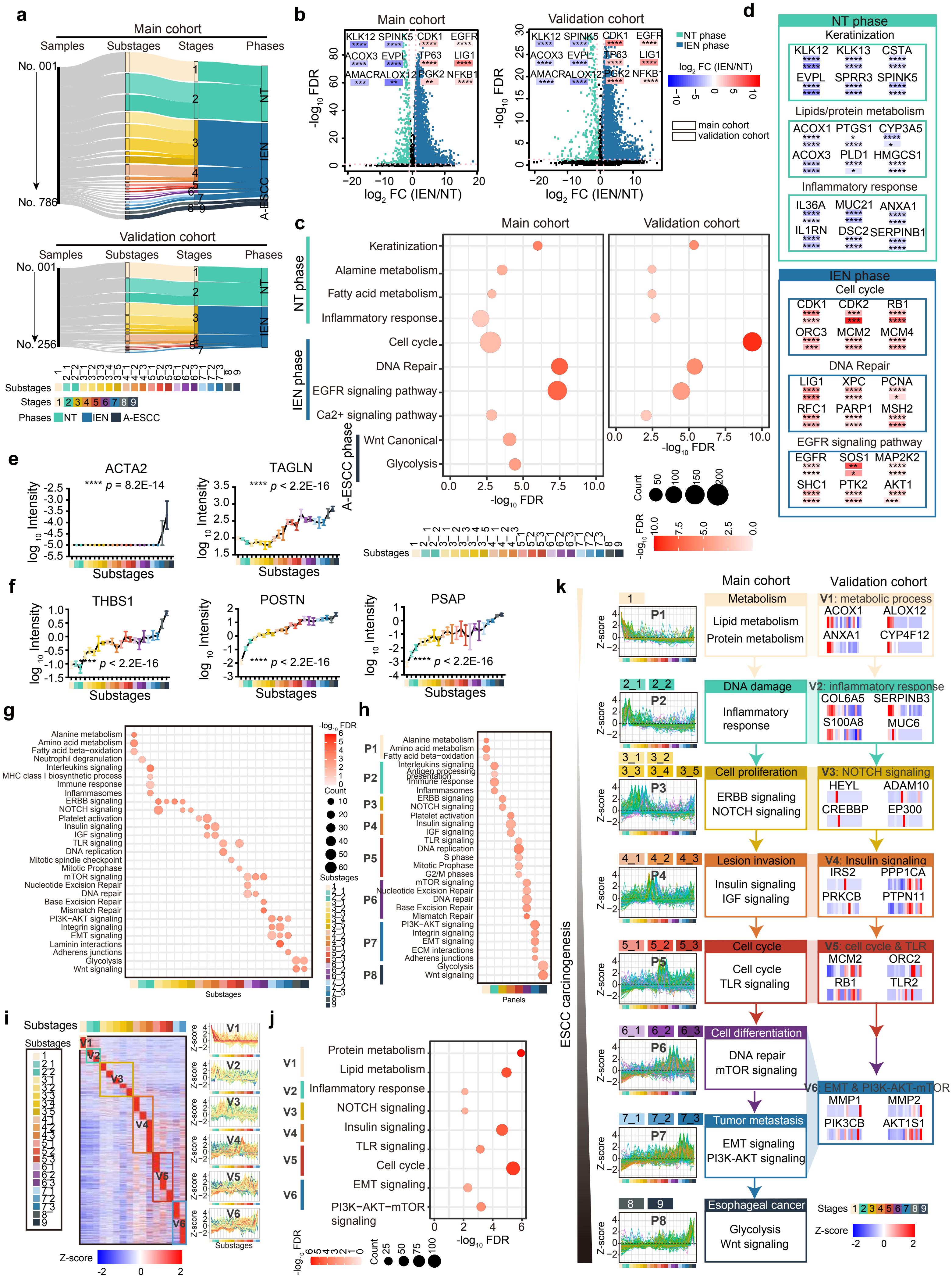

Supplementary Fig. 5 The Immune-based panels and dynamic driver pathway waves of 8 panels in ESCC progression. (a) Sankey diagram analysis of 786 and 256 samples in the main cohort (top) and validation cohort (bottom), respectively. **(b)** Volcano plot depicting the DEPs in the main cohort (left) and validation cohort (right) (two-sided Wilcoxon rank-signed test, **** $p < 1.0E-4$). **(c)** The represented biological pathways of the phases in the main cohort (left, n = 786) and validation cohort (right, n = 256) (two-sided Wilcoxon rank-signed test). Biological pathways are analyzed from the Reactome database**.** **(d)** The represented pathways and associated proteins in the NT phase (top) and IEN phase (bottom) in the main cohort (n = 786) and validation cohort (n = 256) (two-sided Wilcoxon rank-signed test, * $p < 0.05$). Column showing the expression of ESCC biomarkers from other ESCC studies **(e)** (*Yazdian-Robati* et al.) and **(f)** (*Pawar* et al.) in ESCC progression (Kruskal-Wallis test, mean ± SEM, **** $p < 2.2E-16$). Twenty-two substages and $\log_{10}$ Intensity were indicated on x and y axis, respectively. n (stage 1) = 114, n (stage 2) = 206, n (stage 3) = 259, n (stage 4) = 86, n (stage 5) = 32, n (stage 6) = 17, n (stage 7) = 32, n (stage 8) = 16, n (stage 9) = 24 biologically independent samples examined. **(g)** The represented pathways of 22 substages in ESCC progression. **(h)** The dominant pathways of 8 panels in ESCC progression. A total of 786 samples for proteomic profiling were used in the analysis. **(i)** Substage-based supervised clustering (left) and K-means (right) analysis of 6 proteomic patterns in the validation cohort (n = 256). **(j)** The represented biological pathways of 6 proteomic patterns in the validation cohort (n = 256). **(k)** A carcinogenesis path during ESCC progression in the main cohort (left) and validation cohort (right) The results of the k-means analysis were shown. **** $p < 1.0E-4$, *** $p < 1.0E-3$, ** $p < 0.01$, * $p < 0.05$, ns. > 0.05. Source data are provided as a Source data file.

Supplementary Fig. 6

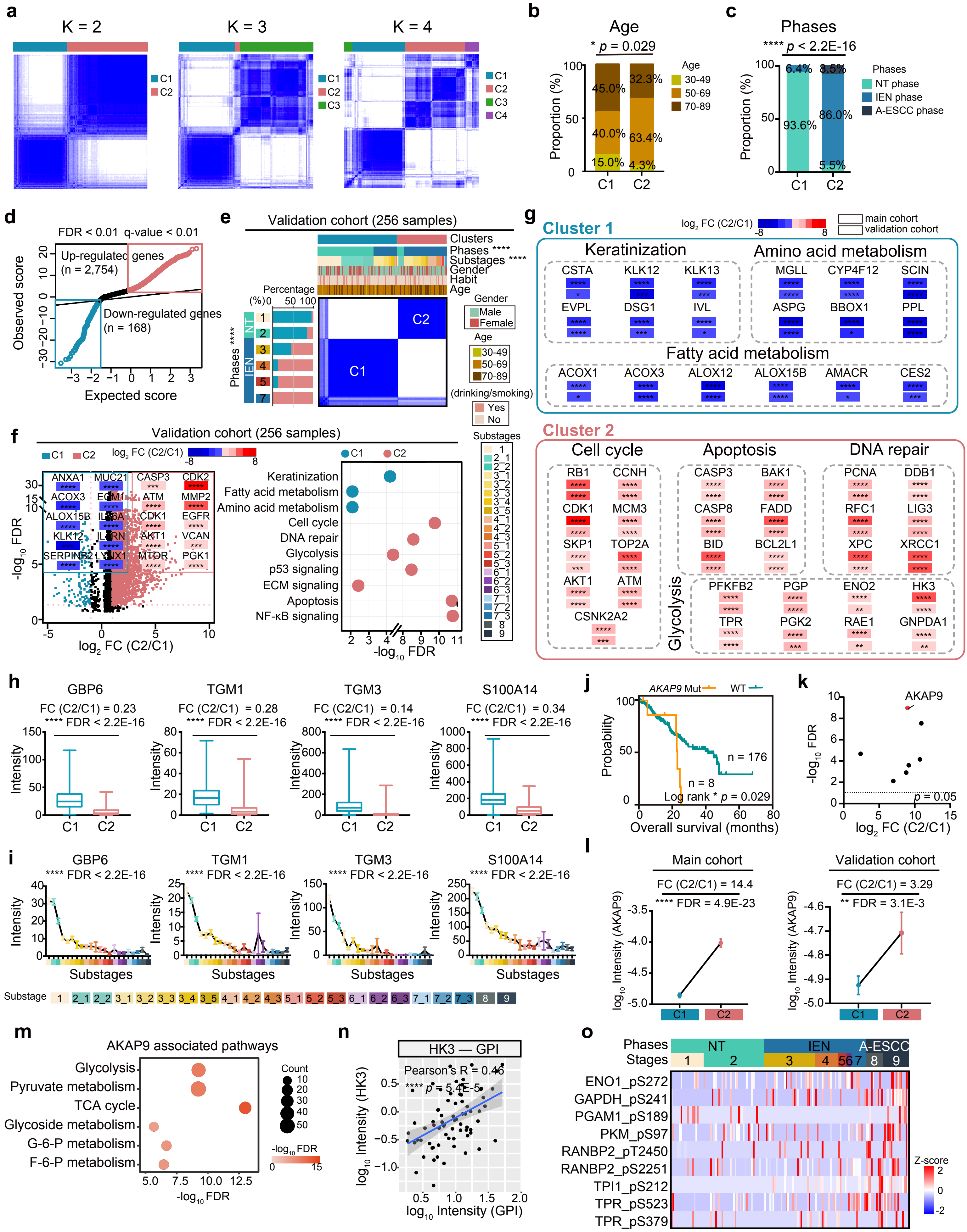

Supplementary Fig. 6 Proteomic characteristics of two clusters and the impacts of *AKAP9* mutation. **(a)** Heatmap showing consensus matrix with **κ** = 2, 3, and 4. The input was the quantile-normalized iBAQ intensity (FOT) matrix of variable proteomics data (n = 6,885). **(b)** Histogram showing the difference between the two clusters in different ages of ESCC patients (two-sided Fisher's exact test). **(c)** Histogram showing the proportion of the phases in the two clusters (two-sided Fisher's exact test, **** p < 2.2E-16). **(d)** Scatterplot displaying the samfit of the filtered up-regulated genes (red) and down-regulated genes (green) of 786 sample in ESCC progression (two-sided Student's t-test, mean ± SD). **(e)** Consensus clustering of 256 samples in the validation cohort (two-sided Fisher's exact test, **** p < 2.2E-16 for phases and substages). **(f)** Volcano analysis of DEPs (left) and the represented biological pathways (right) in the two clusters in the validation cohort (two-sided Student's t-test, **** p < 1.0E-4). Biological pathways are analyzed from the GO/KEGG database. **(g)** Genebox showing the DEPs and the representative pathways in the C1 (top) and C2 (bottom) in the main cohort and validation cohort (two-sided Student's t-test, * p < 0.05). **(h)** Boxplots showing the expression of specific molecules of esophageal tissue in the two clusters (two-sided Student's t-test, **** p < 2.2E-16). Boxplots show median (central line), upper and lower quartiles (box limits), 1.5× interquartile range (whiskers). n (C1) = 314, n (C2) = 472 biologically independent samples examined. **(i)** Column showing the expression of specific molecules of esophageal tissue in ESCC progression (Kruskal-Wallis test, mean ± SEM, **** p < 2.2E-16). n (stage 1) = 114, n (stage 2) = 206, n (stage 3) = 259, n (stage 4) = 86, n (stage 5) = 32, n (stage 6) = 17, n (stage 7) = 32, n (stage 8) = 16, n (stage 9) = 24 biologically independent samples examined. **(j)** Survival analysis of patients with *AKAP9* mutation versus WT comparison of ESCC (two-sided log-rank test). The survival information was referred the TCGA ESCC cohort. **(k)** Volcano analysis showing the impacts of the C2 significant mutations on their counterpart protein levels (n = 102 for WES, two-sided Wilcoxon signed-rank test). **(l)** Column showing the expression of AKAP9 in the two clusters in the main cohort (left) and validation cohort (right) (two-sided log-rank test, mean ± SEM). Main cohort: n (C1) = 314, n (C2) = 472 biologically independent samples examined. Validation cohort: n (C1) = 158, n (C2) = 98 biologically independent samples examined. **(m)** Represented pathways enrichment of proteins which were positive correlated with AKAP9 in the validation cohort (n = 256). **(n)** Scatterplot showing the relationships between $\log_{10}$ HK3 and $\log_{10}$ GPI expression at the protein level (two-sided Pearson's correlation test). **(o)** Heatmap showing the phosphorylation of the phosphoproteins in the

dominant pathways regulated by *AKAP9* mutation in ESCC progression (Kruskal-Wallis test). The square directs to a subset of patient samples used for phosphoproteome (n = 145). **** $p < 1.0E-4$, *** $p < 1.0E-3$, ** $p < 0.01$, * $p < 0.05$.

Supplementary Fig. 7

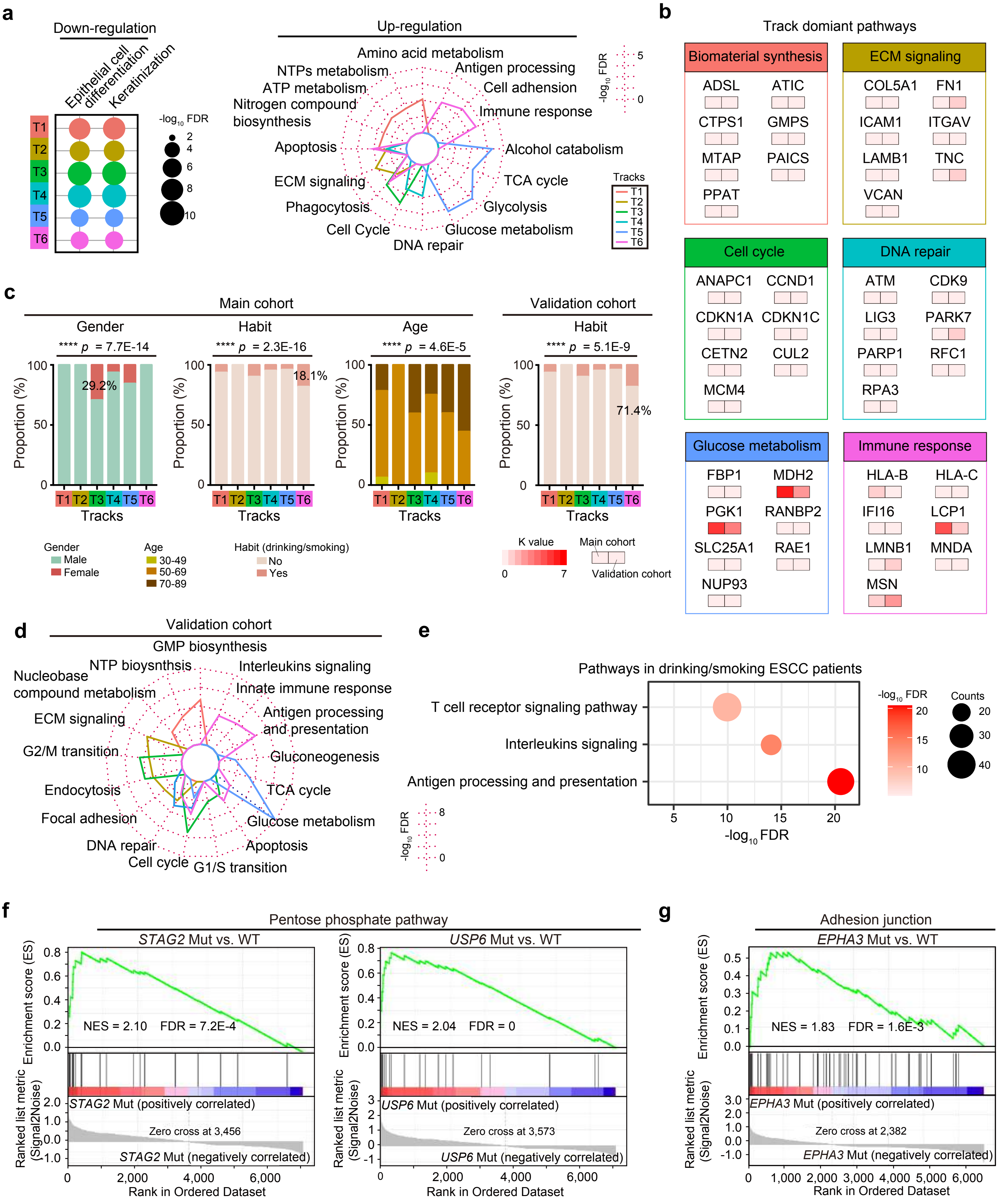

Supplementary Fig. 7 The trajectory analysis of the early ESCC cohort. (a) Radar analysis of the negative (left, K < 0) and positive pathways (right, K > 0) of the 6 tracks of the main cohort. **(b)** The represented proteins of the dominant pathways in the 6 major tracks of the main cohort (n = 746) and the validation cohort (n = 256). **(c)** Histograms depicting the proportion of different genders, drinking/smoking habits, and ages, in 6 tracks (two-sided Fisher's exact test). **(d)** Radar analysis of the tracks' dominant pathways in the validation cohort (n = 256). **(e)** The dominant pathways in the ESCC patients with the habit of drinking/smoking. Biological pathways are analyzed from the GO/KEGG database. **(f)** GSEA plots (KEGG gene sets) for pentose phosphate pathway in *STAG2* mutation and WT comparison (left) and *USP6* mutation and WT comparison (right). **(g)** GSEA plot (KEGG gene sets) for pentose phosphate pathway in *EPHA3* mutation and WT comparison. **** $p < 1.0E-4$, *** $p < 1.0E-3$, ** $p < 0.01$, * $p < 0.05$, ns. > 0.05. Source data are provided as a Source data file.

Supplementary Fig. 8

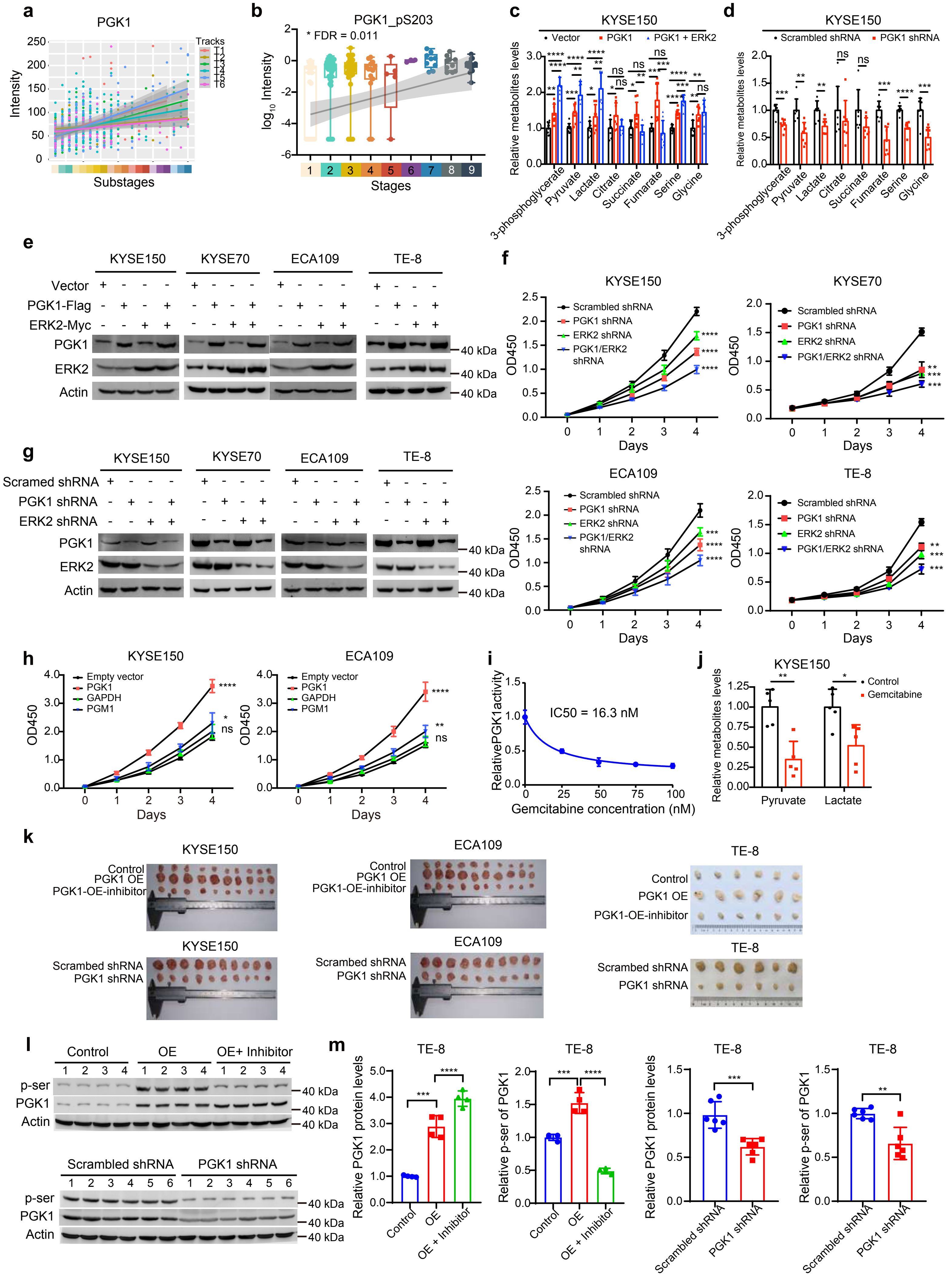

Supplementary Fig. 8 The impacts of GAPDH-OE or PGM1-OE on ESCC cell proliferation and inhibiting PGK1 by gemcitabine attenuates cell proliferation. (a) The expression of PGK1 in all 6 tracks. **(b)** Boxplot showing the expression of PGK1 S203 in the carcinogenesis progress of ESCC (Kruskal-Wallis test). Boxplot shows median (central line), upper and lower quartiles (box limits), 1.5× interquartile range (whiskers). A total of 145 samples were used for phosphoproteome. n (stage 1) = 20, n (stage 2) = 37, n (stage 3) = 31, n (stage 4) = 14, n (stage 5) = 5, n (stage 6) = 3, n (stage 7) = 9, n (stage 8) = 10, n (stage 9) = 16 biologically independent samples examined. **(c)** Metabolite levels in KYSE150 cells (n = 24) transfected with PGK1 or co-transfected with PGK1 and ERK2 (two-sided Student's t-test, mean ± SD). 3-phosphoglycerate: ** p = 2.0E-3 (PGK1), **** p = 1.6E-8 (PGK1 + ERK2), **** p = 2.7E-5 (ERK2 & (PGK1 + ERK2)). Pyruvate: *** p = 7.1E-4 (PGK1), **** p = 1.7E-5 (PGK1 + ERK2), ** p = 9.0E-3 (ERK2 & (PGK1 + ERK2)). Lactate: * p = 0.037 (PGK1), **** p = 1.5E-5 (PGK1 + ERK2), ** p = 1.4E-3 (ERK2 & (PGK1 + ERK2)). Citrate: * p = 0.038 (PGK1), p = 0.63 (PGK1 + ERK2), p = 0.055 (ERK2 & (PGK1 + ERK2)). Succinate: * p = 0.015 (PGK1), p = 0.30 (PGK1 + ERK2), ** p = 3.5E-3 (ERK2 & (PGK1 + ERK2)). Fumarate: *** p = 7.5E-4 (PGK1), p = 0.30 (PGK1 + ERK2), *** p = 8.0E-4 (ERK2 & (PGK1 + ERK2)). Serine: **** p = 2.3E-6 (PGK1), **** p = 5.3E-8 (PGK1 + ERK2), *** p = 3.2E-4 (ERK2 & (PGK1 + ERK2)). Glycine: ** p = 4.8E-3 (PGK1), ** p = 8.9E-3 (PGK1 + ERK2), p = 0.64 (ERK2 & (PGK1 + ERK2)). (**d)** Metabolite levels in PGK1-knockdown KYSE150 cells and control cells (two-sided Student's t-test, mean ± SD). Fourteen cell samples are used in the analysis. *** p = 3.9E-4, ** p = 2.1E-3, ** p = 4.2E-3, p = 0.37, p = 0.07, *** p = 3.6E-4, **** p = 2.5E-5, *** p = 6.8E-4 from left to right. **(e)** Western blot showing the impacts of PGK1-OE and ERK2-OE on the abundance of their counterpart proteins in the ESCC cells. **(f)** Cell proliferation with PGK1 knockdown and/or ERK2 knockdown in KYSE150 cells, KYSE70 cells, ECA109 cells, and TE-8 cell (two-sided Student's t-test, mean ± SD). A total of 320 cell samples are used in the analysis. KYSE150: **** p = 1.5E-7 (PGK1 shRNA), **** p = 1.2E-5 (ERK2 shRNA), **** p = 1.4E-8 (PGK1/ERK2 shRNA). KYSE70: ** p = 1.6E-3 (PGK1 shRNA), **** p = 6.4E-5 (ERK2 shRNA), **** p = 5.3E-5 (PGK1/ERK2 shRNA). ECA109: **** p = 2.4E-5 (PGK1 shRNA), **** p = 2.8E-4 (ERK2 shRNA), **** p = 1.0E-6 (PGK1/ERK2 shRNA). TE-8: ** p = 1.0E-3 (PGK1 shRNA), *** p = 4.7E-4 (ERK2 shRNA), *** p = 1.7E-4 (PGK1/ERK2 shRNA). **(g)** Western blot showing the impacts of PGK1 knockdown and/or ERK2 knockdown on the abundance of their counterpart proteins. **(h)** The impacts of

GAPDH-OE or PGM1-OE on cell proliferation in KYSE150 cells (left) and ECA109 cells (right) (two-sided Student's t-test, mean ± SD). PGK1-OE in KYSE150 cells (left) and ECA109 cells (right) is the positive control. A total of 200 cell samples are used in the analysis. KYSE150: **** p = 3.7E-7 (PGK1), p = 0.19 (GAPDH), * p = 0.026 (PGM1). ECA109: **** p = 1.9E-6 (PGK1), p = 0.17 (GAPDH), ** p = 1.6E-3 (PGM1). **(i)** *In vitro* assay showing the effects of gemcitabine on inhibition of PGK1 activity (IC50: 16.3 nM, mean ± SD). IC50, half-maximal inhibitory concentration. Bars represent the mean of n = 3 independent experiments with error bars indicating SD. **(j)** Gemcitabine decreased PGK1 mediated metabolic flux (two-sided Student's t-test, mean ± SD). Twenty cell samples are used in the analysis. ** p = 1.6E-3 (Pyruvate), * p = 0.014 (Lactate). **(k)** The impacts of PGK1-OE (top) and PGK1 knockdown (bottom) on the size of the xenografts in the KYSE150 cells, ECA109 cells, and TE-8 cells. **(l)** Western blot showing the abundance of PGK1 in the excited tumors at the protein and phosphoprotein levels (n = 3 independent experiments). **(m)** The column showing the impacts of PGK1 on the abundance of PGK1 at the protein and phosphoprotein levels in TE-8 cells (n = 48, two-sided Student's t-test, mean ± SD). *** p = 1.0E-4, **** p = 1.0E-6, *** p = 7.1E-4, **** p = 1.5E-5, *** p = 5.3E-4, ** p = 1.5E-3 from left to right. **** p < 1.0E-4, *** p < 1.0E-3, ** p < 0.01, * p < 0.05, ns. > 0.05. Source data are provided as a Source data file.

Supplementary Fig. 9

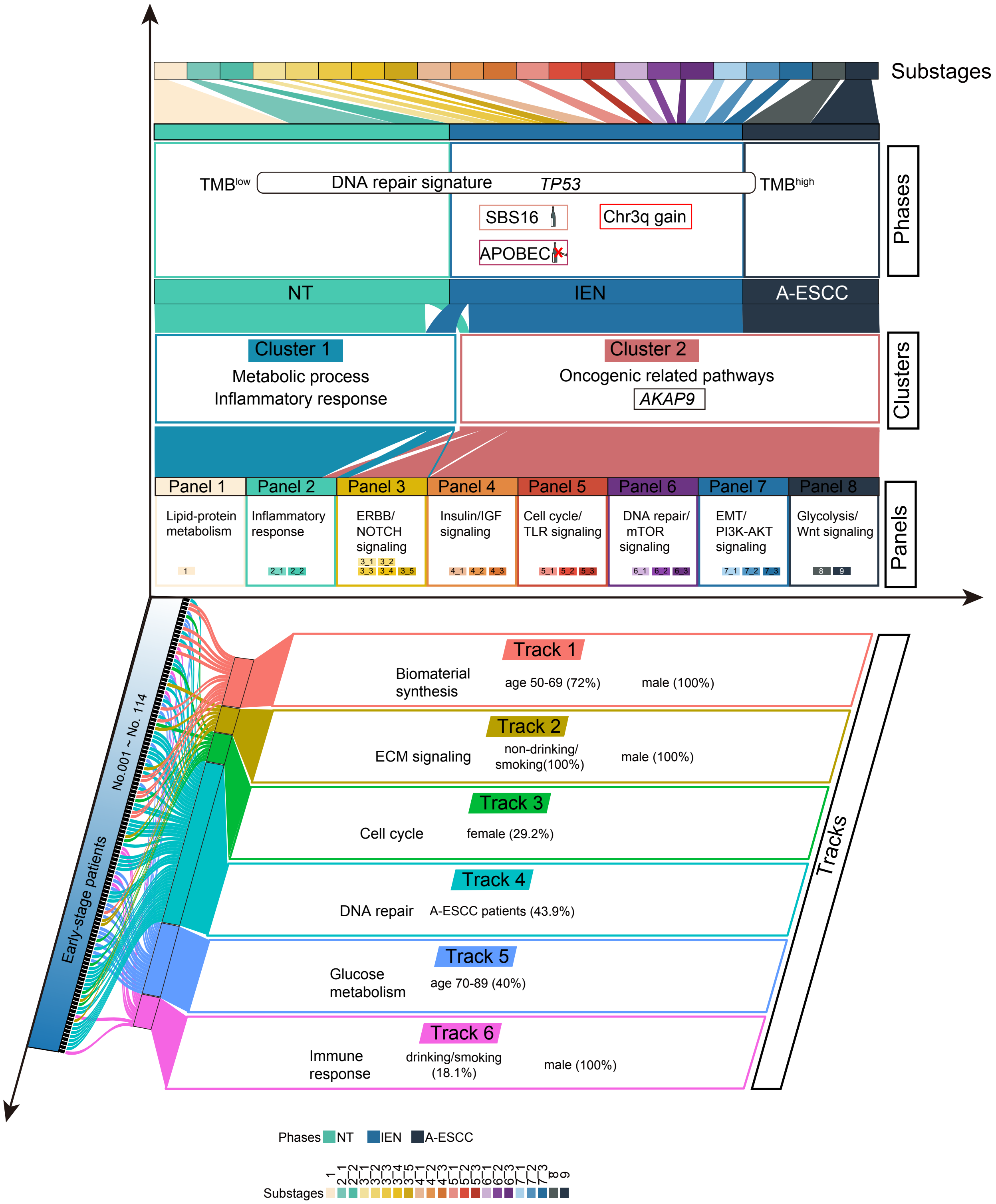

Supplementary Fig. 9 A brief summary of the characteristics of ESCC. TMB: tumor mutation burden.

Supplementary Table 1 | Clinical characteristics of Fudan early-stage ESCC cohort (n = 154).

**Characteristics**	**n (%)**
Age	
< 50 yr	12 (7.8)
50~70 yr	102 (66.2)
> 70 yr	40 (26.0)
Gender	
Male	140 (90.9)
Female	14 (9.1)
Habit (Drinking/smoking)	
Yes	19 (12.3)
No	135 (8.7)
TNM stage	
T1 stage	114 (74.0)
T2 stage	16 (10.4)
T3 stage	24 (15.6)
Survival status	
Alive	154 (100)
Death	0 (0)

Supplementary Table 2 | Subclassification information and samples number in the Fudan cohort.

Stages	Substages (sample number)
Stage 1 (Normal epithelial tissue)	1 (n = 114)
Stage 2 (Dysplasia stage)	2_1 (n = 114), 2_2 (n = 92)
Stage 3 (Tis stage)	3_1 (n = 61), 3_2 (n = 73), 3_3 (n = 67), 3_4 (n = 19), 3_5 (n = 39)
Stage 4 (Lamina propria cancer stage)	4_1 (n = 61), 4_2 (n = 18), 4_3 (n = 7)
Stage 5 (Muscularis mucosa stage)	5_1 (n = 14), 5_2 (n = 9), 5_3 (n = 9)
Stage 6 (Sm stage a)	6_1 (n = 5), 6_2 (n = 5), 6_3 (n = 7)
Stage 7 (Sm stage b)	7_1 (n = 12), 7_2 (n = 9), 7_3 (n = 11)
Stage 8 (T2 stage)	8 (n = 16)
Stage 9 (T3 stage)	9 (n = 24)

Supplementary Table 3 | Subclassification information and samples number of the validation cohort.

Stages	Substages (sample number)
Stage 1 (Normal epithelial tissue)	1 (n = 49)
Stage 2 (Dysplasia stage)	2_1 (n = 45), 2_2 (n = 30)
Stage 3 (Tis stage)	3_1 (n = 38), 3_2 (n = 27), 3_3 (n = 13), 3_4 (n = 7), 3_5 (n = 5)
Stage 4 (Lamina propria cancer stage)	4_1 (n = 20), 4_2 (n = 8), 4_3 (n = 1)
Stage 5 (Muscularis mucosa stage)	5_1 (n = 3), 5_2 (n = 3), 5_3 (n = 2)
Stage 6 (Sm stage a)	NA
Stage 7 (Sm stage b)	7_1 (n = 1), 7_2 (n = 4)

Supplementary Table 4 | Subclassification information and sample number of the Fudan cohort for phosphoproteome.

Stages	Substages (sample number)
Stage 1 (Normal epithelial tissue)	1 (n = 20)
Stage 2 (Dysplasia stage)	2_1 (n = 26), 2_2 (n = 11)
Stage 3 (Tis stage)	3_1 (n = 4), 3_2 (n = 11), 3_3 (n = 9), 3_4 (n = 2), 3_5 (n = 5)
Stage 4 (Lamina propria cancer stage)	4_1 (n = 9), 4_2 (n = 5)
Stage 5 (Muscularis mucosa stage)	5_2 (n = 1), 5_2 (n = 1), 5_3 (n = 3)
Stage 6 (Sm stage a)	6_2 (n = 1), 6_3 (n = 2)
Stage 7 (Sm stage b)	7_1 (n = 3), 7_2 (n = 3), 7_3 (n = 3)
Stage 8 (T2 stage)	8 (n = 10)
Stage 9 (T3 stage)	9 (n = 16)
