# Supplementary material for: Integrative proteogenomic characterization of early esophageal cancer
Source: Nat Commun. 2023 Mar 25;14:1666. doi: 10.1038/s41467-023-37440-w (PMC10039899; doi:10.1038/s41467-023-37440-w)
Supplement: Supplementary file 2 — Description of Additional Supplementary Files [file 41467_2023_37440_MOESM2_ESM.pdf]

Description of Supplementary Datasets:

File Name: **Supplementary Data 1**

Description: **Clinical characteristics of ESCC patients, Related to Fig. 1 and Supplementary Fig. 1. a**, The information of 154 ESCC cases (786 samples) for proteomic profiling; each sample has specific experiment ID at firmiana paltform. **b**, The information of screened 58 cases (145 samples) for phosphoproteomic profiling; each sample has specific experiment ID at firmiana paltform. **c**, The information of 46 cases (102 samples) for whole-exome sequencing. **d**, The HPV negative results of 30 early ESCC samples; AJ and Tellgen HPV were used.

File Name: **Supplementary Data 2**

Description: **Somatic mutations of 102 samples in ESCC progression, Related to Fig. 1, 2, and Supplementary Fig. 3, 4. a**, Somatioc mutations of 102 samples in ESCC progression.

File Name: **Supplementary Data 3**

Description: **The correlations analysis of 42 HEK293T cell samples and the identifies in ESCC progression at the protein and phosphoprotein levels, and the somatic copy number alterations of 102 samples in ESCC progression, Related to Fig. 3, 4, and Supplementary Fig. 1, 2, 4, 5. a**, The correlations analysis of 42 HEK293T cell samples as quality control. **b**, At least 2 unique peptides with 1% FDR at the peptide level and FOT $\geq$ 1.0E-5 (n = 10,913), NA was assigned as 1.0E-5. **c**, The identified phosphosites of 145 samples. **d**, Somatic copy number alterations of 102 samples in ESCC progression.

File Name: **Supplementary Data 4**

Description: **The gene effects of RNAi- and CRISPR-mediated depletion on ESCC cell lines and the cancer associated genes, Related to Fig. 3. a**, The gene effects of RNAi-mediated depletion on ESCC cell lines. **b**, The gene effects of CRISPR-mediated depletion on ESCC cell lines.

File Name: **Supplementary Data 5**

Description: **Proteome clusters of ESCC progression, Related to Fig. 5 and Supplementary Fig. 6. a**, Consensus cluster analysis of 786 samples in Fig. 5a. **b**, The differential expressed proteins in the two clusters.

File Name: **Supplementary Data 6**

Description: **Personalized trajectory of early ESCC, Related to Fig. 4, 6 and Supplementary Fig. 7. a**, The track samples in the main cohort. **b**, The value of slope index (K) of the equation in the main cohort. **c**, The value of coefficient index (R2) of the equation in the main cohort. **d**, The information of 49 ESCC cases (256 samples) for proteomic profiling in validation cohort. **e**, The value of slope index (K) and coefficient index (R2) of the equation in the validation cohort. **f**, At least 1 unique peptides with 1% FDR at the peptide level and FOT $\geq$ 1.0E-5 (n = 8,626) in the validation cohort, NA was assigned as 1.0E-5. **g**, The list of cancer-associated genes.

File Name: **Supplementary Data 7**

Description: **Aberrant glycolytic metabolism in ESCC and its key enzyme, PGK1, as a potential therapeutic target, Related to Fig 7,8, and Supplementary Fig. 8. a**, The overall survival information of PGK1. The data was downloaded from TCGA. **b**, Metabolites levels in

KYSE150 cells transfected with PGK1, or co-transfected with PGK1 and ERK2. **c**, Metabolites levels in PGK1-knockdown KYSE150 cells and control cells. **d**, The impacts of PGK1 and/or ERK2 on PDH activity in KYSE150 cells and ECA109 cells. **e**, The impacts of overexpressed PGK1 and ERK2 on OCR and ECAR. **f**, The impacts of knock-down PGK1 on OCR and ECAR. **g**, Cell proliferation in four kinds of ESCC cell lines (KYSE150, KYSE70, ECA109, and TE-8) with various treatments. **h**, Cell proliferation in four kinds of ESCC cell lines (KYSE150, KYSE70, ECA109, and TE-8) with PGK1 knockdown and/or ERK2 knockdown. **i**, Cell proliferation in GAPDH or PGM-knocking down- KYSE150 cells (left) and ECA109 cells (right). **j**, The effects of gemcitabine on inhibition of PGK1 activity (IC50: 16.3 nM). **k**, Gemcitabine decreased PGK1 mediated metabolic flux. **l**, Gemcitabine inhibited cell proliferation. **m**, The impacts of PGK1 on Tumor weight (g) in KYSE150 cells, ECA109 cells, and TE-8 cells xenografts with three groups: control group, PGK1-overexpression (OE) group, and PGK1-OE-inhibitor (gemcitabine) group. **n**, The impacts of PGK1 on tumor weight (g) of KYSE150 cells, ECA109 cells, and TE-8 cells xenografts with two groups: control group and PGK1-knockdown group. **o**, The impacts of PGK1 on the abundance of PGK1 at the protein and phosphoprotein levels in TE-8 cells.
