# Supplementary material for: Integrative proteogenomic characterization of early esophageal cancer
Source: Nat Commun. 2023 Mar 25;14:1666. doi: 10.1038/s41467-023-37440-w (PMC10039899; doi:10.1038/s41467-023-37440-w)
Supplement: Supplementary file 10 — Reporting Summary [file 41467_2023_37440_MOESM10_ESM.pdf]

nature portfolio

Corresponding author(s): Chen Ding
Yingyong Hou
Jian-Yuan Zhao
Pinghong Zhou

Last updated by author(s): Mar 15, 2023

Reporting Summary

Nature Portfolio wishes to improve the reproducibility of the work that we publish. This form provides structure for consistency and transparency in reporting. For further information on Nature Portfolio policies, see our Editorial Policies and the Editorial Policy Checklist.

Statistics

For all statistical analyses, confirm that the following items are present in the figure legend, table legend, main text, or Methods section.

n/a	Confirmed	
☐	☒	The exact sample size (*n*) for each experimental group/condition, given as a discrete number and unit of measurement
☐	☒	A statement on whether measurements were taken from distinct samples or whether the same sample was measured repeatedly
☐	☒	The statistical test(s) used AND whether they are one- or two-sided<br>*Only common tests should be described solely by name; describe more complex techniques in the Methods section.*
☐	☒	A description of all covariates tested
☐	☒	A description of any assumptions or corrections, such as tests of normality and adjustment for multiple comparisons
☐	☒	A full description of the statistical parameters including central tendency (e.g. means) or other basic estimates (e.g. regression coefficient) AND variation (e.g. standard deviation) or associated estimates of uncertainty (e.g. confidence intervals)
☐	☒	For null hypothesis testing, the test statistic (e.g. *F*, *t*, *r*) with confidence intervals, effect sizes, degrees of freedom and *P* value noted<br>*Give P values as exact values whenever suitable.*
☒	☐	For Bayesian analysis, information on the choice of priors and Markov chain Monte Carlo settings
☐	☒	For hierarchical and complex designs, identification of the appropriate level for tests and full reporting of outcomes
☐	☒	Estimates of effect sizes (e.g. Cohen's *d*, Pearson's *r*), indicating how they were calculated

Our web collection on statistics for biologists contains articles on many of the points above.

Software and code

Policy information about availability of computer code

Data collection	The proteomic raw data were performed by Q Exactive HF-X Mass Spectrometer, Thermo Fisher Scientific. The phosphoproteomic raw data were performed by Q Exactive HF-X Mass Spectrometer, Thermo Fisher Scientific. The whole exon sequencing data were obtained through, Illumina Novaseq 6000. Data of the mitochondrial respiration oxygen consumption rate and extracellular acidification rate were collected on a Seahorse XF96 Extracellular Flux Analyzer (Agilent Technologies, CA, USA).
Data analysis	The data analysis was performed by programming language R (version 4.0.2) and GraphPad Prism (version 9.0). Most of them used broadly applied R packages and others used self-made R scripts according published papers: ConsensusClusterPlus (v1.50.0), pheatmap (v1.0.12) for supervised hierarchical clustering, Hmisc (v4.5-0) for spearman's correlation calculating, ggplot2 (v3.3.5) for scatter plot. For WES, BWA (v0.7.12, Li H et al.), SAMtools (v1.9, Li H et al.) and Picard (http://broadinstitute.github.io/picard/) were used to genome alignment, and muTect Software (Cibulskis K et al. 2013) was used for targeting Somatic SNV sites, and Strelka was used to test Somatic INDEL information. Valid sequencing data was mapped to the reference human genome (UCSC hg19) by Burrows-Wheeler Aligner (BWA, v0.7.12) software to get the original mapping results stored in BAM format. SAMtools (v1.9) and Picard (http://broadinstitute.github.io/picard/) were used to sort BAM files and do duplicate marking, local realignment, and base quality recalibration to generate final BAM file for computation of the sequence coverage and depth. Somatic variants were then called, utilizing VarScan v2.3.8, MuTect v1.1.7) and InVEX (http://www.broadinstitute.org/software/invex/). SCNA analysis was performed by following somatic copy-number variation (CNV) calling pipeline in GATK's (GATK v 4.1.2.0) Best Practice. The results of this pipeline, segment files of every 1,000, were put in GISTIC2 (v2.0). Reads were mapped onto the human reference genome (GRCh38.p13 assembly) by using STAR software (v2.7.7a). The mapped reads were assembled into transcripts or genes by using StringTie software (v2.1.4) and the genome annotation file (hg38_ucsc.annotated.gtf). SCNAs affecting protein and phosphoprotein abundance in either "cis" (within the same aberrant locus) or "trans" (remote locus) mode were visualized using "multiOmicsViz" (v1.18.0) R package. Kinase activity scores were inferred from phosphorylation sites by employing PTM signature enrichment analysis (PTM-SEA) using the PTM signatures database (PTMsigDB) v1.9.0 (https://github.com/broadinstitute/ssGSEA2.0). Standard statistical tests in this study were

used to analyze the clinical data, including but not limited to Wilcoxon signed-rank test, Fisher's exact test, Kruskal-Wallis test. Standard statistical tests were used to analyze the clinical data, including but not limited to Wilcoxon signed-rank test, Fisher's exact test, Kruskal-Wallis test. Specifically, the statistical significance of differences between two groups was calculated with the Wilcoxon rank-sum test and Student's t-test; for more than two groups comparison, Kruskal–Wallis test was used. When exploring the association of different groups with clinical variables, Fisher's exact test and Wilcoxon rank-sum test were used for categorical variables and continuous variables, respectively. As for the correlation analysis between two proteins/phosphoproteins, Pearson's correlation of correlation coefficients was used. For the correlation analysis among the stages in ESCC progression and different HEK293T cell samples, Spearman's correlation of correlation coefficients were used. Kaplan–Meier plots (two-sided log-rank test) were used to describe the OS. For validating the findings in this study, each experiment was repeated at least three times independently. In this study, all analyses were performed in R (version 4.0.2) and GraphPad Prism (Version 9), and all statistical tests were two-sided, and statistical significance was considered when p value < 0.05, which were adjusted using the BH procedure. Data in the boxplot were presented median (central line), upper and lower quartiles (box limits), 1.5× interquartile range (whiskers).

For manuscripts utilizing custom algorithms or software that are central to the research but not yet described in published literature, software must be made available to editors and reviewers. We strongly encourage code deposition in a community repository (e.g. GitHub). See the Nature Portfolio guidelines for submitting code & software for further information.

Data

Policy information about availability of data

All manuscripts must include a data availability statement. This statement should provide the following information, where applicable:

- Accession codes, unique identifiers, or web links for publicly available datasets
- A description of any restrictions on data availability
- For clinical datasets or third party data, please ensure that the statement adheres to our policy

The proteome and phosphoproteome raw datasets generalized in this study have been deposited to the ProteomeXchange Consortium (dataset identifier: PXD038961) via the iProX partner repository (https://www.iprox.cn/) under Project ID IPX0002178000. The VCF files of the WES data files were deposited to the European Genome-Phenome Archive (EGA) associated with the study EGAS00001006126 under project ID EGAD00001008672. Data is available upon request through EGA without any restrictions, and will be available permanently. The raw WES raw are availability in the GSA (Genome Sequence Archive, https://ngdc.cncb.ac.cn/gsa-human/) under restricted access HRA004153 for data privacy laws related to patient consent for data sharing, access can be obtained by the Request Data steps in GSA database website or contacting corresponding author. The approximate response time for accession requests is about 2 weeks. Once access has been granted, the data will be available to download for 3 months. The gene expression profiles of ESCC cell lines in public dataset Expression 21Q2 in this study are available in the Depmap database (https://depmap.org/portal/download/?releasename=DepMap+Public+21Q2&filename=CCLE_expression.csv). The remaining data are available within the Article, Supplementary Information, or Source Data file. Source data are provided with this paper.

Human research participants

Policy information about studies involving human research participants and Sex and Gender in Research.

Reporting on sex and gender	A total of 154 esophageal squamous cell carcinoma (ESCC) patients were enrolled in this study, in which 141 male and 13 female ESCC patients were included.
Population characteristics	The perspective cohort consisted of 141 male and 13 female patients, and had a median age of 63 years old, 19 and 135 of which had a habit of drinking/smoking or not.
Recruitment	Three hundred consecutive esophageal squamous cell carcinoma (ESCC) patients were presumed to have esophageal lesions underwent ESD therapy from January 2018 to December 2018 at Zhongshan Hospital, Fudan University. There were no biases in selecting patients, and none of the patients had received any prior treatment, such as radiotherapy or chemotherapy. One hundred and fourteen early ESCC cases were eligible for the establishment of the intended study cohort. Among the 186 excluded patients, 21 were diagnosed with non-tumor lesions, 26 had stromal tumors, 86 patients were precluded due to unavailability of their normal tissue samples, and 53 samples failed to pass the pathological quality check, such as tumor cell ratio < 80%. Subsequently, 40 advanced ESCC cases (n = 16 for T2 and 24 for T3) were screened after surgical resection without neoadjuvant therapy. Finally, a total of 154 ESCC patients were enrolled in this study, in which 141 male and 13 female ESCC patients were included.
Ethics oversight	The present study was carried out in compliance with the ethical standards of Helsinki Declaration II and approved by the Institution Review Board of Fudan University Zhongshan Hospital (B2019-200R).

Note that full information on the approval of the study protocol must also be provided in the manuscript.

Field-specific reporting

Please select the one below that is the best fit for your research. If you are not sure, read the appropriate sections before making your selection.

☒ Life sciences ☐ Behavioural & social sciences ☐ Ecological, evolutionary & environmental sciences

For a reference copy of the document with all sections, see nature.com/documents/nr-reporting-summary-flat.pdf

Life sciences study design

All studies must disclose on these points even when the disclosure is negative.

Sample size	The proteomic profiling was performed on the 786 tissues samples from 154 esophageal squamous cell carcinoma (ESCC) cases.<br>The phosphoproteomic analysis was performed on the 145 tissues samples from 58 ESCC cases.<br>The WES analysis was conducted on the 102 tissues samples from 46 ESCC cases.<br>No statistical method was used to predetermine sample sixze.<br>The functional and biological experiments were performed with at least three biological replicates to allow statistical significance testing through two-sided student's t-test.
Data exclusions	Cases for MS screening as following: 300 consecutive patients that were presumed to have esophageal lesion were undergo an ESD therapy from January to December 2018 in Zhongshan Hospital, Fudan University. And 114 early ESCC cases were eligible for intended study cohort. Among the 186 excluded patients, 47 ones were excluded at first, 21 among whom 21 were diagnosed as non-tumor lesions, and 26 were diagnosed as stromal tumors. Among the 253 early ESCC and precancerous lesions, 86 patients were precluded due to their unavailable normal tissues, and 53 samples failed to pass pathological quality check, such as tumor cells rate < 80%). Then, 40 advanced ESCC cases (16 in T2 stage and 24 in T3 stage) were screened after surgical resection without neoadjuvant therapy.<br>Case for phosphoproteome dataset: only 154 samples from 58 ESCC patiens were adequate and chosen from 786 samples.<br>Cases for genome dataset: only 102 samples in 46 ESCC patiens were adequate and chosen from 786 samples.
Replication	All experiments were reliably reproduced and indicated in figure legends. The replicated analysis of 293T cell lysates were used for the quality control of the mass spectrometer.
Randomization	For multi-omic analysis, samples of ESCC patients were randomly divided into groups to avoid bias for protein/phosphoprotein quantification.
Blinding	The investigators who measured protein/phosphoprotein expression, whole-exome sequencing (WES) data were blinded to patient information. The investigators who performed IHC were blinded to clinical information of esophageal squamous cell carcinoma (ESCC) patients. For consensus clustering analyses, the investigators were blinded to group allocation during data collection.

Reporting for specific materials, systems and methods

We require information from authors about some types of materials, experimental systems and methods used in many studies. Here, indicate whether each material, system or method listed is relevant to your study. If you are not sure if a list item applies to your research, read the appropriate section before selecting a response.

Materials & experimental systems

n/a	Involved in the study	
☐	☒	Antibodies
☐	☒	Eukaryotic cell lines
☒	☐	Palaeontology and archaeology
☐	☒	Animals and other organisms
☒	☐	Clinical data
☒	☐	Dual use research of concern

Methods

n/a	Involved in the study	
☒	☐	ChIP-seq
☒	☐	Flow cytometry
☒	☐	MRI-based neuroimaging

Antibodies

Antibodies used	Western blot analysis:<br>Anti-PGK1 antibody (Wuhan Fine Biotech Co., Ltd., China, Catalog: FNab06354, dilution 1:1000),<br>Anti-β-actin (Genscript, Piscataway, NJ, USA, Catalog: A00702, dilution 1: 10000),<br>Anti-p-Ser (Cell Signaling Technology, Danvers, MA, USA, Catalog:9606, dilution 1: 4000),<br>Anti-phospho-Threonine (Cell Signaling Technology, Danvers, MA, USA, Catalog: 9386, dilution 1: 1000),<br>Anti-phospho-Tyrosine (Cell Signaling Technology, Danvers, MA, USA, Catalog: 9411, dilution 1: 2000),<br>Anti-COX IV (Cell Signaling Technology, Danvers, MA, USA, Catalog: 4580, dilution 1: 1000), Anti-GAPDH (Cell Signaling Technology, Danvers, MA, USA, Catalog: 85925, dilution 1: 10000),<br>Anti-Thr-338 PDHK1 (Signalway Antibody, Nanjing, China, Catalog: C11596, dilution 1: 500), Anti-PDHK1 (Cell Signaling Technology, Danvers, MA, USA, Catalog: 3820, dilution 1: 1000), Anti-ERK2 (Cell Signaling Technology, Danvers, MA, USA, Catalog: 9108, dilution 1: 1000), Anti-Flag (Abmart, Shanghai, China, Catalog: M20008, dilution 1: 5000),<br>Anti-PGM1 (Cell Signaling Technology, Danvers, MA, USA, Catalog: 12098, dilution 1: 1000), Anti-PHGDH (Cell Signaling Technology, Danvers, MA, USA, Catalog: 66350, dilution 1: 1000).<br><br>Immunohistochemistry analysis:<br>Anti- PGK1 polyclonnal antibody was also validated for IHC (1:500 dilution).
Validation	All antibodies used in this manuscript were obtained from the indicated commercial vendors and have been validated by the respective manufacturer, as described in their website.

Anti-PGK1 (Wuhan Fine Biotech Co., Ltd., China, Catalog: FNab06354, dilution 1:1000) validated for immunohistochemistry (IHC) and western blotting by manufacturer [http://www.finebio.cn/finebio-Products/D11580260.html.],
Anti-β-actin (Genscript, Piscataway, NJ, USA, Catalog: A00702, dilution 1: 10000) validated for western blotting by manufacturer [https://www.genscript.com.cn/antibody/A00702THE_beta_Actin_Antibody_mAb_Mouse.html.],
Anti-p-Ser (Cell Signaling Technology, Danvers, MA, USA, Catalog: 9606, dilution 1: 4000) validated for western blotting by manufacturer [https://www.cellsignal.cn/products/primary-antibodies/phospho-ser-14-3-3-binding-motif-4e2-mouse-mab/9606?site-search-type=Products&N=4294956287&Ntt=phospho-serine&fromPage=plp],
Anti-phospho-Threonine (Cell Signaling Technology, Danvers, MA, USA, Catalog: 9386, dilution 1: 1000) validated for western blotting by manufacturer [https://www.cellsignal.cn/products/primary-antibodies/phospho-threonine-42h4-mouse-mab/9386?site-search-type=Products&N=4294956287&Ntt=anti-phospho-threonine&fromPage=plp.],
Anti-phospho-Tyrosine (Cell Signaling Technology, Danvers, MA, USA, Catalog: 9411, dilution 1: 2000) validated for western blotting by manufacturer [https://www.cellsignal.cn/products/primary-antibodies/phospho-tyrosine-mouse-mab-p-tyr-100/9411?site-search-type=Products&N=4294956287&Ntt=phospho-tyrosine&fromPage=plp.],
Anti-COX IV (Cell Signaling Technology, Danvers, MA, USA, Catalog: 4580, dilution 1: 1000) validated for western blotting by manufacturer [https://www.cellsignal.cn/products/primary-antibodies/cox-iv-3e11-rabbit-mab/4850?site-search-type=Products&N=4294956287&Ntt=anti-cox+iv&fromPage=plp.],
Anti-GAPDH (Cell Signaling Technology, Danvers, MA, USA, Catalog: 85925, dilution 1: 10000) validated for western blotting by manufacturer [https://www.cellsignal.com/products/primary-antibodies/gapdh-14c10-rabbit-mab-bsa-and-azide-free/85925?site-search-type=Products&N=4294956287&Ntt=gapdh&fromPage=plp.],
Anti-Thr-338 PDHK1 (Signalway Antibody, Nanjing, China, Catalog: C11596, dilution 1: 500) validated for western blotting by manufacturer [https://www.sabbiotech.cn/g-190064-PDHK1(Phospho-Thr338)-Conjugated-Antibody-C11596.html.],
Anti-PDHK1 (Cell Signaling Technology, Danvers, MA, USA, Catalog: 3820, dilution 1: 1000) validated for western blotting by manufacturer [https://www.cellsignal.com/products/primary-antibodies/pdhk1-c47h1-rabbit-mab/3820?site-search type=Products&N=4294956287&Ntt=anti-pdhk1&fromPage=plp&_requestid=218510],
Anti-ERK2 (Cell Signaling Technology, Danvers, MA, USA, Catalog: 9108, dilution 1: 1000) validated for western blotting by manufacturer [https://www.cellsignal.com/products/primary-antibodies/p42-map-kinase-erk2-antibody/9108?site-search-type=Products&N=4294956287&Ntt=anti-erk2&fromPage=plp],
Anti-Flag (Abmart, Shanghai, China, Catalog: M20008, dilution 1: 5000) validated for western blotting by manufacturer [http://www.ab-mart.com.cn/page.aspx?node=%2059%20&id=%20968],
Anti-PGM1 (Cell Signaling Technology, Danvers, MA, USA, Catalog: 12098, dilution 1: 1000) validated for western blotting by manufacturer [https://www.cellsignal.com/products/primary-antibodies/pgam1-d3j9t-rabbit-mab/12098?site-search-type=Products&N=4294956287&Ntt=anti-pgm&fromPage=plp.],
Anti-PHGDH (Cell Signaling Technology, Danvers, MA, USA, Catalog: 66350, dilution 1: 1000) validated for western blotting by manufacturer [https://www.cellsignal.com/products/primary-antibodies/phgdh-d8f3o-rabbit-mab/66350?site-search-type=Products&N=4294956287&Ntt=phgdh&fromPage=plp.].

Eukaryotic cell lines

Policy information about cell lines and Sex and Gender in Research

Cell line source(s)	HEK293T cells (ATCC, Catalog: CRL-11268, RRID: CVCL_QW54), Human ECA109 cells (ATCC, Catalog: GCC-OE0002CS, RRID: CVCL_6898), Human KYSE150 cells (ATCC, Catalog: GCC-OE0004CS, RRID: CVCL_QW54), Human KYSE70 cells (YaJi Biological, Catalog: YS1331C, RRID: CVCL_1356), Human TE-8 cells (YaJi Biological, Catalog: YS2958C, RRID: CVCL_1766).
Authentication	All the cell lines were authenticated with short tandem repeat (STR) profiling method.
Mycoplasma contamination	All cell lines tested negative for mycoplasma contamination.
Commonly misidentified lines (See ICLAC register)	No commonly misidentified cell lines were used.

Animals and other research organisms

Policy information about studies involving animals; ARRIVE guidelines recommended for reporting animal research, and Sex and Gender in Research

Laboratory animals	Five-week-old male Balb/C nude mice were obtained (Shanghai SLAC Laboratory Animal Co., Ltd, Shanghai, China) for in vivo xenografts. ESCC cells (1×10^7) were subcutaneously injected into nude mice (n=10 mice/condition); tumors were harvested and weighed after 30 days post-injection. Mice were housed in polycarbonate cages, and provided free access to food and water with a 12-h light:dark cycle.
Wild animals	No wild animals were involved.
Reporting on sex	A total of 130 BALB/c nude mice (male) were obtained in this study.
Field-collected samples	No field-collected samples were involved.
Ethics oversight	All experimental procedures involving animals were approved by the Fudan University Institutional Animal Care and Use Committee and were conducted in accord with the National Institutes of Health Guidelines for the Care and Use of Laboratory Animals.

Note that full information on the approval of the study protocol must also be provided in the manuscript.
